# The rise of gemination in Celtic

**DOI:** 10.12688/openreseurope.15400.1

**Published:** 2023-02-02

**Authors:** David Stifter

**Affiliations:** 1 Early Irish, Maynooth University, Maynooth, Kildare, W23 F2H6, Ireland

**Keywords:** Indo-European linguistics, Celtic linguistics, historical linguistcs, geminate consonants, substrate linguistics

## Abstract

This study investigates systematically the emergence and establishment of geminate conson­ants as a phono­logical class in the Celtic branch of Indo-European. The approach of this study is comparative historical linguistics, drawing on diachronic structuralism combined with aspects of language contact studies and functional approaches to language usage. This study traces the development of geminates from Proto-Indo-European (fourth millennium b.c.), which did not allow geminate consonants, to the Common Celtic period (first millennium b.c.), when almost every consonant could occur as a singleton or as a geminate, and on to the earliest attested stages of the Insular Celtic languages (1 ^st^ millennium a.d.). Although they were prominent in the phonology of Proto- and Ancient Celtic (Gaulish, Celtiberian), ultimately geminates were gotten rid of as a phonological class in the individual Insular Celtic languages. This is probably due to the fact that the contrast between lenited and unlenited sounds took on a central role in Insular Celtic phonology, making gemination a redundant category.

Most instances of geminate consonants in Celtic can be explained by regular sound change operating on inherited clusters of consonants. Each sound change will be discussed in a separate section in a rough chronological order. Effectively, gemination is largely a strategy to reduce the number of allowed consonant combinations. To a limited degree, gemination also had a morphological function, especially in the formation of personal names and in the creation of adjectival neologisms. However, there is a residue of words, especially nouns, in the Insular Celtic languages that defy any attempt at etymologising. They are prime suspects of having been borrowed from prehistoric, substratal languages.

## Plain language summary

Geminate, *i.e.,* ‘double’ or ‘long’, consonants were very common in Proto- and Ancient Celtic languages, such as Gaulish or Celtiberian of the 1 ^st^ millennium B.C. and earlier. They were also very prominent in the prehistory of the Insular Celtic languages, *e.g.* Irish, Welsh or Breton, but they were abandoned as a class of sounds shortly before the attestation of those languages due to other developments in those languages, especially the rise of lenited sounds as a grammatically very important class. This important role of geminates in Celtic contrasts with the situation in its ancestor language, reconstructed Proto-Indo-European (ca. middle 4 ^th^ millennium B.C.), which effectively disallowed geminate consonants. This article explains, how geminate consonants arose step by step in the prehistory and the early history of Celtic, mostly by regular sound change operating on inherited words. In addition, gemination became prominent in the formation of personal names and in the creation of new adjectives. However, a group of nouns with geminates finds no explanation within the traditional framework of historical linguistics. It is suggested that they are due to borrowings from prehistoric, lost languages in the west of Europe.

## Abbreviations

**Table:** 

acc.	accusative
Bret.	Breton
Cib.	Celtiberian, c. 150–0 b.c.
CIIC	*Corpus Inscriptionum Insularum Celticarum*, *i.e.* Macalister, 1945
CisGaul.	Cisalpine Gaulish, second–first centuries b.c.
Corn.	Cornish
dat.	dative
eDIL	*electronic Dictionary of the Irish Language*, *i.e.* Toner *et al*., 2019
fem.	feminine
Fr.	French
Gall.-Lat.	Gallo-Latin (Latin spoken in Gaul)
Gaul.	Gaulish, second century b.c.– c. fourth century a.d.
gen.	genitive
Germ.	German
GOI	*A Grammar of Old Irish*, i.e. Thurneysen, 1946
GPC	*Geiriadur Prifysgol Cymru* (Dictionary of the University of Wales)
Gr.	Ancient Greek
Hisp.- Lat.	Hispanic Latin, Latin in the Iberian Peninsula
IEW	*Indogermanisches Etymologisches Wörterbuch*, *i.e.* Pokorny, 1959
Ital.	Italian
Lat.	Latin
Latv.	Latvian
LCorn.	Late Cornish, early eighteenth century
LEIA	*Lexique Étymologique de l’Irlandais Ancien*, i.e. Vendryes *et al*., 1959–
LGaul.	Late Gaulish, c. second–fourth centuries a.d.
Lith.	Lithuanian
LIV	*Lexikon der Indogermanischen Verben*², i.e. Kümmel & Rix, 2001
masc.	masculine
MBret.	Middle Breton, c. 1100–1650
MCorn.	Middle Cornish, c. 1200–1600
MHG	Middle High German, c. 1050–1500
MIr.	Middle Irish, c. 900–1200
MLat.	Middle Latin, Latin in the Middle Ages
MLG	Middle Low German, c. 1200–1600
ModIr.	Modern Irish, c. 1200–present
MW	Middle Welsh, c. 1100–1400
NBret.	New (or Modern) Breton, c. 1650–present
neut.	neuter
NIL	*Nomina im Indogermanischen Lexikon*, i.e. Wodtko, Irslinger & Schneider 2008
nom.	nominative
OCorn.	Old Cornish, c. 800–1200
OCS	Old Church Slavonic, c. 860–1100
OEngl.	Old English, c. mid-seventh–eleventh centuries
OFr.	Old French, c. ninth–fourteenth centuries
OHG	Old High German, c. 750–1050
OIr.	Old Irish, c. 700–900
ON	Old Norse, c. 800–1350
OW	Old Welsh, c. 800–1100
PC	Proto-Celtic, c. late second millennium b.c.
Pict.	Pictish
PIE	Proto-Indo-European, c. middle of the fourth millennium b.c.
pl.	plural
pres.	present
PrimIr.	Primitive Irish, c. fourth–sixth centuries a.d.
qPC	quasi-Proto-Celtic
qPIE	quasi-Proto-Indo-European
RIIG	*Recueil informatisé des inscriptions gauloises*, i.e. Ruiz Darasse *et al*., 2022
Sc. Gael.	Scottish Gaelic
sg.	singular
subj.	subjunctive
VKG	*Vergleichende keltische Grammatik*, *i.e*. Pedersen, 1909–1913.
VLat.	Vulgar Latin
VN	verbal noun
voc.	vocative
W	Welsh (any stage of the language, but especially the modern variant after 1400)
1/2/3sg./ pl.	first, second, third person singular or plural

## Preliminaries

This study investigates systematically the emergence and establishment of geminate consonants as a phonological class in the Celtic branch of Indo-European. The aim is to come closer to a general theory of this phenomenon in Celtic, in order to permit inferences about the etymology of words that contain geminate consonants in the attested Celtic languages. Geminate sounds have been commonly used as an argument for identifying borrowings from unknown substrate languages. However, before their precise diachronic and synchronic status within the phonological system of Proto-Celtic and the individual languages hasn’t been determined, conclusions about layers of loanwords based on geminates are circular.

The approach of this study is diachronic structuralism combined with aspects of language contact studies and of functional approaches to language usage. The working hypothesis is that the inexorable, albeit gradual, rise of geminate consonants as a phonological class across all modes of articulation in the older stages of Celtic was a multicausal process that was fed by a combination of language-internal developments in Celtic and of language-external factors. The focus of this article is on the period up to the beginning of the 1 ^st ^millennium a.d. Evidently younger sound changes that occurred in the documented histories of Irish or British and that created further instances of geminates or, at that stage, fortes sounds (for example, assimilations that postdate syncope such as *nl* > *ll* in OIr. (Old Irish) *tenlach* > *tellach* ‘hearth’, *ld* > *ll* in *acaldam* > *acallam* ‘conversation, dialogue’, or MacNeill’s Law in Irish, or provection after syncope in British, or other late sound laws) are not treated here. Reference to them will only be made when they are relevant to clarify points in the prehistoric developments.

The terminology in this article follows the traditional practice in Celtic historical phonology. The term ‘geminate consonant’ will be used synonymously with ‘long consonant’. Geminate consonants are written double in reconstructions, *e.g.*, * *ballo-*, which is meant as equivalent to a phonetic analysis as [balːo-]. The main contrast between the two classes of Celtic stops is considered to be between ‘voiced’ (= D) and ‘unvoiced/voiceless’ (= T) consonants. Phonetically, the contrast between the D-series and the T-series may rather have been that between ‘lenis’ voiceless consonants and ‘fortis’ aspirated voiceless consonants (cf. Stifter, 2017: 1191; similar Van Sluis, 2019: 3–36; sceptical Martinet, 1952: 201) or, in Eska’s (2018) framework of Laryngeal Realism, a contrast in the feature [spread glottis]. Since the sound changes in this article are understood as arithmetic abstractions, notations of reconstructions can usually be transferred easily between alternative frames of references. The phonetic details of these variant descriptions do not make a practical difference here. In addition, it is assumed that intervocalic voiced stops developed non-contrastive, lenited, *i.e.*, fricative allophones already at a very early stage (cf. Schrijver, 2016: 497–499; Stifter, 2017: 1189–1190). These fricative allophones are not indicated in the reconstructions.

The label ‘PIE’ (Proto-Indo-European) will be reserved for reconstructions that can be securely set up for the protolanguage, while ‘qPIE’ (quasi-Proto-Indo-European) is an umbrella label for any *voreinzelsprachlich*, pre-Celtic reconstruction that may have arisen in the long period between the break-up of Proto-Indo-European and the emergence of Proto-Celtic (PC), for instance during an extended Western Indo-European period. qPIE reconstructions will be given in standard PIE phonology, even where this may be anachronistic. *Mutatis mutandis*, similar considerations apply to the label ‘qPC’ (quasi-Proto-Celtic).

Examples are cited from the standard lexicographic collections. As a rule, no special reference to these sources is made and etymologies are only discussed in cases where it is necessary. The reference points for Proto-Indo-European are *Lexikon der Indogermanischen Verben* (LIV = Kümmel & Rix, 2001), *Nomina im Indogermanischen Lexikon* (NIL = Wodtko, Irslinger & Schneider 2008) and *Indogermanisches Etymologisches Wörterbuch* (IEW = Pokorny, 1959). For Proto-Celtic, the standard handbooks are Matasović’s *Etymological Dictionary of Proto-Celtic* (2009) and Schumacher’s (2004) lexicon of primary verbs, *Die keltischen Primärverben*. Old Irish words are cited from the *electronic Dictionary of the Irish Language* (eDIL = Toner *et al*., 2019; albeit with occasional spelling normalisation to an idealised Old Irish standard; for criticism of the inconsistent spelling of headwords in eDIL see Griffith *et al*., 2018: 7), Welsh and Cornish from *Geiriadur Prifysgol Cymru* ( GPC Online), and Breton from Favereau (2016). Gaulish data is taken from Delamarre (2003) and from Delamarre (2007) for personal names; Celtiberian data from Jordán Cólera (2019). Lepontic and Cisalpine Celtic examples are taken from *Lexicon Leponticum* ( Stifter *et al*., 2009). Ancient Celtic personal names are cited either as abstract stems or in an idealised nominative singular, even if that form is unattested. Since this article is concerned with the predesinential parts of words, this procedure has no consequence for the main argument. Due to the nature of the sources, the semantics of ancient Celtic cognates is occasionally uncertain. The position adopted here is that the choice of ancient cognates is guided by plausibility, *i.e.*, formal phonological and morphological correspondence with words in other languages. If new evidence should emerge that casts doubt on these equations, the items would have to be removed.

This study consists of two big conceptual parts. Chapters 2–8 are devoted to etymological gemination, *i.e.*, types of gemination that arose by regular sound change operating on inherited words. The sections within this first part are arranged in an approximate, but not strict, chronological order. The principle of thematic coherence occasionally overrides chronology. For instance, morphological processes such as gemination arising from inflection, derivation and compounding are treated in single sections, even if they may relate to extended periods of time. The second part of the study is concerned with non-etymological gemination. ‘Non-etymological’ means that such types or instances of gemination cannot be described by regular sound laws, but they arose non-predictably from pragmatic contexts such as addressing, through sound-symbolic neologisms, or are due to borrowing.

### Previous research

The first dedicated study of geminates in a Celtic language was in response to an observation in Germanic linguistics, namely that obstruents before * *n* seem to have become geminates in Proto-Germanic. This is known as ‘Kluge’s Law’ (proposed by Kluge, 1884; for a modern take, see Kroonen, 2013: xxxiv–xxxv). Stokes ( 1891; revised in 1891–3) took his inspiration from this recently postulated Germanic rule and tried to apply it to Old Irish. It is evident from Stokes’ discussion that he had no clear understanding of the phonetic reality of Old Irish double consonant spellings such as *cc*, and many of his explanations no longer stand up to a modern understanding of Celtic historical phonology. From an early date, the notion of the operation in Celtic of a sound change comparable to Kluge’s Law was met with scepticism ( *e.g.*, GOI ( *Grammar of Old Irish* = Thurneysen, 1946) 92–93; Sjoestedt, 1926: 19–20; Martinet, 1952: 197–198).

Zupitza (1900) meant progress over Stokes’ attempts insofar as he also recognised other sources for Irish geminates. Building on a very thorough study of spellings in the major Old and Middle Irish sources, he rightly identified many instances of geminates as the products of assimilation across morphemic boundaries, but he also saw a major source of geminate stops in clusters of obstruents + * *n*. Like Stokes, many of his etymologies have been rejected in the meantime. In a similar vein, Pedersen (VKG ( *Vergleichende keltische Grammatik*) i 158–161, 476–477) distinguishes between double consonants arising from assmilation in word formation, while also being inclined to operate with a Celtic version of Kluge’s Law. In addition, he recognises doubling of consonants in terms of endearment.

Sommerfelt (1954) considered substratal influence responsible for establishing the contrast between short and long consonants in Irish, as well as that between non-palatalised and palatalised consonants. Kuryłowicz (1957) adopted a very different strategy in order to explain those instances of gemination in Celtic and Germanic that are not caused by assimilation. Critical of the notion of Kluge’s Law, he saw a different, single principle behind the phenomenon. Starting from a core where pairs of simple *vs*. geminate consonants were the product of regular sound change ( *l* : *ll*, *n* : *nn*), Kuryłowicz thinks that this opposition was extended as a morphological marker to other sounds as well.

New life was breathed into the hypothesis of the geminating force of * *n* by Lühr, 1985, who adduced further instances of geminate occlusives as evidence for that rule. She had to explain the numerous counterexamples through analogy ( Lühr, 1985: 345). In addition, Lühr’s hypothesis allows for geminates that are due to expressivity. The idea of a Celtic version of Kluge’s Law was also advocated by Bammesberger, 1998. Like a century before, most scholars remained sceptical to the usefulness of such a law in Celtic.

De Bernardo Stempel ( 1999: 508–522) provides a useful list of Old Irish words with gemination. She distinguishes between inherited geminates, *i.e.*, those geminates that arose through regular sound change, and other cases, which she largely ascribes to expressiveness. Her discussion also includes a detailed refutation of Lühr (1985). In a 2010 article, De Bernardo Stempel turns her attention to geminates in the ancient Continental Celtic languages, in particular to the possibility of gemination resulting from accentuation. Like in the case of Old Irish, she distinguishes between etymological and non-etymological gemination. This useful dichotomy between etymological and non-etymological gemination will inform the present article, too. McCone ( 2005: 406–407) touches briefly on the subject of geminates in the context of substratal influence on Insular Celtic. His remarks are specifically directed at word-initial geminates and their role in the emergence of initial mutations. He maintains that, except perhaps for *rr*, *ll*, *nn*, the status of geminated initial consonants in the historically attested Insular Celtic languages is dubious, and he downplays their significance as an indicator of substrate influence.

The status of gemination in the phonology and phonetics of the Insular Celtic languages and the part it played, or did not play, in establishing the system of initial mutations, especially in British Celtic, was the topic of a heated debate that extended for more than five decades, from the middle of the 20 ^th^ until the early 21 ^st^ century (see the more detailed summary in Van Sluis, 2019: 15–24). The debate grew out of an exchange between Jackson ( 1953: 473–480, 545–548, 565–573, 634–638; 1960; 1967: 307–308, 317–323) and Greene ( 1956; 1966); cf. also Kortlandt (1982), Feuth (1983), Koch (1989). A satisfactory solution to the problem was proposed by Harvey (1984) and Russell (1985) that removed the notion of gemination as a genuine mutation in the history of Irish and British. Afterwards, the debate shifted away from gemination towards the related question of the relationship between the spirant mutation and nasalisation in the British Celtic languages, with notable contributions by Thomas (1990), Sims-Williams ( 1990, 2008), McCone ( 1996; 92–96), Schrijver (1999), and Isaac ( 2004; 2008).

Although it can be seen from this brief history of research that many scholars have contributed to the study of Celtic gemination or to aspects of it in the past, a comprehensive modern account of the phenomenon in a diachronic perspective is lacking. The following study aims at filling this gap, by conducting a systematic analysis of all the phonological sources and internal and external contexts in which geminate sounds arose in the early history of the Celtic languages.

## 1. Proto-Indo-European and Pre-Celtic

Celtic geminate stops cannot have been inherited from Proto-Indo-European (PIE), since the Indo-European protolanguage famously did not have geminate consonants as a phonological class. This prohibition went so far that even accidental geminates across the word boundary were prone to simplification. It is believed that the so-called ‘mobile *s*’ of Indo-European arose in that way ( Mayrhofer, 1986: 120–121). However, this is not the whole story, because geminate sounds were not entirely absent from some registers. A handful of strongly affective words with geminates can be projected back to the protolanguage. Celtic languages inherited some of these words, again for use in strongly affective contexts.

(1) PIE * *atta* ‘daddy (or some other older, male relative)’ probably underlies numerous Gaulish names such as *Atta*, *Attū*, *Attios etc*. How OIr. *aite* ‘fosterfather’ belongs here, is unclear. ModIr. *oide* ‘fosterfather, tutor’ shows that this word had a /d/, not a /t/, as would be expected from PC * *tt*.

(2) PIE * *kakko-* ‘shit, excrement’ > MIr. *cacc*, W *cach*, Corn. *caugh*, MBret. *cauch*, NBret. *kac’h*. It is rather unlikely that various Gaulish names beginning with *Cac-* and *Cacc-* contain this etymon.

(3) PIE * *mamma* ‘mum’ can underlie W *mam*, MBret. *mam*, OCorn. *mam* ‘mother’, and perhaps OIr. *muimme* ‘fostermother, nurse’ and Gaulish names such as *Mammios*.

After some far-reaching simplifications of the phoneme inventory, especially the merger of the Indo-European velar and palatal series of stops and the merger of the mediae and mediae aspiratae ( Schrijver, 2016: 497–499; Stifter, 2017: 1189–1190) and the loss of laryngeals, the Pre-Celtic system of consonants was reduced to a relatively simple 15-phoneme inventory that probably did not yet distinguish geminates as a phonological category. All sound changes discussed in the following chapters operate on the basis of the post-PIE, pre-Proto-Celtic sound inventory in Table 1.

**Table 1.** the pre-Proto-Celtic sound system.

	plosive	nasal	fricative	glide	liquid
bilabial	b	m	p	w	
dental	t d	n			l r
alveolar			s (z)		
palatal				j	
velar	k g				
labiovelar	kʷ gʷ				

Marginal phonemes, *i.e.*, phonemes that occur only in very restricted contexts and which are allophones of core sounds of the system, are put in brackets here and in later sections. In keeping with the traditional practice in comparative Indo-European and Celtic studies, the phonemes /j/ and /w/ will be represented by the symbols *i̯* and *u̯* in the main part of this article.

From their virtual lack in the ancestral stages of the language, it follows that geminates must have been acquired as a phonological class after the break-up of the Proto-Indo-European parent language. It will become clear from this study that the emergence of geminates occurred gradually. There are around twenty regular, phonological sources for geminate consonants of Proto- and Common Celtic origin, plus one that is exclusive to Irish. In addition, a number of non-phonological sources (sound symbolism, loans) will be identified.

## 2. Etymological gemination: Proto-Celtic and Common Celtic

The phonetic class among which geminates started to arise earliest in Celtic are resonants. The starting point are several inherited consonantal clusters of common occurrence. They provided the input for assimilatory processes that created long, *i.e.*, geminate resonants either already in Proto-Celtic or so early after the split into individual branches that the changes spread across the entire speech community, leading to identical outcomes everywhere.

### 2.1. *ln > *ll

The assimilation of * *ln* > * *ll* is carried through in all attested Celtic languages and can therefore be securely assigned to Proto-Celtic, bolstered by a large number of convincing etymologies. The input for this change is predominantly morphological, *i.e.*, nominal or verbal derivatives where suffixes with *n-* attached to roots ending in *-l*.

(1) qPIE * *h₁elneh₂-* > * *ellā-* > OIr. *ell* ‘herd, flock’.

(2) qPIE * *h₂ebl̥neh₂-* > * *aballā* > Gaul. *aballo-*, *auallo-*, OIr. *aball*, W *afall(en)*, OBret. *aballenn*, Bret. *avalenn* ‘apple-tree’.

(3) PIE * *h₂elno-* or * *alno-* (?) > PC * *allo-* > Cib. *Allus*, *All[on]is* (?), Gaul., W *all-* ‘other, different’, prps. W *arall*, OIr. neut. *alall* ‘other’ (cf. Dunkel, 2014: 19 fn. 7, 22 fn. 9).

(4) PIE * *bʰl̥no-* ‘inflated thing’ > PC * *ballo-* > Gaul. *ballo-*, OIr. *ball* ‘member’.

(5) The reconstruction PIE * *dʰu̯l̥no-* > PC * *du̯allo-* > Gaul. *dallo-*, OIr., W, Corn., Bret. *dall* ‘blind’ is somewhat doubtful since PC * *du̯-* would be expected to be retained in Gaulish.

(6) qPIE * *k ^u̯^elno-* ‘distant’ > PC * *k ^u̯^ello-* > W, Corn., Bret. *pell* ‘far’. The reconstruction * *k ^u̯^elso-* is also possible.

(7) qPIE * *ml̥no-* ‘hesitant’ (?) > * *mallo-* > Gaul. *mallo-*, OIr. *mall* ‘slow’, W *mall* ‘evil, rotten, bad’. The reconstruction * *ml̥so-* is also possible.

(8) qPIE * *polno-* ‘full’ > * *φolno-* or * *(h₂ ^?^)olno-* > * *ollo-* > Gaul. *ollon* ‘big?; whole?’, OIr. *oll* ‘great, ample’, W *oll*, *holl*, Corn., Bret. *oll* ‘all’ (cf. Dunkel, 2014: 19, 593).

(9) qPIE * *sh₂lneh₂-* ‘salted (meat)’ > * *sallā-* > OIr. *sall* ‘bacon’. Alternatively, the reconstruction * *sh₂ldeh₂-* is also conceivable (see 3.8. (3)). The Old Irish verb *saillid* ‘to salt’, on the other hand, is synchronically derived from the noun *salann* ‘salt’, namely * *salei̯n-ī-* > * *salʲ ^†^nʲī-* > * *sailnid* > *saillid*. Its geminate is due to the later, Old Irish assimilation of *ln* > *ll*.

The change * *ln* > * *ll* is also witnessed by a group of Insular Celtic verbs that are based on old nasal-infix formations (cf. McCone, 1991: 11–23). They usually lack parallels in the Continental Celtic languages, but because of their archaic morphology they can nevertheless be securely projected back to Proto-Indo-European or at least to Proto-Celtic:

(10) PIE * *dʰh₂l̥nh₁-* or * *dʰalnh₁-* ‘to emanate, sprout’ > * *daln-* → * *dalli̯e-* > MW *deilliaw* ‘to emanate, come out’ ( Schumacher, 2004: 257–259).

(11) PIE * *gl̥-ne-H-* ‘to get power’ > * *galnV-* > * *gallV-* > W *gallu*, Corn. *gallos*, Bret. *gallout* ‘to be able, have power’.

(12) PIE * *g ^u̯^l̥neh₁-* ‘to throw’ > * *balni-* > * *balli-* > OIr. *at·baill* ‘to die’.

(13) PIE * *k ^u̯^l̥-ne-h₁-* ‘to turn’ >> * *k ^u̯^e/alnV-* ‘to go in a circle’ > * *k ^u̯^e/allV-* > OIr. *·cella* ‘to turn, go in a circle’, W *pallu* ‘to fail, weaken, cease, refuse, deny’.

(14) PIE * *pl̥-ne-h₂-* ‘to approach’ >> * *φe/alnā-* > * *φe/allā-* > OIr. *ad·ella* ‘to visit’.

(15) PIE * *u̯elnH-*/* *u̯l̥nH-* ‘to be powerful’ > * *u̯ella-*/ *u̯alla-* > OIr. *follaithir* ‘to rule’ and *follnaithir* with re-insertion of the nasal. The verbal root * *u̯aln-* can also be assumed indirectly for Gaulish, since it underlies the widely attested nominal formation * *u̯ellamno-*/* *u̯allamno-* ‘ruler’ found in the onomastic element Gaul. *uallaunos*, OIr. *Folloman*, W *Cad-wallon*.

Other possible examples are uncertain.

(16) Delamarre ( 2003: 99; but cf. IEW 524) suggests that Gaul. *callio-* ‘hoof’ and W *caill*, Bret. *kell* ‘testicle’ continue * *kalno-*, the Indo-European preform of which, perhaps * *kHl-no-*, is uncertain.

(17) OIr. *cellach* ‘strife, contention’ and *Sucellus*, the Gaulish name of a hammer-wielding god, perhaps ‘good-stiker’, can be combined under the reconstruction * *kel-n-(h₂)o-*, a nasal-infix formation from the root * *kelh₂-* ‘to strike’. However, it is far from certain that they belong together, and alternative individual explanations exist ( Matasović, 2009: 199; Zair, 2012: 183, 204).

(18) If OIr. *fillid* ‘to return’ continues the Indo-European root * *u̯el-* ‘to turn, roll’, the starting point is probably a nasal extension * *u̯el-n-*.

Finally, two important morphological processes in ancient Celtic languages must be discussed separately. One of them may have arisen, and the other certainly arose as a consequence of the assimilation * *ln* > *ll*.

In Celtiberian, this sound change resulted in a productive inflectional and derivational pattern ( Jordán Cólera, 2019: 611–614). The Celtic suffix *-on-*, particularly common with an individualising function among personal names, inflected originally with full paradigmatic ablaut, *i.e.*, lengthened grade * *-ū* < * *-ō(n)* in the nominative and full grade * *-on-* and zero grade * *-n-* in other cases. In the majority of cases, ablaut was eradicated in Celtiberian by generalising the lengthened grade across the oblique cases as well. However, the original state of affairs is still preserved in words with roots or presuffixal stems ending in *l*. The Celtiberian script does not write geminate consonants, but Motta (1981) convincingly argued that written nom. *abulu*, gen. *abulos* or nom. *statulu*, gen. *statulos* hide phonological /abulū abullos/ and /statulū statullos/. The nominative continues PC ° *lū* < PIE *° *lō(n)*, while the genitive is the regular outcome of the zero-grade in the oblique stem, namely ° *lnos*. Other Celtiberian words may also hide original * *ln* behind a written single *l*. This has, for instance, been proposed for *kelaunikui*, which Jordán Cólera ( 2019: 690) explains as * *kelnH-mno-*.

On a more speculative note, the same process can be invoked to explain the emergence of the common hypocoristic suffix * *-llo-* for names in the ancient Celtic languages. I illustrate my ideas with Gaulish, but the basic steps are valid generally. It is well-known that the morphology of shortened names often does not observe meaningful morpheme boundaries ( Schmitt, 1995: 424), but that dithematic compound names can be truncated in the middle of the second element, irrespective of the meaning and the transparency of the formation. Stüber *et al.* ( 2009: 256) cites *Adnema* as a shortened form of a compound with * *nemes-* ‘sky’ or * *nemeto-* ‘sanctuary’ as second element, and *Verca* as a shortening of a compound of * *u̯er-* ‘on, upon’ and a second element starting with * *k-*. At the same time, shortened names often appear as *on*-stems (see Stüber, 2004 for that suffix in general). For example, the compound name *Boudilatis* (perhaps ‘having the fury/heat of booty/victory’) consists of the two lexemes * *bou̯di-* ‘victory, booty’ and * *lāti-* ‘warrior fury’. This could be truncated to the short name * *bou̯dilo-* (not attested), which could in turn be ‘individualised’ as * *bou̯dil-on-*. The suffix, which originally was fully ablauting, would have led to a paradigm nom. sg. * *bou̯dilū*, acc. sg. * *bou̯dilonam* (perhaps attested as *Bodilo*) with full and lengthened grades in the strong cases, and an oblique stem * *bou̯dill-* < * *bou̯dilØn*- with zero-grade suffix and regular assimilation. The oblique stem then provided the springboard for newly thematised * *bou̯dillo-* (attested as *Boudillus*), whence * *-llo-* could be reanalysed as a suffix in its own right added to the *i*-stem * *bou̯di-*. The notional connection with * *bou̯dilāti-*, or a compound with any other second element starting in * *l-*, had been lost at that stage. The derivation was felt to operate directly on the first element alone, which could be extended to nouns other than *i*-stems. An alternative, slightly variant explanation of the suffix * *-llo-* starts from monothematic, originally adjectival names in * *-lo-*, which were likewise ‘individualised’ by the addition of * *-on-*, *e.g.*, * *kamulo-* ‘servant’ or ‘champion’ → ‘individualised’ * *kamulon-*, * *kamuln-* → * *kamullo-*, attested as *Camullus*.

In a further step, the presence of gemination in a suffix with *-l-* provided the starting point for the analogical introduction of consonantal gemination into other suffixes, in particular the very common suffixes in * *-ko-*, to which doublets in * *-kko-* were then created. This explains the observation (section 9.2.4.) that some geminate sounds show a notable propensity to occur in suffixes.

### 2.2. *sm > *mm

The Proto-Celtic language showed a tendency towards weakening of word-internal *s* ( Stifter, 2012: 541–542; Stifter, 2017: 1192), so much so that in various word-internal clusters with resonants, it was prone to disappear through assimilation to the resonant or, in other words, with compensatory lengthening of the resonant. However, only one such development can lay a secure claim on Proto-Celtic age by virtue of being attested for Celtiberian, namely * *sm* > * *mm*.

(1) The PIE pronominal dative singulars * *Hi̯osmōi̯* ‘to whom’ and * *tosmōi̯* ‘to him’ show up, *via* PC * *i̯ommūi̯* and * *sommūi̯* (with generalisation of the stem allomorph * *so-*), as *iomui* and *somui* in Celtiberian.

(2) PIE 1sg. * *h₁esmi* ‘I am’ > * *emmi* > Gaul. *immi*, OIr. *am*.

It is interesting to note that while Celtic * *s* is weak and feeble in sonorant contexts, it is strong and ‘aggressive’ in contexts with stops. That is to say, especially when following an obstruent, that sound is ‘weakened’ and eventually completely assimilated to the * *s*. However, as soon as *m* is involved as well, it is the one that finally prevails. In less flowery language, * *Tsm* (where *T* is any obstruent) becomes * *mm* already in Proto-Celtic. Neuter verbal abstracts in * *-s-man-* are one class of words where this can be observed. One example illustrates the pan-Celtic treatment of such complex clusters:

(3) qPIE * *g̑ʰn̥gʰ-s-men-* ‘act of stepping, striding’ → pre-Celtic (pre-Clt.) * *kangsman-* > * *kanχsman-* > * *kãmman-* > OIr. *céimm*, W, Corn. *cam* ‘stepping, step’; cf. Bret. *kammenn* ‘detour’; thematic derivatives are attested in Cib. *kamanom* ‘path’ and Gaul. * *cammīnos*, which survives as a loanword in Ital. *cammino*, Fr. *chemin*, *etc*. ‘path, way’ ( Hamp, 1974).

Another example is supported by three languages:

(4) PC * *garsman* ‘shout, cry’ > * *garmman* > OIr. *gairm*, W, Corn., Bret. *garm*, and perhaps Gaul. *garma[n]*. For this explanation, I assume that * *s* in the cluster * *rsm* assimilated to the * *m*, and that the resulting * *rmm* contrasted with more common * *rm* to such an extent that it did not undergo the spirantisation to * *rṽ* in British. If, however, the * *s* assimilated to the preceding * *r* rather than to the following sound and the resulting * *rrm* merged with * *rm* before British spirantisation, unspirantised Brit. *garm* may be a borrowing from Irish (personal communication Paulus van Sluis).

Numerous other examples of neuter verbal abstracts of this type are conveniently collected in Stüber ( 1998: 45–83; 2015: 114–115) and need not be repeated here.

(5) A special case is PC *amman-* ‘time’, attested in Gaul. *amman*, OIr. *amm*, and in the derivatives OIr. *aimser*, W *amser*, Bret. *amzer* ‘time, weather’. It can either continue PIE * *h₂et-s-men-* or maybe ‘asigmatic’ * *h₂et-men-* ‘the act of going around’ ( Stifter, 2017b).

(6) Structurally similar are masculine and feminine agent nouns with the suffix * *-s-mon-*, *e.g.*, OIr. *rúam* ‘red dye’ < * *rou̯dsmon-* < qPIE * *h₁reu̯dʰ-s-mon-*, or OI. *femm*, *femmain*, W *gwymon*, Corn. *gumman*, Bret. *goumon*, Fr. *goémon* ‘edible sea-weed’ < * *u̯immon*-, perhaps from qPIE * *u̯ip-s-mon-* ‘swayer’ ( Stifter, 1998: 204–207).

### 2.3. *ndn, nnd (and *nkn) > *nn

Triple clusters of two * *n* and a single * *d*, either in the order * *ndn* or * *nnd*, appear to have been simplified to a geminate * *nn* already in Proto-Celtic. Two or perhaps three nouns fall in the first group:

(1) qPIE * *bn̥d-no/eh₂-* > * *bandno/ā-* > Gaul. *banno-*, *benno-*, OIr. *benn*, W *ban*, Bret. *bann* ‘top, tip, summit’. The Germanic cognates, OEngl. *pintel* ‘penis’ or MHG *pinz* ‘awl’, point to a preform with * *b-*, so the word is perhaps a substratal loanword.

(2) qPIE * *gln̥d-neh₂-* > * *glandnā-* > * *glannā-* > Gaul., OBrit. *glanna*, W, Corn. *glan*, Bret. *glann* ‘river-bank’. OIr. *glenn* ‘valley’ is a hybrid of this * *glandnā-* and * *glendes-* (W *glyn*, Bret. *glenn* ‘glen, valley’). The cognate MLG *klint* ‘hill’ suggests a preform with two unaspirated mediae, which makes the item suspect of a substratal loan. A connection with the verbal root * *gʰlendʰ-* ‘to look, shine’ is semantically not attractive.

(3) qPIE * *g⁽ʰ⁾rn̥d⁽ʰ⁾no-* > * *grandno-* > Gaul. *grannus*, OIr. *grenn* ‘beard, bristles’, W. *gran* ‘cheek, jaw, beard’, Bret. *gour(r)enn* ‘eye-lash’; Provençal *gren* ‘moustache’ and OFr. *grenon* ‘beard’ are perhaps borrowed from Gaulish. The vocalism across the Celtic languages, which is parallel to that of the preceding two items, suggests a structure similar to those. IEW 440 thinks of a connection with the root * *gʰer-* or * *gʰreh₁-* (in modern notation) meaning ‘to sprout, stick out’.

(4) Van Sluis *et al*. (2023) propose that qPIE * *kentno-*, a thematic derivative of the *n*-stem * *kenton-* ‘membrane, skin’, was simplified to * *kenno-* > OIr. *cenn*, W *cen*, Corn. *cen*, OBret. *cennen* ‘skin’.

(5) The reconstruction PC * *tundnā-* is a formal possibility for OIr. *tonn*, W, LCorn. *ton*, Bret. *tonn* ‘wave’. This could be analysed as a formation from the PIE root * *(s)teu̯d-* ‘to push’ with a nasal infix taken over from the verbal inflection, *i.e.*, structurally qPIE * *tu-n-d-neh₂-* ( Stifter, 2023a: 179; other possible cases of nasal infixes transferred from verbal stems into the nominal inflection in Celtic are discussed in Stifter, 2018: 38). See 3.1. (10) for an alternative reconstruction of *tonn*.

Clusters with two nasals are particularly common in the present stem formation of verbs.

(6) OIr. *ro·finnadar* ‘to find out’ ultimately continues the PIE root * *u̯ei̯d-* ‘to see’. First, the *n* of the inherited nasal infix formation * *u̯i-n(e)-d-* became fossilised as * *u̯ind-*, and then another nasal suffix was added to this neo-root, *i.e.*, PC * *u̯ind-nu-*, which directly underlies the Old Irish present stem.

(7) Structurally similar, although involving a guttural instead of a dental, is OIr. *srennaid* ‘to snore’, probably a denominal verb from the noun *srenn* ‘snoring’ < * *srenk-no-* or * *srengʰ-no-* (the precise character of the guttural is difficult to determine).

McCone (1998) sets up several Celtic verbal stem formations as ‘double nasal presents’ with the sequence * *nnd*. The envisaged development is illustrated by the Indo-European root * *gʰed-* ‘to grab’. This had a nasal present in Indo-European, from which the secondary root * *gʰend-*/ *gʰn̥d-* was extrapolated in Celtic. This then received a new nasal infix, *i.e.*, * *gan-n-d-e/o-* ‘to have room’, reflected by OIr. *ro·geinn*, W *genni* ( Schumacher, 2004: 330). Most of the pertinent roots already contained a fixed *n* in Proto-Indo-European. Schumacher (2004) includes the following verbs with this structure in his collection of Celtic primary verbs: * *bran-n-d-e/o-* ‘to well out’ < * *bʰrend⁽ʰ⁾-* (233–234) * *gan-n-de/o-* ‘to find room’ < * *gʰend-* ←* *gʰed-* (330–331); * *glan-n-d-e/o-* ‘to bring to light’ < * *gʰlendʰ-* (334–337); * *skan-n-d-e/o-* ‘to jump’ < * *skend-* (574–575); * *tan-n-d-e/o-* (or * *tend-e/o-*) ‘to break, cut’ < * *tend-* (614–615). In the case of * *gri-n-d-e/o-* ‘to drive, impel’ < * *gʰrei̯dʰ-*, the change to OIr. *·greinn* is possibly analogical and may have occurred only during the historical period ( McCone, 1998: 473; Schumacher, 2004: 354).

In addition to these relatively common sources for the geminate resonants *ll*, *mm* and *nn* mentioned in the foregoing, a number of rarer consonant clusters may have fed into the creation of marginal geminate obstruents through regular phonological change.

### 2.4. *dk > *kk

A handful of Insular Celtic examples illustrate the assimilation of * *dk* > * *kk* at the end of root syllables. Since this assimilation is also found across the composition boundary in Gaulish (see section 6.2.), it is likely that the change is already Proto-Celtic.

(1) qPC * *ad-kii̯o/ā-* ‘nearness (< *at-ness)’ > OIr. *aicce* ‘proximity’, W *ach* ‘beside, near’.

(2) qPIE * *h₁rud-ko-* ‘red’ → * *rud-kii̯o-* ‘redness’ > * *rukkii̯o-* > OIr. *ruccae* ‘shame’.

(3) qPIE * *pr̥d-keh₂-* > * *φridkā-* > * *φrikkā-* > W *rhech* ‘fart’.

(4) qPIE * *slHd-kV-* ‘slay-thing’ > * *slad-kV-* > * *slakkV-* > OIr. hapax *slacc* ‘sword’, ModIr. *slacán* ‘bat’ ( Schumacher, 2004: 585).

*A fortiori* one might expect that * *kk* would also continue * *gk* and * *tk*, but examples are difficult to identify.

(5) In the case of inherited PIE * *h₂r̥tk̑o-* ‘bear’, the outcome is Gaul. *arto-*, OIr. *art*, W *arth* < PC * *arto-* < * *arχto-* < * *artko-* with metathesis of the dental and the guttural at the syllable onset, as must also have happened in * *dʰg̑ʰomi̯o-* ‘earthling, human’ > * *dgoni̯o-* > * *gdoni̯o-* > Gaul. *-χtonion*, OIr. *duine*, W *dyn*, Corn., Bret. *den* ‘person’.

### 2.5. ‘Geminating’ *p?

It is possible that the weakening and loss of IE * *p* after resonants led to a marginal increase of geminates. The best examples involve the cluster * *rp*:

(1) PIE * *sterp-* or * *stirp-* → * *sti/erφāko-* > OIr. *serrach* ‘colt, foal; young animal’. For the root, cf. Lat. *stirps* ‘stem, stump, stock, race’, and Lith. *stir̃pti* ‘to grow up’ and *ster̃ptis* ‘to stiffen’.

(2) PIE * *serpeh₂-* ‘sickle’ > * *serφā-* > * *serrā-* > OIr. *serr*, MW, MCorn. *serr*, OBret. *ser* ‘sickle’. A loan from Lat. *serra* ‘saw’ < * *sersah₂-* is not excluded, however, especially in view of the retention of initial *s-* in British instead of its regular development to *h-*. Finally, the Celtic word could even be cognate with the Latin.

The cases of * *lp* are more problematic:

(3) The root PIE * *telp-* ‘to make room’ is probably continued by OIr. *do·alla*, *·talla*, occasionally *do·ella* ‘there is room for; to find room’. At first glance, this verb seems to support the notion of the change * *lp* > * *ll*, but perhaps the * *ll* is rather due to a nasal-infix formation, *i.e.*, * *tl̥-n-p-* > * *taln-(φ)-* > * *tall-*. The deuterotonic form *do·alla* must be analogical.

(4) Pedersen (VKG i 78–79) compares OIr. *sell* ‘iris’, *sellaid* ‘to see, perceive’, MW *syllu* ‘to gaze’, Bret. *sellout* ‘to watch’ with Gr. στιλπνός ‘brilliant’, στίλβω ‘to shine, be brilliant’. Even if that comparison were correct, the geminate *ll* could be due to the * *n* of the suffix, not the * *p*, *i.e.*, * *stilpno-* > * *stilφno-* > * *stilno-* > * *stillo-*. For an alternative explanation of PC * *s(t)ill-* see section 3.7.

(5) pre-Celtic * *kelpurno-* > OIr. *cilorn*, W *celwrn*, OBret. *chilorn*, Bret. *kelorn* ‘vessel’, perhaps also in the Old British placename *Cilurnum*, speaks clearly against the notion of such a sound change. The word, which has cognates in Lat. *calpar* and Gr. κάλπις ‘vessel, pitcher’, is possibly a substratal loan (cf. Van Sluis forthc.).

(6) OIr. *col*, W *cwl*, OBret. *col* ‘wrong, sin’ could be related to Lat. *culpa* ‘blame, guilt’, in which case they would continue PE * *kulφo-* < * *kulpo-*, but alternative explanations are possible.

Finally, it is conceivable that instances of the very rare inherited cluster * *pk* led to geminate * *kk*. Before a voiceless dental, PIE * *p* became PC * *χ via* the intermediate stage * *φ*, *e.g.*, * *septm̥* ‘7’ > * *seφtam* > PC * *seχtam*, cf. OIr. *secht ^N^*, MW, LCorn., OBret. *seith*, Bret. *seizh*. This means that it fell together with any kind of tectal sound in this position. It is conceivable that, in a parallel manner, * *pk* became * *χk via* * *φk*, which was then further assimilated to * *kk* ( Testen, 1999). Possible examples are, by necessity, few, but this explanation could account for the apparent suffix in some words for ‘swine’, *e.g.*, * *su(H)-pk̑-u-* ‘swine-stock’ > * *sukku-* → W *hwch*, Corn. *hoch*, Bret. *houc’h* ‘pig’, OIr. *socc* ‘ploughshare, snout’, or * *mo(H)-pk̑-u-* ‘big (?) stock’ > * *mokku-* > Gaul. *Moccus*, OIr. *mucc*, W *moch*, Corn. *mogh*, Bret. *moc’h* ‘pig’. The suffixal element is the zero-grade of PIE * *pek̑u-* ‘small livestock’ ( Testen, 1999). Perhaps this suffix is also contained in * *brokkV-* ‘badger’ > Gaul. *brocco-*, OIr. *brocc*, W, Corn. *broch*, Bret. *broc’h*, although the word is more likely a substratal loan (see 13.1. (5)).

## 3. Etymological gemination: developments across branches with identical or similar outcome

Further assimilations, predominantly involving clusters with resonants, took place in a staggered fashion after the end of the Proto-Celtic linguistic unity. From what the fragmentary documentation of the ancient Celtic languages allows us to see, none of these changes were carried through in all Celtic languages. Gaulish and, more importantly, Celtiberian sometimes reflect a more conservative stage. In the Insular Celtic languages, however, the changes discussed in this chapter are completely carried through. This is not a proof for a genetically close relationship between Goidelic and British, but it may be a function of their later attestation, *i.e.*, tendencies towards assimilation, which had their kernel much earlier, had enough time to unfold completely in these languages.

What unites these assimilations and those of the previous section, is that they occur word-internally, but not word-initially, and that they happened after the vocalisation of syllabic resonants. There is no single overarching rule that accounts for all contexts.

### 3.1. *sn > *nn

Two forms in Celtiberian appear to indicate that, unlike * *sm*, * *sn* had not assimilated to * *nn* already in Proto-Celtic, even though there is ample evidence from the three other branches that the change was carried through before the historical period. The two items that show the conservative behaviour of Celtiberian are:

(1) PIE * *pr̥h₃-sneh₂-* > * *φrasnā* > * *φrannā* > OIr. *rann*, W *rhan*, MCorn. *ran*, Bret. *rann* ‘part, share’. If Cib. *arznas* in Botorrita I derives from the same preform ( Jordán Cólera, 2019: 107, 121), it would demonstrate that the assimilation to * *nn* was not of Proto-Celtic age, but the divergent syllabification of the initial resonant is to be noted.

(2) The comparison of OIr. *trén*, ogam TRENA- ‘strong’, which continues PC * *treχsno-*, with Gaul. *Trenos* indicates that clusters of the type *- *Csn-* were subject to an early reduction to simple * *-n-* at least at the Core Celtic stage. Cib. *masnai*, also in Botorrita I, has been suggested to continue either * *mak-snā-* ‘enclosure’ oder * *mad-snā-* ‘breaking’ (see Wodtko, 2000: 244). In either case, the treatment of the triple consonant cluster deviates from that in * *treχsno * and the cluster *-sn-* is evidently maintained.

Strong evidence for the change * *sn* > * *nn* in the languages apart from Celtiberian comes from the following items:

(3) The Old Irish paradigm of nominative (nom.) *brú* ‘bosom’ < PC * *brusū*, oblique stem *bronn-* < * *brunn-* < * *brusn-* is a morphological fossile that proves that the suffixal ablaut of PIE * *bʰrusō(n)*, * *bʰrusn-* had been inherited into Proto-Celtic. The weak stem * *bʰrusn-* also underlies * *brunni̯o*-, *i.e.*, OIr. *bruinne* ‘bosom, breast’, W *bryn* ‘hill, mound, prominence’, Corn. *bren* ‘hill’, and was also borrowed into Proto-Germanic * *brunjōn-* ‘breastplate’.

(4) qPIE * *dʰusno*- ‘smoky’ > * *dunno-*, Gaul. *dunno-*, OIr. *donn* ‘brown’, W *dwn* ‘some shade of brown’.

(5) PIE * *h₁osno-* > * *onno-* > Gaul. *onno-*, OIr. *onn* and, with further suffixation, *uinnius*, W *onn*, OCorn. *onnen*, Bret. *ounn* ‘ash-tree’.

(6) PIE * *h₁u̯h₂sno-* ‘empty’ > * *u̯anno-* > OIr. *fann*, W *gwan*, OBret. pl. *guenion*, Bret. *gwan*, OCorn. *guan* ‘weak, feeble’. For the problems with the PIE reconstruction, see Zair ( 2012: 46–47).

(7) qPIE * *k _ə_sn-* or * *kasn-* → * *kannīnā-* > OIr. *cainnenn*, W *cennin*, OCorn. *kenin*, Bret. *kinnen* ‘onion, leek’. This etymon is perhaps also contained in the ancient Germanic ethnonym *Canninefates*, if it means ‘leek lords’.

(8) qPIE * *k̑ _ə_snih₂-* or * *k̑asnih₂-* ‘female grey one’ > * *kasnī-* > * *kannī-* → W *ceinach* ‘hare’. This etymon may also underly the OIr. personal name *Cainnech*, possibly from * *kannīko-*; this name is unlikely to be derived from *cainnenn* ‘onion, leek’, since that word did not syncopate when an extra syllable was added, as the genitive *cainninne* shows.

(9) pre-Celtic * *k ^u̯^resno-* (perhaps a substratal loanword) > * *k ^u̯^renno-* > LGaul. *prenne*, OIr. *crann*, W, Corn. *pren*, Bret. *prenn* ‘tree, wood’; the vocalism of OIr. *crann* may be due to some sort of ablaut. For the underlying root * *k ^u̯^res-*, cf. * *k ^u̯^resti̯o-* > W *prys*, Sc. Gael. *preas* ‘copse, grove’, and * *k ^u̯^erstV-* > OIr. *cert* ‘name of a letter’, W *perth*, Corn. *-perth* ‘hedge, bush’. It has been suggested that the same etymon be contained in OIr. *currach*, *cuirrech* ‘swamp, bog’ ( *Léxique Étymologique de l’Irlandais Ancien* (LEIA) C-278; see 3.2. (5)).

(10) qPIE * *tuh₂sneh₂-* ‘swelling (?)’ > * *tusnā-* > * *tunnā-* > OIr. *tonn*, W, LCorn. *ton*, Bret. *tonn* ‘wave’ ( Stifter, 2023a: 179; Zair, 2012: 155). For an alternative explanation, see 2.3. (5).

(11) qPIE * *u̯l̥Hsno-* ‘wounded (?)’ > * *u̯lanno-* > OIr. *flann* ‘(blood-)red’ ( Zair, 2012: 73).

(12) I have suggested a pertinent etymology for Gaul. * *dannos* ‘title of a public or religious official’, namely *via* * *dasno-* from PIE * *dʰh₁sno-* ‘pertaining to the religious sphere’, cf. Lat. *fānum* ‘sanctuary’ ( Stifter, 2011: 166–167).

(13) Matasović ( 2009: 416; 2020: 337–338) sets up * *u̯esnālā-*, derived from PIE * *u̯esr/n-* ‘spring’, as the preform for OIr. *fannall*, W *gwennol*, Bret. *gwennel* ‘swallow’. I have instead proposed a substratal loan from a preform * *u̯annell-* ( Stifter, 2010).

### 3.2. *rs > *rr

A series of words, not always of clear etymological analysis, suggest that clusters of * *r* + * *s* were preserved in some form in Celtiberian ( Jordán Cólera, 2019: 121). Instances of *r* + sibilant are Cib. *arznas* (already discussed in 3.1. (1)), *tikerzeboz*, *uerzoniti*, *uerzaizokum*. *Uerzaizokum* has been connected with the root * *u̯ers-* ‘better, elevated’; for *uerzoniti*, an analysis as * *uper-sonh₂-ei̯e-ti* has been proposed (cf. Jordán Cólera, 2019: 202, 208). However, the explanation of none of these words is beyond doubt. Other examples such as the coin legends *arzakoz*, *arzakozon* and *arzaoz* ( Jordán Cólera, 2019: 279–348) are too unclear to be used as evidence. They could reflect Iberian names. A single Hispano-Celtic example has been adduced as positive proof for the assimilation of * *rs* > *rr*, namely the placename adjective *erredicis*, which Prósper ( 2002: 318) analyses as * *per-sed-i̯o-*. In view of the apparent retention of * *rs* otherwise in Celtiberian, this analysis must remain doubtful.

The Gaulish corpus also contains several examples with the surface sequence *-rs-*. Whatever is the precise analysis of the names Ουερσικνος, *Versenus* and *Versinius*, it can be argued that they are productively formed compounds with the intensive prefix * *u̯er-* ‘super’ + lexemes beginning with *s-*, *e.g.*, * *seno-* ‘old’. They are therefore not reliable examples for the treatment of inherited * *rs* in Gaulish. Several Gaulish names are derived from the etymologically obscure stem *darso-*. Delamarre ( 2003: 136) tentatively compares this with Late Lat. *darsus*, the name of a fish (Fr. *dard*, Engl. *darter*). Bret. *dars* ‘id.’ must surely be borrowed from Latin or Romance. The reading *incors* in the inscription from Larzac is uncertain (there is a space between the *r* and the *s*, so the two letters could belong to different words). The Cisalpine Celtic corpus has one example of *-rs-*, the patronymic *uarsileos* of unclear analysis. Finally, the names *Morsinus* and *Morsius* of unclear analysis are from Noricum and may not be Celtic at all.

Apart from cases that are recent, productively formed compounds, the precise nature of the clusters in the examples above is uncertain. Are they inherited instances of *-rs-* or did they also arise through secondary processes or through re-analysis? This question is all the more legitimate since Gaulish does provide examples that show the operation of the change * *rs* > * *rr*. In order to reconcile the contradictory evidence, one can speculate that the change was just under way in Gaulish at the beginning of the historical period.

(1) PIE * *kr̥so-* ‘runner’ > * *karso-* > * *karro-* > Lat. *carrus* (from Gaulish), OIr. *carr*, W *car*, Bret. *karr* ‘cart’.

(2) PIE * *bʰr̥so-* ‘point’ > * *barso-* > * *barro-* > Gaul. *barro-*, *baro-* (?), OIr. *barr*, W *bar*, Bret. *barr* ‘top’.

In the Insular Celtic languages, the change has been carried through completely, as further examples demonstrate:

(3) PIE * *g̑ʰerso-* ‘small, short’ > * *gerso-* > * *gerro-* > OIr. *gerr* ‘short (cropped)’, beside *gair* ‘near’ < * *g̑ʰr̥i-*.

(4) PIE * *h₁erseh₂-* ‘hinder part, buttocks’ > * *errā-* > OIr. *err*. Perhaps also in *eirr* ‘chariot fighter’, if from * *ers-sed-* ‘he who sits at the back’, in contrast to *arae* ‘charioteer’ < * *are-sed-* ‘he who sits at the front’.

(5) It has been suggested to compare OIr. *currach*, *cuirrech* ‘swamp, bog’ with OE *hyrst* ‘hillock, wooded eminence’, OHG *horst* ‘wood’ < * *k ^u̯^r̥sti-* (cf. LEIA C-278; but see also 3.1. (9)), which requires an ablauting preform * *k ^u̯^orsīko-* or * *k ^u̯^orsiko-* > * *k ^u̯^orrī̆ko-*. It is not entirely clear if the *o* in the sequence * *orri* would be raised to * *urr* in Old Irish.

(6) PIE * *u̯r̥seh₂-* ‘high point’ > * *u̯arsā-* > * *u̯arrā-* > OIr. *farr* ‘prop, post’, W *gwar* ‘nape of the neck’, OCorn. *guar* ‘neck’.

(7) PIE * *u̯erso-* ‘being on a high point’ > OIr. *ferr* ‘better’ This is the same etymon as the preceding item, but with a different ablaut grade. See also W *gwell* in 3.7. (1).

(8) qPIE * *dor-so-* > * *dorro-* > OIr. *dorr* ‘harsh, rough’, *doirr* ‘anger’. This example is uncertain, since the word is restricted to Irish and is late attested. If inherited, it may be built on the PIE root * *der-* ‘to tear’, for possible extra-Celtic cognates see LEIA (D-159) and Matasović ( 2009: 103). See also section 11.3. (2).

(9) PIE * *(s)tord-s-V-*, * *(s)tr̥d-s-V-* > * *torsV-*, * *tarsV-* > OIr. *tarr* ‘stomach’, *torrach* ‘pregnant’, OW *torr*, W, Corn. *tor*, Bret. *tor*, *teur* ‘stomach’, W *torrog* ‘pregnant’. IEW 1024 and LEIA (T-33–34) derive this etymon from the extended Indo-European root * *(s)ter-d-* ‘to be stiff, sturdy’, with reference to an inflated belly. Matasović ( 2009: 385), on the other hand, doubts the Indo-European pedigree of the word.

(10) The merger of the preposition *for* ‘over, upon’ < * *u̯or* ←* *u̯er* < * *uper* with enclitic pronouns starting with *s-* has resulted in inflected prepositions with *-rr-* in Old Irish, namely 3sg. fem. *forrae*, *fuirre* < * *u̯or-sii̯am*, and 3pl. *forru* < * *u̯or-sūs*.

### 3.3. *ls > *ll

Like in the case of the preceding change, Celtiberian provides an example that shows the unassimilated cluster, namely VELSAM. Due to the lack of a clear analysis of the word, it is uncertain whether its internal cluster is the retention of an inherited sequence or is the result of a secondary development (see Jordán Cólera, 2019: 908 for proposals, none of which is compelling). Other names in Celtiberian sources with the sequence *-ls-* such as *belsa*, *belsu*, *kelse* are probably borrowings from Iberian. There is no secure example in Celtiberian of the change * *ls* > * *ll* having taken place. As for Gaulish, Delamarre ( 2007: 194) records the name (in the dative) *Velsounae Suiocae Vescleuesis f.* from Dalmatia. Its provenance, together with the un-Celtic name of the father (* *eu̯* should have become * *ou̯* in Celtic), renders it unlikely that *Velsouna* is Gaulish. The Gaulish inscription from Larzac (L-98 2b3) contains the form *(s)uolson*, which Lambert (cited in Delamarre, 2003: 327) tentatively compared with W *gwall* ‘mistake, error’ (see below).

In the Insular Celtic languages, at any rate, the change has been carried through:

(1) PIE * *melso-*, * *ml̥so-* ‘deceit, fault’ > * *melso-*, * *malso-* > * *mello-*, * *mallo-* > OIr. *mell* ‘destruction, confusion, error’, W *mall* ‘destruction, evil’; perhaps also the first element *mallo *, *mello-* in Gaulish personal names.

(2) PIE * *pl̥so-* ‘rock’ > * *φalso-* > * *φallo-* > OIr. *all* ‘cliff’.

(3) From the Indo-European base * *u̯el-s-* ‘deceit (?)’ (IEW 1140), a full complement of ablaut formations may be reflected in Celtic. The full grade PIE * *u̯elso-* gives * *u̯ello-* > OIr. *fell* ‘deceit, treachery’. The zero grade PIE * *u̯l̥seh₂-* > * *u̯alsā-* > * *u̯allā-* can underlie OIr. *fall* ‘neglect, negligence’, W *gwall* ‘fault, error, failure’, Bret. *gwall* ‘bad’. Alternatively, W, Bret. *gwall* and Gaul. *uolson* (see above) can also continue the *o*-grade PIE * *u̯olso-*.

(4) * *k ^u̯^elso-* ‘distant’ and * *ml̥so-* ‘hesitant’ are viable alternative reconstructions for the adjectives that have been discussed as * *k ^u̯^elno-* and * *ml̥no-* in section 2.1. above.

### 3.4. *sr

The case of * *sr* is different. The alleged change * *sr* > * *rr* in OIr. *errach* ‘spring’ < * *u̯esr-* (VKG i 82) is highly questionable. Not only is there no good parallel for this change (see the arguments below), but the loss of the initial * *u̯* would also be unprecedented. *Errach* is better analysed as an adjectival derivate of *err* ‘hinder part, extremity, tail’ (see 3.2. (4)), *i.e.*, ‘that which is at the end (of the winter)’. The spelling *dírruidiguth* ‘derivation’ beside *dírṡuidigud* (both in the St Gall glosses, namely Sg. 53a11 and 188a8) for the verbal noun of *díṡruthaigidir* ‘to derive’ does not prove the Proto-Celtic assimilation of * *sr* > * *rr* since the verb is an Old Irish calque on Latin *dērīuāre*, which shows the synchronic ‘strengthening’ or devoicing of *r* caused by lenited * *ṡ*.

The best examples of inherited * *sr* show a single *r* as the outcome in Irish and, to a lesser degree, in British, but the details of the intermediate stages are not entirely clear. It is possible that * *s* became * *ð* before * *r*, perhaps passing through a stage * *z*. This is best illustrated by the feminine numeral * *tisres* > Gaul. *tidres*, OIr. *teóir*, MW *teir* ‘3’ ( Kim, 2008: 160−161). On the basis of metrically disyllabic *teüir* in the two poems on the first page of the Milan manuscript of Old Irish glosses (MS Ambros. C301 inf.; *Thes*. ii 291‒292), McCone ( 1996: 47) wants to derive this from * *tēsūres*. However, the metrical evidence is not conclusive, since the poem is comparatively late, perhaps from c. 850, and the hiatus of *teüir* can be poetical licence.

The development * *sr* > * *r* suggested here is supported by the following examples:

(1) PIE * *kēs-reh₂-* ‘tool for combing’ > * *kīsrā-* > * *kīrā-* > OIr. *cír* ‘comb’.

(2) PIE * *u̯ōs-rV-* ‘related to spring’ > * *u̯āsrV-* > * *u̯ārV-* > OIr. *fáir*, W *gwawr* ‘sunrise, east’.

(3) The case of * *mēmsro-* (from * *mēms-* ‘piece of meat’) > OIr. *mír* ‘bit, piece of meal’, although similar, is best kept apart, since a more complex consonant cluster is involved.

(4) Delamarre ( 2003: 128–129) explains the Gaulish compound element *craro-* as ‘hornet’ < PIE * *k̑r̥h₂sro-* (cf. Lat. *crābrō*), but this is pure speculation since its meaning is unknown. If the etymology is correct, it would show the loss of * *s*.

(5) PIE * *su̯esōr*, *su̯esr-* ‘sister’ > * *su̯esūr*, *su̯esr-* is continued as Gaul. dat.pl. *suiorebe*, OIr. *siür*, gen. *sethar*, nom.pl. *sethir*, W *chwaer*, OCorn. *huir*, Corn. *hôr*, Bret. *c’hoar*. Of these, the Gaulish and British forms are irrelevant here since they continue the full-grade allomorph of the stem. Only the allomorph *seth(V)r-* in Old Irish is potentially relevant for the present question. It has been suggested that *- *θr-* in the oblique cases continue directly the zero grade * *-sr-* of the suffix. However, this cannot be used as proof for the regular treatment of * *sr* in Celtic since these forms may have been remodelled after the other kinship terms, which all have oblique stems in *-thr-* from * *-tr * ( McCone, 1994: 277–278, 283).

### 3.5. *sl > *ll

Only ambiguous evidence is found for the single relevant example in Ancient Celtic.

(1) PIE * *koslo-* ‘hazel’ > * *kollo-* > OIr., W, Bret. *coll*, OCorn. *colwiden*. Delamarre ( 2003: 127) refers various Gaulish personal and placenames to this etymon: the personal name *Collus* and the placename *Collex* < * *Collācon* in Switzerland have geminate *ll*. However, the French placenames *Coole* < *Cosla* (attested 983), *Coolus* < *Coslus* (attested 869), *Coulon* < ancient *Coslumnus* display unassimilated *sl* still in historical times, as does the divine name *Cuslanus* in Cisalpine Gaul. If the latter forms belong to this etymon, the change can only have happened subsequent to the Proto-Celtic stage.

In the Insular Celtic languages, at any rate, the change has been carried through.

(2) qPIE * *dʰrus-li-* ‘fragment’ > * *drusli-* > * *drulli-* > W *dryll*, Bret. *druilh*, Corn. *dral*.

(3) qPIE * *k ^u̯^ei̯sleh₂-* ‘faculty of seeing (?)’ > * *k ^u̯^ei̯slā-* > * *k ^u̯^ei̯llā-* > OIr. *cíall*, W *pwyll*, Bret. *poell* ‘sense, reason’. The underlying root is PIE * *k ^u̯^ei̯s-* ‘to see, perceive’.

(4) qPIE * *neu̯H-slo-* or * *nou̯Hslo-* ‘shout, cry’ > * *nou̯slo-* > * *nou̯llo-* > OIr. *núall* ‘cry, noise’.

(5) qPIE * *(s)tug/k-slo-* ‘treated with a hammer or chisel (?)’ > * *tuslo-* > * *tullo-* > OIr. *toll* ‘pierced’, W *twll*, Corn. *toll*, Bret. *toull* ‘hole, perforation’. There are personal and placenames with *tull-* in Gaulish, but their relationship with this etymon are unclear.

Geminate - *ll-* is also the outcome in Old Irish verbal compounds when root-initial * *sl-* comes to stand between two vowels. For instance, the compound verb * *to-sli-i̯e/o-* ‘to earn’ comes out as deuterotonic *do·slí*, but prototonic · *tuilli*. In British, in contrast, root-initial * *sl-* in such contexts is treated like lenited *l*, *e.g.*, the root * *slad-* ‘to slay’ occurs in the compounds W *ymladd* ‘to kill, fight’, Corn. *omladh* ‘to fight’, or OBret. *anlaedam* ‘I attack’. In the case of W *dyrllid*, Bret. *dellit* ‘to earn’ < * *to-ro-sli-i̯e/o-*, *ll* is not a trace of * *sl*, but is due to the deleniting effect of *r* upon a following *l*.

### 3.6. *nl > *ll

Only examples from Irish come to mind for this assimilation. Two are compounds with the preverb * *en-* + a second element starting in * *l-*. It is not excluded that their behaviour is analogical after other preverbs and that the change occurred as late as the immediate prehistory of Irish.

(1) The prototonic stem of OIr. *in·loing* ‘to join, unite’ is *·ellaing* < PC * *en-lunge-*, its verbal noun is *ellach* < * *en-lou̯go-*.

(2) OIr. *ellam* ‘ready, prompt’ and ‘bride-price’ can be analysed as a compound of PC * *en-* ‘in’ + * *lāmā*- ‘hand’.

(3) In Stifter ( 2011b: 558–559), I proposed to explain the legal term *noíll* ‘oath’ as a Proto-Goidelic compound * *nou̯en-lug-* ‘nine-oath’.

### 3.7. *rl > *ll?

I know of two proposals for the assimilation of * *rl* > * *ll*, neither of which is entirely compelling.

(1) Thurneysen (GOI 236) explains W, Bret. *gwell*, Corn. *guel* ‘better’ from * *u̯erlo-*, from the same PIE root * *u̯er-* ‘top’ that gives OIr. *ferr* ‘better’ < * *u̯erso-* ( 3.2. (7)), but with a different suffix.

(2) Schrijver ( 1995: 421–422) derives OIr. *sell* ‘iris’, *sellaid* ‘to see, perceive’, MW *syllu* ‘to gaze’, Bret. *sellout* ‘to watch’ from PC * *stillo-* < pre-Celtic * *stīrlo-*, a derivative from PIE * *h₂stēr-* ‘star’, *via* assimilation and an Osthoff-type shortening of the vowel. A semantic parallel for the metaphorical use of a heavenly body for ‘eye’ is PIE * *suh₂lih₁* ‘two suns’, which underlies OIr. *súil* ‘eye’. Pedersen (VKG i 78–79), on the other hand, compares Gr. στιλπνός ‘brilliant’ ( 2.5. (4)).

### 3.8. *ld > *ll?

None of the examples for the notion that * *ld* became * *ll* in Proto-Celtic are conclusive, and there is one strong argument against it. I will start with the alleged examples in favour of that change.

(1) The precursor of OIr. *caill* ‘forest’, W *celli*, OCorn. *kelli* ‘grove, copse’ has been reconstructed as PC * *kaldi-* and compared with PGerm. * *hulta-* ‘wood’ < * *kl̥do-*. However, PC * *kalnV-* would equally be possible, perhaps derived as ‘place for making lumber’ from the root * *kelh₂-* ‘to strike’, with unclear loss of the laryngeal. See Zair ( 2012: 182–183) for further speculations.

(2) OIr., W *coll*, OCorn. *colled*, Bret. *koll* ‘destruction, damage’ has been compared with PGerm. * *halta-* ‘lame’ < PIE * *kol(h₂)do-*, from the same root * *kelh₂-* ‘to strike’ as the preceding item. Because of the semantic distance between the languages, it is not necessary to operate with identical formations. Like before, the Celtic word could continue a formation with a nasal suffix, *i.e.*, * *kolh₂no-* with loss of the laryngeal by Saussure’s Law. Thurneysen (GOI 95) and Hamp ( 1974e: 196) compare Lat. *culpa*, but the notion of PC * *ll* < * *lp* was rejected in section 2.5. Hamp also sets up the alternative reconstruction * *kol(d)no-* without further discussion.

(3) OIr. *sall* ‘bacon’ could be derived from the stem * *saldo-* < PIE * *sh₂l-dh₃-o-* (see NIL 586–587 for this reconstruction), but a formation with a nasal suffix, *i.e.*, * *sh₂l-n-*, is equally possible and has been adopted in section 2.1. (9). Other evidence for * *sald-* in Celtic, namely W *hallt* ‘salt, salty’, is ambiguous. British Celtic did undergo the relatively late sound change * *ld* > *llt*, which also affected loanwords from Latin. Latin loans furnish the most solid instances for this change: W *swllt* ‘shilling’, Corn. *sols*, Bret. *saout* ‘money’ < VLat. *soldus* < *solidus*, W *melltith* ‘curse’ < *maldictio* < *maledictiō*. A possible native item is W *mellt* ‘lightning’ < * *meldʰV-* ‘lightning, hammer of the god of thunder’. While * *saldo-* is a conceivable preform of *hallt*, that word could also continue * *sal-* + adjectival * *-to-*, *i.e.*, ‘provided with * *sal-*’. Therefore, neither Irish nor Welsh provide unambiguous evidence for a Celtic preform * *saldo-*. If *hallt* continued * *sald-*, it would disprove the Proto-Celtic age of the assimilation * *ld* > * *ll*.

(4) In any case, PIE * *meldo-* ‘mild, soft’ > OIr. *meld*, later *mell* ‘pleasant’ is decisive evidence that the cluster was retained unchanged up to Old Irish.

The changes in section 3.1.– section 3.8. were major additional sources of geminate resonants, especially in the Insular Celtic languages. Although they include no obstruent sounds, through their number they reinforced geminates as a phonological class in Celtic. Kuryłowicz ( 1957: 141‒144) makes a similar point about the pivotal role that geminate resonants arising from soundchange played in establishing gemination as a marked phonological category, but his arguments for gemination as a morphological process as a whole are not convincing and rely only on a small number of examples.

### 3.9. *‘tau Gallicum’*

In this section, a diversity of phonologically related clusters are treated together, although they belong to several different chronological layers. Clusters of dentals involving voiceless dentals, of dentals followed by *s*, and of *s* followed by *s* across a morphological boundary, are grouped together under a single heading, glossing over various issues that are not relevant to the present question. Already at the Proto-Indo-European stage, clusters of dentals developed an excrescent medial sibilant on an allophonic level ( section 3.9.1– section 3.9.2.). These clusters merged with clusters of dentals with original * *s* over the course of the ancient Celtic period ( section 3.9.3– section 3.9.6. In the ancient languages, their behaviour and orthographic representation is still differentiated, which indicates that they had not all fallen together indiscriminately yet. Depending on their origin, they may appear as biphonemic assibilated or as geminate sibilant sounds, traditionally referred to as *tau Gallicum* (see Eska, 1998 for more details). All these clusters end up as *s* in the Insular Celtic languages, *via* an intermediate stage of geminate or ‘strong’ * *ss*.

The general outlines of these developments are well-known ( *e.g.*, McCone, 1996: 48, 99; Stifter, 2017: 1192); the intricate minutiae do not need to detain us in this article, which focusses on the bigger, systemic picture of Celtic sound developments. Seven different types of input lead to the same outcome in the Insular Celtic languages. Completeness is not envisaged here, only a few notable examples will illustrate each type.

3.9.1. *tt > *tˢt > *tˢ > ss

(1) qPIE * *ret⁽ˢ⁾-ti-*, * *ret⁽ˢ⁾-tu-* ‘running’, an abstract noun of the verbal root * *ret-* ‘to run’ > * *retˢi/u-* > * *ressi/u-* > Gaul. *ressi-*, W PN *Rhys*, OIr. unstressed compound element *-ras*/ *-rus*.

(2) PIE * *h₂lit⁽ˢ⁾-tu-* ‘suffering’ > * *litˢu-* > * *lissu-* > OIr. *lius* ‘disgust, loathing’.

(3) The PIE root * *u̯et-* ‘to turn to’ formed the past participle * *u̯et⁽ˢ⁾-to-*, which *via* * *u̯etˢo-* > * *u̯esso-* constitutes the root of the OIr. impersonal verb *do·cuäs* ‘has gone’.

3.9.2. *dt > *dˢt > *tˢ > ss

(1) PIE * *med⁽ˢ⁾-tu-* ‘mast, nourishment’ > * *metˢu-* > * *messu-* > OIr. *mess*, W *mes*, Corn. *mesen*, Bret. *mez* ‘acorn, mast’.

(2) PIE * *med⁽ˢ⁾-tu-* ‘judgement’ > * *metˢu-* > * *messu-* > OIr. *mess*, W *armes* ‘prophecy’.

(3) PIE * *k̑h₂d⁽ˢ⁾-ti-* ‘strong emotion’ > * *katˢi-* > * *kassi-* > Gaul. *cassi-*, OIr. *cais* ‘love, hate’, W, Corn., Bret. *cas* ‘hatred’.

(4) PIE * *u̯id⁽ˢ⁾-to-* ‘seen’ > * *u̯itˢo-* > * *u̯isso-* > CisGaul. *-uiśeos*, Gaul. *-uistus*, OIr. *fess*, W *gwys*, Bret. *gous* ‘was/is known’ illustrates a participal formation from a verbal root in *-d*.

3.9.3. *Ds > *tˢ > ss

Proto-Celtic must have possessed a large number of examples of this change as part of the morphologically regular formation of the *s*-subjunctive of strong verbs, and of the related formation of the future (continued in Old Irish) and the desiderative (attested in Gaulish). Here I will limit myself to the subjunctive, which was formed by adding the suffix * *-se/o-* directly to the final consonant of the root ( Schumacher, 2004: 49–57). Due to the phonotactic constraints of Celtic, this then underwent various types of changes. In the case of dentals, the result in Proto-Celtic was *tau Gallicum*. Because of the fragmentary or relatively late transmission of most Celtic languages, evidence for this formation is only plentifully attested in Old Irish, but sporadic evidence is found in the other languages as well. Two examples will suffice as an illustration for Irish:

(1) PIE * *ret-se/o-* > * *retˢe/o-* > OIr. *ress-* ‘may run’.

(2) PIE * *g ^u̯^ʰed-/se/o-* > * *g ^u̯^etˢe/o-* > OIr. *gess-* ‘may ask, pray’.

British and Continental Celtic only have residual examples of this category.

(3) A possible case in Gaulish is *scrisumio* ‘that I may spit’ < * *skritˢe/o-* < PC * *skrit/d-se/o-* (determining the precise Indo-European root is more challenging, cf. Darling, 2019: 133–137).

(4) In Celtiberian, *robiseti* ‘may cleave (?)’ may continue * *bitˢe/o-* < * *bʰidʰ-se/o-* from the root * *bʰei̯dʰ-* ‘to split, cleave’ ( Schumacher, 2004: 224–225).

It is conceivable that this category was still productive in the ancient Celtic languages and that new finds of texts will furnish more examples in the future.

(5) In British Celtic, the category had been largely abandoned by the period of medieval attestation. The only lexical fossiles are MW *gwares* ‘he may succour’ and *ryres* ‘he may run’, two compounds of * *ret-se/o-*.

3.9.4. *zds- > *tˢ > ss

The only example for this highly specific development is * *nezd-is-m̥h₂o-* > * *nezd ^†^samo-* ‘nearest, next’ > * *netˢamo-* > Gaul. gen.pl. *neđđamon*, OIr. *nessam*, W *nesaf*, Corn. *nessa*, MBret. *nessaff* ‘nearest, next’ ( Jasanoff, 1988–90: 185). In theory, PC * *nezd-tamo-* with a different superlative suffix would also be conceivable.

3.9.5. *st > *tˢ > ss

(1) PIE * *upo-sth₂o-* ‘the one standing underneath’ > * *u̯osto-* > * *u̯otˢo-* > * *u̯osso-* > Gall.-Lat. *uassus*, OIr. *foss* ‘servant’, W *gwas* ‘boy, lad, servant’, Corn. *guas*, Bret. *gwaz* ‘young man’.

(2) PIE * *h₂u̯osto-* ‘staying’ > * *u̯osto-* > * *u̯otˢo-* > OIr. *foss* ‘rest, position’, W *gwas* ‘abode, dwelling’.

(3) PIE * *k ^u̯^is-to-* ‘seen’ > * *k ^u̯^itˢo-* > OIr. *ad·cess* ‘was seen’ illustrates a participal formation from a verbal root ending in *-s*.

(4) If the identification of Gaul. *tuððos*/ *tuθos*/ *tuso* with Lat. *furnus* ‘furnace’ is correct (see Delamarre, 2003: 304), then the word could be derived from * *tutˢo-* < * *t(o)-us-to-*, from the root * *h₁eu̯s-* ‘to burn’.

The British languages sometimes have *st* where the other Celtic languages have *ss*. Schrijver ( 1995: 410–430; 2022) explains such cases as continuing * *-sst-*, *i.e.*, clusters that arose when a suffix with *-stV-* was added to stems or roots ending in *s* or, occasionally, a dental. The evidence for this morphological structure is circular, since it is only recoverable from the fact that the British languages have *st*. Possible instances are:

(5) qPIE * *h₂u̯es-steh₂-* ‘spending the night’ > * *u̯esstā-* > OIr. *fess*, but W, Bret. *gwest* ‘night’s stay, feast’.

(6) PC * *kisstā-* ‘woven basket’ > OIr. *cess* ‘basket, wickerwork’, Lat. *cissium* ‘a light, two-wheeled vehicle, cabriolet’, borrowed from Gaul. * *cission*, but W *cest*, Bret. *kest* ‘basket, box’ from the root PC * *kei̯s-* ‘to do, bring, plait’ ( Schumacher, 2004: 391–393; see 3.9.6. (4)). It is not excluded, however, that this noun was influenced by, or even borrowed from Lat. *cista* ‘box’.

3.9.6. *s-s > *ss

Although inherited clusters of two consecutive *s* were simplified to a single *s* according to a well-known Indo-European sound rule, where such a formation was synchronically transparent, double *ss* could be reintroduced in the individual Celtic languages.

(1) This is directly seen in Gaul. *pissiumi* ‘I shall see’, which synchronically consists of the Gaulish verbal root *pis-* < PIE * *k ^u̯^ei̯s-* ‘to perceive’ + the desiderative or future morpheme * *si̯e/o-*.

The subjunctive and future stems of most roots where this rule could apply (PC * *gus-* ‘to choose’ < * *g̑eu̯s-*, * *k ^u̯^is-* ‘to see’ < * *k ^u̯^ei̯s-*, * *tau̯s-* ‘to be silent’ < * *th₂eu̯s-*, * *u̯os-* ‘to spend the night’< * *h₂u̯es-*, all after Schumacher, 2004) have been rebuilt in such a way that the phenomenon can no longer be observed in Irish.

(2) Only stray examples with *s* remain in the case of of *ad·cí* ‘to see’ < * *k ^u̯^is-*, *e.g.*, OIr. 3sg. *·accastar* ‘is seen; may be seen’, or future forms such as 1sg. *·accus* or 3pl. *at·chichset* < * *k ^u̯^ik ^u̯^is-se/o-*.

(3) Schumacher ( 2004: 525–527) sets up a root PC * *φlus-* ‘to drink’, which he derives from *? *pleu̯s-*, tentatively explained as an extended form of PIE * *pleu̯-* ‘to swim, float’. The subjunctive stem of this verb in Old Irish is *lós-* and *lús-*.

(4) Finally, Schumacher ( 2004: 391–393) sets up the root PC * *kei̯s-* ‘to make, accomplish, create’ (see also 3.9.5 (6)). No present stem of this verb is attested in Old Irish; the subjunctive stem *céiss-* ‘may plait, make, bring’ and the future stem *cichis-* appear to exhibit *-s-* originating from double * *s-s*.

3.9.7. ‘Original’ *ss

The etymological origin of *tau Gallicum* is not always clear. Occasionally its origin may have been onomatopoetic. A case in point is Gaul. *bussu-* if it means ‘kiss’ or ‘lip’, which seems to have a parallel in Irish ( *Bérla na Filed*) *bus*, *pus* ‘lip’ and in Southern German *Busserl* ‘kiss’.

### 3.10. Stops plus *n?

Sequences of stops + * *n* have been claimed by some scholars to be a source for geminate stops (‘Kluge’s Law’; *e.g.*, Stokes, 1891; Stokes, 1891–3, Zupitza 100; VKG i 158–161; Lühr, 1985; Bammesberger, 1998; see the section on previous research in the introduction). It is not possible in the context of this study to discuss all the proposed items (for example, Lühr, 1985 discusses 72 relevant forms). Suffice it to say that the proposals are rarely convincing (cf. GOI 92‒93), that they can be accounted for in alternative ways, or that they face counterexamples (see the discussions by Kuryłowicz ( 1957: 131‒132) and Strachan (1891‒4)). For almost every geminate occlusive, a combination of that same simple occlusive + *n* can be found that must be reconstructed as such for Proto-Celtic and which therefore runs counter to the predictions of Kluge’s Law in Celtic. ^2^ I will illustrate these general objections with only a few examples.

(1) OIr. *ette* ‘wing’ has been frequently traced back to * *petnii̯o-*, but this is flatly contradicted by OIr. *én*, W *edn*, Corn. *ethen*, MBret. *ezn*, ModBret. *evn* ‘bird’, which can only continue * *φetno-* < * *petno-*. OIr. *ette* may rather come from a formation with a different suffix, *e.g.*, * *φetantii̯o-* << * *pet-n̥t-* ‘flying’; cf. OIr. *ethait* ‘bird’ < * *φetantī-* < * *petn̥tih₂-*. OBret. *attanoc* ‘winged, flying’ is not an example of geminate *-tt-* (this would have given * *th*!), but rather a variant spelling for *-d-*, cf. ModBret. *adaneg* ‘winged’ < * *φatanāko-* << * *pₔt-n̥-*.

(2) Gaulish attests to countless examples of the patronymic suffix *-ikno-* ‘son/daughter of’ < * *i-kn-o-*, whose semantic core is the PIE root * *ken-* ‘to originate from’. The common ancient Celtic onomastic suffix * *-kko-* has nothing to do with this, but is rather due to ‘onomastic’ gemination of the common adjectival suffix * *-ko-*, in analogy to other onomastic suffixes with geminates, such as *-illo-* < * *-ilno-* or *-ullo-* < *- *ulno-* (see chapter 9.).

(3) On a related note, the complex suffix * *-o-g̑n̥(h₁)-o-* ‘born from’ regularly develops to * *-agno-* in Goidelic. This is attested in ogam inscriptions as *-AGNI*, and as the common suffix *-án* in Old Irish, not ** *-aggo-* > OIr. ** *-ac*, as would be predicted by Kluge’s Law. I do not enter the vexed question whether * *-agno-* is regularly reflected as *-an* in British Celtic, or whether that suffix has a different origin.

## 4. Etymological gemination: developments across branches with divergent outcome

A small number of simplifications or assimilations of clusters led to very different outcomes in the sub-branches of Celtic, or they occur so sporadically that they are better classified as isolated developments rather than proper sound changes.

### 4.1. *zd

Proto-Celtic * *zd* continues the rare PIE cluster * *sd*. No evidence is known how this cluster was treated in Celtiberian. The ultimate outcome of * *zd* in Old Irish is ⟨t⟩ = /d/, apparently *via* an intermediate stage * *dd*. How early this assimilation occurred is unclear. In the case of * *zg*, the spelling TASEGAGNI, probably for OIr. *Tadgán*, in the ogam inscription I-KIK-002 (= CIIC 28) seems to indicate the retention of the sibilant until very recently before Old Irish. The ultimate outcome of * *zd* in the British Celtic languages is * *θ* (W ⟨th⟩, Corn. ⟨th⟩, Bret. ⟨zh⟩), which is also the regular outcome of * *tt*. A stage * *dd* like in Irish is precluded since that yields *d* in the British Celtic languages. One way of explaining the British development is to assume that * *zd* passed through the intermediate stage * *tt*, even though the required change * *zd* > * *tt* is untrivial. It finds potential support in Gaulish where the stage * *tt* appears to be attested in Gall-Lat. *petia*, *pettia* ‘piece’, which can hardly be separated from PC * *k ^u̯^ezdi-* ‘part, share’, and in Gall-Lat. *bottia* ‘blister, bump, boss’ and * *botto-* ‘knob, button’, which underlies various words in the Romance languages and which could be cognate with OIr. *bot*. Isaac ( 2004: 74–76) proposes a different pathway from PC * *zd* to Brit. * *θ* and the Gaulish reflex. Schrijver ( 1995: 376) is agnostic about the intermediate stages in the development.

(1) PIE * *ni-sd-o-* ‘place to sit down’ > * *nizdo-* ‘nest’ > OIr. *net*, W *nyth*, OCorn. *neid*, LCorn. *nyth*, OBret. *nith*, Bret. *neizh*.

(2) PIE * *bʰrosdʰo-* > * *brozdo-* > OIr. *brot* ‘goad, spike’, W *brath* ‘biting, stinging; prick’. The vocalism of the Welsh word is unclear.

(3) PIE * *gu̯osdo-* ‘tail’ > * *g ^u̯^osdo-* > * *bozdo-* > Gall-Lat. *bottia* ‘blister, bump, boss’, OIr. *bot* ‘tail, penis’, W *both* ‘nave, hub, boss of a shield’, Corn. * *both* ‘hillock (in placenames)’. However, the precise relationship of these words remains to be clarified.

(4) PIE * *g̑ʰasdʰo-* > * *gazdo-* > OIr. *gat* ‘withe, osier’.

(5) PC * *k ^u̯^ezdi-* ‘part’ > Gall-Lat. *pettia* ‘piece; share of land’, OIr. *cuit* ‘share, part’, OW *ped*, W *peth*, MCorn. *peth*, *pyth*, Bret. *pezh* ‘thing’, Pict. *peth-* ‘share of land’.

(6) qPIE * *masdi̯o-* > * *mazdi̯o-* > OIr. *maite* ‘stick, staff’. This may be a loan from a pre-Indo-European substrate language ( Van Sluis *et al*., 2023).

(7) PIE * *rasd-* ‘to scratch’ > * *razd-* > W *rhathu*, Bret. *razhañ* ‘to scratch, rub off’.

(8) PIE * *su̯isde/o-* ‘to whistle’ > PC * *su̯izde/o-* > W *chwythu* ‘to blow, breathe’, Corn. *hwytha*, Bret. *c’hwezhañ* ‘to breathe’; and the nominal formation PC * *su̯izdo-* > W *chwyth*, Corn. *wheth*, Bret. *c’hwezh* ‘breath, breeze’. OIr. *fet* ‘whistle’ could belong here as well if the lenited anlaut *f-* < * *su̯-* became generalised instead of expected *s-*. Alternatively, *fet* can come from * *u̯into-* < * *u̯īnto-* < * *u̯eh₁nto-* ‘wind’ (see section 7.2.).

(9) PC * *trozdi*- ‘starling’ > OIr. *truit* ‘starling’. The cognates W *trydw*, OCorn. *troet*, LCorn. *trojen*, OBret. *trot*, MBret. *tred* ‘starling’ are possibly loans from Irish (IEW 1096; *pace* Stifter, 2021: 174), unless the Insular Celtic languages continue * *troddi-*, which is unsatisfactory with regard to the Germanic cognates ON *þrǫstr* < * *þrastu-*, OEngl. *þræsce* ‘thrush’ < * *þra(st)skōn-*, as well as cognates in other European languages. This item is suspect of being a prehistoric substratal loan into several European languages.

### 4.2. *χt

A handful examples appear to attest to the irregular assimilation of PC * *χt* (of diverse origins) > * *tt*, instead of retaining them as two separate segments as is the norm. Since examples for this assimilation are isolated across the family and within the languages, it is best to view them as sporadic simplifications of the clusters, the motivation for which remains obscure.

(1) The name of the Ibero-Celtic people *Vettones* in Central Spain can be understood as * *u̯eχt-on-*, a form with the individualising suffix *-on-* built on the stem seen in Gaul. *Vectirix*, *Vecturius*, OIr. *fecht*, MW *gweith* ‘journey, time’, Corn. *gweth*, *gywth*, Bret. *gwezh* ‘time’ < * *u̯eg̑ʰ-to-* ( Prósper, 2005: 305–309).

O’Brien ( 1954; 1956) has drawn attention to three Irish words that appear to exhibit a similar behaviour:

(2) OIr. *utlach* ‘lapful’ stands beside *uchtlach*, synchronically derived from *ucht* ‘lap’ < PIE * *pektu-* ‘breast’. It is conceivable that the internal *-ch-* was lost through dissimilation against the final *-ch*.

(3) OIr. *littiu* ‘gruel, porridge’ stands beside *lichtiu* and W *llith* ‘food, sustenance’ < * *liχti̯on-* (of uncertain origin). Matasović ( 2009: 135) regards the form with *-tt-* as primary and reconstructs PC * *φlittV-*, which he compares with Lat. *puls*, *pultis* ‘porridge’ < * *polt-*/ *pl̥t-*.

(4) OIr. *aittenn* ‘furze, gorse’ can be reconstructed as * *attīno-*, but its cognates W *eithin*, OCorn. *eythinen*, MCorn., OBret. *ethin*, Bret. *izhin* continue * *aχtīno-* < PC * *ak-tīno-*, perhaps ‘sharp tree’, from the root * *h₂ek̑-* ‘sharp, pointed’. McCone ( 2005: 409) compares this with Basque *ote* of the same meaning and suspects a borrowing from an unknown source for all languages.

## 5. Etymological gemination: morphological gemination in derivation and inflection

Many instances of gemination treated above ( *e.g.*, * *ln* > * *ll*, * *sm* > * *mm*, * *sn* > * *nn*) occur across morpheme boundaries and are in fact indirectly the result of processes of word formation. Evidence for a few more such changes arising through derivation or inflection is usually restricted to a single branch within Celtic. Therefore, it will be apposite to discuss them separately.

### 5.1. *n-n > *nn

A group of words with *nn* in the Celtic languages are best analysed as derivates with a nasal suffix from roots ending synchronically in *n*. Although the phenomenon appears to be pan-Celtic, the evidence for it is often confined to a single language or two languages at best. It is noteworthy that the resulting sequence *-nn-* was not simplified, as may have been the case with early instances of * *-mm-* > * *-m-* (see 6.3. (2)). Perhaps these words were formed at a time when geminates were already established as a phonological class in the language.

(1) The double *nn* in the Gaulish personal names *Adgennos*, *Congennolitanos*, *Adgonnetios* has in the past either been ignored or explained as a sporadic gemination of * *-geno-* < * *g̑enh₁o-* ‘born’ in personal names. However, onomastic gemination is typically coupled with shortening, but none of these names show shortening. Furthermore, all examples of *-nn-* are found in compounds with preverbs as first members, whereas compounds with other elements, *e.g.*, Cib. *mezukenos*, Gaul. *Medugenos* ‘mead-born’, *Rextugenos* ‘law-born’, OIr. *Muirgen* ‘sea-born’, *etc*., never show gemination. The two phenomena seem to be causally linked. I propose to explain *-genno-* as a thematic derivative of the Celtic neuter *n*-stem * *genen-* ‘birth, origin’ (cf. OIr. *gein*), namely * *-gen-n-o-* ‘having birth, origin’. In this interpretation, *Adgennos* can be understood as meaning ‘having birth into (a specific family), having been born to (a family)’, and *Congennolitanos* as ‘being wide-ranging with regard to relations’. PC * *genen-* itself continues earlier * *genmen-* with loss of *m* in heavy clusters such as * *genmn-*; * *genmen-* in turn is simplified from PIE * *g̑enh₁men-* (NIL 153).

(2) Gaul. *linna* ‘coat’, OIr. *lenn*, W *llen*, OCorn. *len* ‘coat’, OBret. *lenn* ‘piece of cloth’ all go back to PC * *linnā-*. The proposed etymology as * *φlitnā-* ‘spread out (cloth)’ ( Delamarre, 2003: 203) is unsatisfactory since * *tn* does not otherwise assimilate to * *nn*. Perhaps it is to be analysed as * *lin-neh₂-* ‘made of linen’ (cf. Gr. λίνον ‘linen’, OCS *lьnъ* ‘flax’) or as * *liHn-neh₂-* with Osthoff shortening before the geminate (cf. Lat. *līnum* ‘flax, linen’ and OIr. *lín*, unless the latter is borrowed from Latin).

(3) The Gaulish name element *rinno-* of unknown meaning, the rare Irish adjective *renn* ‘swift, hasty’, and W *rhyn* ‘rigid, stiff, brave, rough, cold’ may form an equation, although the divergence in meaning renders this less than obvious. If the Irish adjective represents the original meaning, one can think of a formation from the root * *h₃rei̯H-* ‘to whirl’, perhaps from a hypothetical nasal-infixed verb * *rinati* ‘to run’, comparable with Goth *rinnan* < * *h₃ri-n-H-*, with the addition of the adjectival suffix * *-no-* to the present stem. Alternatively, the formation could derive from the noun * *rei̯no-* ‘great flowing mass of water’ < * *h₃rei̯(H)-no-*, with ablaut and with the addition of a second nasal suffix.

(4) The Celtiberian name *Stennoco* (in Latin script) is a derivative of the shape * *sten-n-o-* from the *on*-stem name *stenū* < * *stenōn* with a single *n* ( Jordán Cólera, 2019: 613–614). The name ⟨stena⟩ in Celtiberian script could likewise stand for * *stennā*, since the vernacular script does not graphically mark geminates. This development is parallel to that of the genitive *abulos* < * *abul-nos* in section 2.1.

(5) The first element of Gaul. *sonnocingos* has been thought to contain the oblique stem of the Indo-European word for ‘sun’. If this doubtful proposal is right, the structure of the word must be something like * *su(h₂)n-no-*, *i.e.*, a derivative with a nasal suffix (which still would not explain the vowel *o*).

(6) Old Irish has two distinct verbs that influenced each other formally: in PIE * *sn̥-n(e)-h₂-* ‘to obtain’, a nasal-infix present of a root containing * *n* led to geminate * *nn* in * *sanna-* → * *sanne/o-* > OIr. *seinnid*. Schumacher ( 2004: 558) defines its meaning as ‘to attain, achieve, procure’; in eDIL ( https://dil.ie/36945), the meaning is given as ‘to strike’, with many examples that are classified as “obscure”. Although entirely unrelated, PIE * *su̯n̥h₂-* ‘to sound’ > * *su̯ana-* acquired geminate * *nn* probably after its model, *i.e.*, * *su̯anne/o-* > OIr. *seinnid* ‘to sound, make music’.

(7) In British, the same development underlies the verb PIE * *tn̥-nu-* > * *tannu-* > W *tannu* ‘to extend’, Corn. *tan* ‘take!’ ( Schumacher, 2004: 618–619).

### 5.2. Other

It is conceivable that (very rare) combinations of nominal stems ending in * *-b* + the athematic dative and instrumental plural endings * *-bis* and * *-bos* may have led to geminate *- *bb-* within nominal paradigms, but no such examples are attested from ancient Celtic. Probably there were other, rare contexts of similar character in which geminates arose through regular processes of inflectional and derivational morphology.

## 6. Etymological gemination: morphological gemination in composition

Compounding, that is the univerbation of two or more lexical items to form a new lexical item that is more than just the sum of its constituent parts, is an important process of word formation of ancient and medieval Celtic languages. When in the course of compounding two consonants come into contact with each other, new geminates can emerge, either because the consonants were identical or similar already before, or because assimilation takes place.

### 6.1. *dzd* > *dd*?

In Stifter ( 1998: 212–218), I proposed to explain PC * *ruddo-* ‘rust’, reflected in W *rhwd*, Corn. *-roade*, *-rode* in placenames, OBret. *rod* ‘rust; mud, dirt’, either as a * *h₁rudʰ-dʰh₁ó-* ‘that which makes redness’ or as * *h₁rudʰs-dʰh₁ó-* > * *rudzdo-* > * *ruddo-*, where the first element is an *s*-stem with zero-grade in root and suffix and with subsequent simplification of the cluster * *dzd*. The alternative proposal by Hill ( 2003: 196–202) < * *h₁rudʰ-sd-ó-* with the root * *sed-* ‘to sit’ is phonologically equivalent to the second option. Schaffner ( 2016–7: 114–5), on the other hand, reconstructs * *h₂ru-ti-* ‘redness’, which does not involve a geminate.

### 6.2. Assimilations across morpheme boundaries

A special source for geminates are obstruent clusters where the first obstruent assimilates totally to the articulation of the second. The simplest examples are those where the involved sounds are already identical from the outset. Relevant examples are Gaul. *readdas* ‘has given’, perhaps from * *φro-ad-dast*, and the very similar OIr. *do·rat* ‘has given’ < * *to-φro-ad-dast*, both ultimately containing a sequence of the preverb * *ad-* and the PIE root * *deh₃-* ‘to give’. Welsh provides the examples *adynu* ‘to suck’ < * *ad-dina-* ( Schumacher, 2004: 274), *adygaf* ‘to take, seize’ < * *ad-duke/o-* ( Schumacher, 2004: 287), and perhaps the Welsh adjective *edifar* ‘regretted, deplored’ < * *ad-dī-maro-* (cf. Jackson, 1953: 427). In these cases the synchronic outcome of the geminate in Welsh is an unlenited voiced stop. The late by-form *addygaf* of *adygaf* owes its lenition to the influence of other compounds of * *duk-* after leniting preverbs, *e.g.*, MW *dyđwc* ‘to bring, carry’ < * *to-duk-*, *ymđuc* ‘to conceive, bear’ < * *ambi-duk-*, *kyfyrđuc* ‘to remove’ < * *kom-ro-duk- etc*. ( Schumacher, 2004: 287). Such reanalyses are found elsewhere as well: the compound *atygaf* ‘to bring back’ ( *e.g.*, MW *attwc*) < * *ad-ddug-* < * *ati-duk-*, with the different preverb *ad-* < * *ati-*, received the reanalysed by-form *ad-ddygaf*. W *addysg* ‘education, learning’ is either a formation within Welsh or, if it ultimately continues Lat. *addiscō* ‘to learn in addition’, it may have undergone the same analogical lenition as *addygaf*.

In the majority of instances, however, the consonants across morpheme boundaries are different and some sort of assimilation takes place, either assimilation in voice or in articulation, or in both. This is readily observable in verbal and nominal compounds where the first element is a preverb ending in a stop, in particular * *ad-* and * *eχs-*. The latter occurs in the *s*-less allomorph * *ek-* in at least a subgroup of such formations (see the discussion in Russell ( 1988: 118‒121), how and why * *ek-* and other preverbs lost their * *-s*). Examples come from all branches of Celtic. However, the way how cross-morphemic clusters are treated differs in complex ways, not only between the branches, but sometimes even within a single language. This difficult matter deserves a detailed discussion.

It seems that already in Proto-Celtic there was full assimilation to the second obstruent when it was voiceless. An equation involving three branches of Celtic is PC * *ad-trebā-* > * *attrebā-* ‘abode, dwelling’, reflected in OIr. *attrab*, W *athref*, and in the Gaulish ethnonym *Atrebates*; cf. OIr. *ad·treba* ‘to inhabit’ where the two elements are separated by a strong morphemic boundary. This contrasts with the adverb W *adref* ‘homewards’ that belongs to a younger stratum. *Pace* GPC, which calls its initial *a-* an “elfen anhysbys” (obscure element), it is evidently the old preposition * *a* < * *ad* ‘to’ (identical with the sentential connector *a ^2^*) + * *trebā* ‘home’, corresponding to Bret. *a-dreñv* ‘backwards’. Even though *adref* contains the same etymological elements as *athref*, it must have been univerbated from a prepositional phrase when * *ad* had lost its final consonant and consequently had acquired a leniting effect. An equation encompassing Gaulish and Irish is CisGaul. *Ateporix*, Galatian Ατεπορειγος and OIr. *attach* ‘refuge’ < * *ad-tek ^u̯^o-* ‘running to’. Several other examples demonstrate that full assimilation and, most likely, gemination were in fact the rule in Gaulish in such contexts, namely *appisetu* ‘let see’ < * *ad-k ^u̯^ise-*, the names *Aclutius* < * *ad-klut-i̯o-* ‘very famous’ and CisGaul. *akluśamoualos* < * *ad-klut-(i)samo-u̯alo-* ‘most famous ruler’, and Lat. *attegia* ‘cabin, hut’, which was probably borrowed from Gaul. * *attegi̯ā-* < * *ad-teg-i̯ā-*. Old Irish furnishes numerous additional instances of voiceless geminates resulting from assimilation to a second element with a voiceless stop, *e.g.*, *frettech* ‘fore-swearing’ < pre-OIr. * *frith-tech-*, verbal noun of *fris·toing*, *accobar* ‘desire, wish’ < * *ad-kuφro-*, verbal noun of *ad·cobra*, and many others. Examples in British are not quite as numerous, but still unambiguous, *e.g.*, W *achas*, Corn. *ahas*, which correspond to OIr. *accuis* ‘hate, enmity’ < * *akkassi-* < * *ad-kad-ti-*; and W *achan* ‘whisper, murmur’ < * *ad-kanV-*, W *athwll* ‘perforated’ < * *ad-tullo-*.

The behaviour of the preverb * *ek(s)-* is special. The expected development in front of a voiceless consonant is that the first * *k*, *via* the intermediate stage * *χ*, was either lost or assimilated to the * *s*. This is shown by the agreement of Gaul. *escingo-* ‘warrior, infantrist’ < * *eχs-keng-* ‘striding out’ (but cf. also *extincon* of unknown meaning) and OIr. *escarae* ‘enemy’ < * *eχs-karant-* (however, its lack of syncope points to a recent date of formation); cf. also OIr. *sesca* ‘sixty’ < * *su̯eks-kont-*. Celtiberian provides the comparable example *eskenim*, perhaps ‘foreigner’, but in this case in front of a voiced stop, if it goes back to * *eχs-gen-i-*. For the position before * *t*, a parallel is provided by * *trek-stu-* > * *treχstu-* > * *trestu-* > OIr. *tress* ‘contention, fight’, W *tres* ‘battle’. However, in young, as it were *nachgrundsprachlich*, compounds, it seems that the consonantal part of the preverb assimilated totally to the following consonant, perhaps in parallel with the treatment before voiced consonants (see the following paragraph). This may have occurred separately in the already differentiated Celtic branches after the break-up of Proto-Celtic. Old Irish has only three relevant examples: *etaim* ‘chance, opportunity (?)’ < * *eχs-tud-sman-* or * *eχs-dī-tud-sman-* ‘act of falling out’, *ettech* ‘refusal’ < * *eχs-teg-o-*, verbal noun of *as·toing* ‘to refuse’, and *etal*, *etail* ‘pure, sinless’ < * *eχs-tol-o/i-* ‘being outside of desire’. The latter has a parallel in W *ethol* ‘chosen’ < * *ettol* < * *ek-tol-*. This treatment of * *k-t* > * *tt* across compositional boundaries contrasts with the regular development of pre-Celtic * *Kt* > PC * *χt* within simplex lexemes, and must be due to morphophonological analogy after the model of voiced sounds.

When the second element was a voiced obstruent, the outcome differs decisively between the languages and no comprehensive picture emerges. Old Irish shows full assimilation in such cases. Verbal and nominal compounds provide countless examples; it will suffice to mention a few representatives, *e.g.*, OIr. *·eipir* ‘says’ < * *eχs-ber-* or *·acair* ‘sues, accuses’ and *acrae* ‘act of suing, bringing an action’ < * *ad-gar-*. In order to explain the difference from how * *eχs-* is treated before voiceless consonants in old formations seen above, the development before voiced consonants may have been the following: * *eχs-b°* > * *eγzb°* > * *eγb°* with loss of ‘sandwiched’ * *z* and with subsequent assimilation to * *ebb-*. As OIr. *naidm* ‘act of binding, bond’ < * *nad-man-* and *maidm* ‘act of breaking’ < * *mad-man-* demonstrate, * *d* did not assimilate regularly to a following * *m*. However, in analogy to the verbal compounds cited above, * *dm* did become * *mm* in verbal compounds with *ad-* as first preverb, *e.g.*, the verb *ad·midethar* ‘to aim at, evaluate’ has the prototonic stem *·aimdethar* and the verbal noun *ammus* < * *ad-med-*. In Stifter ( 2017b: 221) I tentatively suggested that, in contrast to * *dm*, * *tm* may have regularly given * *mm* in Celtic, in order to explain OIr. *amm* ‘time’ < * *amman-* < PIE * *h₂et-men-* ‘the act of going around’, but alternative explanations are available for this word, for which see the cited article. The case of the compound * *eχs*- *med-* is ambivalent. From its prototonic stem a neo-simplex verb *éimdid* ‘to refuse, reject’ was created, which is frequently written with *mh*, indicating lenition of the *m*. On the other hand, the further-derived compound *fo·émid*, *for·émid* ‘to be unable, fail; refuse’ never shows such a spelling and is in fact once transmitted with a double *mm*. One of the two stems must have undergone analogical change.

The evidence is not so resounding in sheer numbers in British, and it seems to differ between the classes of sounds involved and according to chronological layers of word formation. Clear examples of full assimilation are found for the preverb * *ad-* followed by *b-*. W, Corn., Bret. *aber* ‘river-mouth’ < * *abber-* < * *ad-ber-* is reflected the same in all three languages and must be an old formation; W *aberth* ‘sacrifice’ < * *ad-ber-tā-* and W *abwyd* ‘bait, lure’ < * *ad-* + *bwyd* ‘food’ show the same treatment. However, the evidence for *-d* followed by a *g-* is ambivalent. Some examples with * *ad-* in intensifying and preverbal function display the plain simplification of the geminate: W *agarw* ‘rough, stern, bitter’ < * *ad-garu̯o-* (cf. OIr. *acarb* ‘very rough’), W *agwrdd* ‘strong, mighty’ (related to Hisp.-Lat. *gurdus* ‘dolt’?); W *agwedd* ‘manner, fashion’ (beside *gwedd* ‘appearance’ < * *u̯idā-* ‘look’) can only have been formed after initial * *u̯-* had developed into an occlusive. On the other hand, a handful of examples appear to illustrate first assimilation of * *dg* to * *gg* and then fricativisation to * *χ*, effectively making it fall together with the outcome of * *kk*. These are the hapax W *achwir* ‘true, genuine’, seemingly a compound of * *ad-* + *gwir* ‘true’, W *achlan* ‘all, total’, if it is from * *ad-* + *glan* ‘clean’, and W *achwre*, *achre* ‘part of a roof or fence; covering’, perhaps from * *ad-* + * *u̯regi-*, related with OIr. *fraig* ‘wall’. Again several of these words feature *g-* that arose word-initially rather late before PC * *u̯-*. Compounds of the root PC * *gab-* ‘to take’ do not provide conclusive evidence. Schumacher ( 2004: 321) argues that the inherited stem was replaced by * *kab-* in British, *e.g.*, W *dyrchafael*, MCorn. *drehevel* ‘rising, ascending’ continue * *to-ro-ud-kab-aglā-* rather than * *-ud-gab-*.

The outcomes are also rather diverse for the preverb * *eχs-*. It is evident that some of the developments must be due to analogy, but it is difficult to determine with certainty what the regular inherited treatment was. Some examples suggest that in such cases the outcome was a fricative, *e.g.*, W *differ*, MCorn. *difres* ‘to defend, protect’ < * *dī-eχs-ber-*, and W *dichlyn* ‘to choose, pick’, Bret. *dilenn* ‘to choose, select’ < * *dī-eχs-glenn-* (cf. OIr. *as·gleinn*, *·eclainn* ‘to examine’ < * *eχs-glenn-*; see Hamp, 1974c and Hamp, 1974d). Fleuriot ( 1964: 138) cites OBret. *diclinatuiu*, gl. *legendae* ‘to be chosen’, without indicating the source. The OBret. spelling ⟨cl⟩ is ambiguous and could stand for /χl/ or /gl/. I regard this verbal adjective as the Old Breton representative of ModBret. *dilenn*. Fleuriot, however, explains it as the equivalent of OIr. *do·gleinn* ‘to select, collect, gather’ < * *dī-glenn-*. This matter remains ambiguous.

Even though I have been writing these reconstructions with a medial *-s-*, chiefly for reasons of etymological transparency, it is not certain that the *-s-* was retained or, if it was, if it played a role in the sound changes. Schrijver ( 1995: 376; 1999: 2), on the other hand, suspects that the development in such contexts was “* *-ksb-* > * *-xsb-* > * *-sb-* > * *-hb-* > * *-p-* > * *-f-*” (and analogically for * *-ksg-*), *i.e.*, that *-s-* played a crucial role. In view of W *achlan* < * *agglan* < * *ad-glano- etc*. mentioned above (if that is the right explanation), I consider the possibility that these British forms involved the asigmatic allomorph * *ek-* of the preverb (see the following paragraph), with full assimilation to the initial of the following element, *i.e.*, * *dī-ek-glenn-* > * *dīgglenn-* > * *dīχlenn- etc*. In view of *aber* < * *abber-* < * *ad-ber-*, it must then be assumed that the case of the labial in W *differ* < * *dībber-* < * *dī-ek-ber-* is analogical to the guttural. Another piece of evidence for the development of * *eχ(s)-g°*, unfortunately without insights into the crucial intermediate stages, is MBret. *elas* ‘gizzard, liver’, corresponding to OIr. *eclas* ‘gizzard, oesophagus, stomach’ < * *eχ(s)-glasso/ā-* ( Hayden & Stifter, 2022). Because of the regular disappearance of the guttural before *l* in Breton, it permits no inference about the precise treatment of the cluster.

That there existed an asigmatic allomorph * *ek-* in British Celtic, or at least in the stage immediately preceding Welsh, is evident from other formations. This allomorph is most conspicuously visible in W *eglwg* ‘conspicuous, visible’ < * *ek-luko-* from the root * *leu̯k-* ‘to become bright’. Moreover, a series of words starting with *e-*, all exclusive to Welsh, can conceivably be analysed as compounds with * *ek-*, giving evidence of a sequence of reanalyses of such formations. They were therefore probably only formed productively within the Welsh language during the historical period. They include *eglan* ‘sea-shore’ < * *egglannā-* < * *ek-* + * *glannā-* ( *glan* ‘shore’), *egwan* ‘very weak’ < * *ek-* + * *u̯anno-* ( *gwan* ‘weak’), as well as the evidently late neologisms *egwyl* ‘respite’ (16 ^th^–17 ^th^ century, beside *gŵyl* ‘holiday’) and *egwal* ‘cabin’ (18 ^th^-century, beside *gwâl* ‘lair, den’); furthermore *eban* ‘feeble’ beside *ban* ‘top’ and *edif* ‘greedy’ beside *difiog* ‘voracious’ ( Russell, 1988: 120). At first glance, the first of these appear to contradict the claim above that * *ek-g*° became * *egg*° and ultimately * *eχ*°. However, since these formations are late and secondary, various analogical steps are involved. *Eglwg* ‘conspicuous’ was synchronically analysable as consisting of *eg-* + a lenited allomorph of * *llwg* ‘light, visibility’. *Eglan etc*. can therefore have been formed as compounds of the same *eg-* + *-lan*, the lenited allomorph of *glan*, *etc*. Since, on the other hand, the relationship of the simplex *glan* to the compound *eglan* could also be analysed as that of a prefix * *e-* + unmutated base *glan*, the way was then free to form *e-ban* from *ban* and *e-dif* from *dif*°.

The situation is very different in Gaulish. In the certain instances of the preverb * *ad-* followed by * *b-* or * *g-*, assimilation is typically lacking, *e.g.*, *adgarios*, *adgariontas*, *adgarie* < * *ad-gar-* (contrast OIr. *acrae* ‘act of suing, prosecution’, *·acair* ‘to sue, prosecute; bewitch’ < * *aggar-* of the same structure), *Adbugiounus* < * *ad-bug-*, *Adbogius* < * *ad-bog-* (contrast OIr. *apach* ‘corpse, remains’ < * *abbou̯g-*, possibly of the same structure). Cisalpine Gaulish shows the same treatment if *aśkoneti(o)* represents the name written *Adgonnetius* < * *ad-gonn-et-* in the Latin script. Assimilation is likewise missing between *d* and *m*, as the name *Admina* ( *aśmina* in the Lepontic script) attests. The name *Annamat(i)us* and the placename *Annamatia* are undoubtedly to be connected with the name that is more commonly written *Adnamat°* ‘against the enemies’, but note that attestations for the assimilated variants do not come from the Gaulish heartland, but from ‘marginal’ areas such as Noricum or Pannonia where influence from other languages is conceivable. De Bernardo Stempel ( 2010: 69) cites the divine epithet *Agganaicus* < * *ad-gen-aki̯o-* of Jupiter (Pavia, 2 ^nd^ c. a.d.) as an example for the assimilation of *dg* > *gg*. Perhaps this reflects a late development in Cisalpine Gaulish, but the number of additional changes required for the etymology casts doubt on the relevance of this example. The preponderant lack of assimilation in most of the Gaulish evidence means either that assimilation of voiced clusters across the morpheme boundary was not a pan-Celtic phenomenon, or that these compounds were morphophonologically so transparent that the assimilation could be easily undone by reanalysis.

Its ambiguous writing system renders the pertinent Celtiberian evidence meagre and difficult to interpret. The gentilic name *abo[..]kum*, which has been emended to *aboiokum* on the basis of *Abboiocum* in a Latin inscription, has been explained as * *ad-bog-i̯o-ko-* by Prósper ( 2005: 252‒254). The verbal form *usabituz*, perhaps ‘let him cut out’, may continue * *uts-ad-bi-tūd* ( Schumacher, 2004: 226‒231). While the deficiency of the Celtiberian script with regard to writing obstruent clusters leaves it undecidable if * *d* has been assimilated to * *b* in these cases or if it is just not graphemically expressed, the spelling *Abboiocum* in the Latin script indicates a genuine geminate resulting from assimilation, provided the etymology is correct.

The examples above are all taken from compounds where the identity of the involved elements is well established and would also have been easily recoverable for native speakers. This transparency of the elements may have entailed a special, analogical treatment across the morpheme boundary. It is therefore possible that geminates that are not transparently analysable as resulting from assimilation may show different outcomes. This is conceivably the case in the British Celtic languages, as Anders Jørgensen (personal communication) reminds me. As argued in the sections above, sometimes what must have been voiced geminates in prehistory are reflected by unlenited sounds, sometimes the outcome appears to be spirant sounds, at least in the case of * *gg*, as if an intermediate stage had consisted of a voiceless geminate (cf. Russell, 1988: 115‒125). W *achlan* and *eglan* show contradictory behaviour within a single language. The conditions for the divergent treatment are not always obvious. When the outcome of the geminate in Welsh is a single voiced stop, but lacks parallels in Breton and Cornish, it may have been formed more recently than words with parallels, where the outcome is a voiceless fricative.

At this point it is apposite to look at the treatment of other possible examples of voiced geminates, especially of * *gg*, in the British languages, when they do not occur across transparent morpheme boundaries. They allow placing the ambiguity of the treatment of * *gg* in a bigger picture.

(1) The change * *gg* > * *χ* may have a parallel in PC * *biggo-* ‘small’ > W *bychan*, OCorn. *boghan*, Bret. *bihan*; the reconstruction with * *gg* is necessitated by OIr. *bec*. The unsatisfactory alternative is to assume two different formations * *biggo-* and * *bikk(an)o-* for the two branches.

(2) The case of the word for ‘bell’ is uncertain. OIr. *cloc* presupposes * *kloggo-*. W *cloch*, Corn. *clogh*, Bret. *kloc’h* could either continue that same preform, or they could be borrowed from MLat. *clocca*, which has a geminate * *kk* (cf. also Fr. *cloch*, Germ. *Glocke*). The origin of this word is clearly sound-symbolic (see section 11.2.).

(3) Another item is equally ambiguous: W *gwraig*, OCorn. *grueg*, Bret. *gwreg* ‘woman’ speak in favour of a preform * *u̯rakī* or * *u̯rakū* with *k*. On the other hand, the spelling variants of the rare Irish word *frac*, *fracc*, *frag* ‘woman’ are most straightforwardly interpreted to stand for /g/ < * *u̯raggā-* ‘(old) woman’. This interpretation finds support in Scottish Gaelic *fràg* ‘a kind woman’. While the spellings *frac* and *fracc* could conceivably stand for /frak/ < * *u̯rakkā-*, the spelling *frag* and ScGael. *fràg* would remain isolated in that case. A uniform explanation that accounts for all Gaelic forms is preferable. In addition, the British Celtic languages possess the bye-form W *gwrach*, OCorn. *gurah*, Bret. *gwrac’h* ‘old woman’. This could either reflect * *u̯rakkā* with ‘expressive’ gemination of the * *k* of * *u̯rakī/ū* or, if my analysis above of *bychan etc*. as * *biggo-* is accepted, it could reflect * *u̯raggā-* and correspond directly to the Irish form. Phonaesthetically, *-ch* /x/ acquired a negative connotation especially in Welsh ( Rodway, 2019; Wmffre, 2007: 59; Zimmer, 2000: 278). This may have been prompted by loans from Irish with their frequent suffix- *ach* that conferred a particularly alien and in consequence pejorative feeling on such words ( Sims-Williams, 2011: 183–184). This has been suggested to explain the choice of the ‘phonemaestheme’ *-ch* and the semantics of *gwrach*. However, while this phonetic attitude is specifically Welsh, the negative connotations of *gwrach* appear to go back already to Proto-British, since it is equally attested in all three British languages. This detracts from the phonaesthetic explanation of *gwrach*.

(4) A few other examples are even less certain. The Welsh hapax sg. *gwre* ‘worm, insect, mite’ < PC * *u̯rigā* < * *ṷr̥g⁽ʰ⁾eh₂* stands in a suppletive relationship with pl. *gwraint* ‘mites, worms’, cf. OIr. *frige*, pl. *frigit* ‘vermin, flesh-worm’, Gallo-Lat. *brigantes* ‘worms in the eyelid’ < PC * *u̯rigant-*. Breton has a related word ending in *-c’h*, namely sg. *gwrec’h*, pl. *grec’hent* ‘mite’. The relationship between the Welsh and Breton words is reminiscent of that of W *lle*, OCorn. *le*, OBret. *le* ‘place’ (< * *legā*? cf. Schrijver, 1995: 308) and OBret. *legh*, Bret. *lec’h* ‘place’. Several explanations are possible. The forms with the fricative could continue a sporadically geminated * *-gg-*; or they could continue by-forms with suffixal * *-s-* of unclear function added to the stem, *e.g.*, * *u̯riχsā* and * *leχsā*. Finally, the *-c’h* of Breton could be due to a phonetic strengthening in sandhi of final * *-γ* > * *-χ* in Late Proto-British * *g ^u̯^reγ* and * *leγ*, comparable to the strengthening of * *-h* > * *-χ* seen in Bret. *dec’h* ‘yesterday’ < * *deh* < * *γdes(i)*, in contrast to W *doe* ( Schrijver, 1995: 390). The latter appears to be the simplest explanation. It only entails the extra assumption that the stem form of the singular was then also extended to the plural. In consequence, these words are not relevant for the treatment of geminates.

Several items seem to exhibit the simplification of * *dd* > *d*, like across the morpheme boundary seen above.

(5) The best evidence is W *credu*, MCorn. *cresy* (with secondary assibiliation), MBret. *cridiff*, Bret. *krediñ* ‘to believe’, cf. OIr. *creitem* ‘belief’ < PC * *kred-dī-mā* ‘belief’ < PIE * *k̑red dʰeh₁*- ‘to put one’s heart ( *uel sim*.)’, also supported by Lat. *credere*, Vedic *śrad dhā-*, Avestan *zrazdā-* ‘to believe’ (see now Weiss, 2020). In this word, the geminate does not result from composition with a preverb, but the compositional joint between the constituents arguably remained transparent until late in prehistory.

(6) This is more difficult to argue for a number of other words in which * *dd* can be set up for etymological or comparative reasons. For W *rhwd*, OBret. *rod* ‘rust; ‘mud, filth’, and the Cornish placename *Polroad*, *Polrode*, possibly from * *ruddo-* < * *rudzdo-* see section 6.1. above.

(7) Bret. *red* ‘bog myrtle’ is surely cognate with OIr. *rait* ‘id.’, in which case the preform must be * *ro/addi-* or perhaps * *ruddi-* from the same root * *h₁reu̯dʰ* ‘to become red’ as the previous item ( Stifter, 1998: 216‒217).

(8) Another possible instance is the equation OIr. *gat* ‘theft’ and Bret. *gad* ‘hare’, which leads to the reconstruction of the common preform * *gaddo/ā-* (in Stifter, 2021, I linked the two semantically distant words through the popular belief that hares steal milk).

(9) The Latin loan *abbatem* ‘abbot’ is reflected as W, Bret. *abad*, OCorn. *abat*.

While in Irish it is the rule that geminates that resulted from the assimilation of two stops were subsequently simplified to a single stop, the foregoing discussion makes it unavoidable to conclude that geminate clusters were treated differentially in British. * *bb* and * *dd* seem to have been reduced to single voiced stops, but * *gg* may have become the voiceless fricative * *χ*. Where, on the other hand, single * *g* results in Welsh, this may rather be due to a late analogy. Clearly, more research needs to be done on the treatment of geminates, and it cannot be guaranteed that all relevant examples were taken into consideration in this study, since no exhaustive search was carried out.

### 6.3. *RR

This paragraph brings together a handful of diverse instances in individual languages where geminate resonants do – or do not – arise as a consequence of identical sounds coming into contact across the composition boundary; phenomena which do not fit precisely into any of the preceding sections.

(1) A special, language-internal case of identical sounds across the morpheme boundary is Gaul. *petorritum* ‘four-wheeled wagon’. The geminate *rr* must have arisen from metathesis of earlier * *petru-rito-*, which contains the composition form * *petru-* < * *ku̯etru-* of the numeral ‘4’ (itself metathesised from earlier * *k ^u̯^etur-*), and a nominal formation of the root * *ret-* ‘to run’. Alternatively * *k ^u̯^etur-* could be an archaism preserved in this compound, or *petor-* is simplified from the younger Gaulish form * *petu̯or-* with influence from the cardinal * *k ^u̯^etu̯ores* ‘4’.

(2) A word that is best reconstructed as a compound of the preverb * *kom-* and the root * *men-* ‘to think’ shows the very exceptional behaviour that, instead of expected * *kommen*-(a compound with a root noun?), the lexical stem is reflected as * *komen-* with a simple *m*, namely OIr. *cuman* ‘reminiscent’, W *cof*, MCorn. *cof*, MBret. *couff*, NBret. *koun*, *kouñ* ‘memory’. The behaviour of * *komen-* finds no parallel in other compounds of * *kom-* with a root beginning with *m-*. Instead, gemination is the rule, *e.g.*, OIr. *commailt* ‘consumption’ < * *kom-melti-*, *commus* ‘competence’ < * *kom-med-tu-*, or W *cymaint* ‘as large as’ < * *kom-mantī-*, *etc*. The difference is perhaps one of time-depth (VKG i 171; Jackson, 1953: 481). The simplification of * *mm* > *m* could even be an echo of the Proto-Indo-European constraint on geminates. Alternatively, the allomorph * *ko-* of * *kom-* could have been extrapolated from adjectives such as PC * *ko-u̯ar-i̯o-* < * *kom-u̯ar-i̯o-* ‘proper’ (OIr. *coäir*, MW *cyweir*) or * *ko-u̯īr-V-* < * *kom-u̯īr-V-* ‘correct’ (W *cywir*) where * *m* was regularly lost before * *u̯*. To make things even more complicated, OBret. *commin*, glossing *annalibus* ‘annals’, must surely reflect the etymon of OIr. *cuman etc*., too. However, it is written with double *mm*, which otherwise represents unlenited *m*, namely in *camm* ‘oblique’, *gueimmonou* ‘seaweed’, *lammam* ‘I jump’, *pimmont*, *pimmunt* ‘50’.

(3) A potential parallel is OIr. *neim* ‘poison’, which inflects as a neuter *n*-stem in the singular. The underlying formation would be expected to be * *nem-men-* (root * *nem-* ‘to apportion’ + suffix * *-men-*), but since the Old Irish word descriptively continues * *nem-en-*, it is attractive to operate with the same early simplification of * *mm* here. A different possibility for both * *kommen-* and * *nemmen-* is to assume the loss of the medial consonants in cases where the second syllable came to stand in the zero grade, *i.e.*, * *kommn-* > * *komn-*, with subsequent generalisation of the simplified stem * *komen-* (personal communication Michael Weiss). Such a strategy has been employed to explain OIr. *gein* ‘birth’ < * *genen-* ← * *g̑enh₁men-* (see 5.1. (1)).

At the end of the foregoing developments, the phonological system of Celtic had morphed into the one in Table 2.

**Table 2.** the early Common Celtic sound system.

	plosive	nasal	fricative	glide	liquid
bilabial	b (bː)	m mː	(φ)	w	
dental	t (tː) d (dː)	n nː			l l: r r:
alveolar			s (z) tˢ		
palatal				j	
velar	k (kː) g (gː)		(x ɣ)		
labiovelar	kʷ (kʷː?) g>ʷ (gʷː?)				

There was a contrast between *s* and a strong sibilant, although that contrast does not mirror that of simple *vs*. geminate consonants elsewhere. It is probable that single voiced stops already had lenited allophones in intervocalic position, but this is not indicated in the system above. On the whole, geminated voiced stops were rare except across the morpheme boundary (an observation already made by Martinet, 1952: 198).

## 7. Etymological gemination: Proto-Goidelic * *NT* > * *DD*

Notwithstanding the many diverse developments presented so far, one of the most common sources for geminate voiced stops in Irish are Proto-Celtic clusters of nasal + voiceless stop that developed into the corresponding voiced geminates after the separation of Goidelic from the rest of the Celtic languages, *e.g.*, Proto-Goidelic * *gg* < PC * *nk*. At some point in the prehistory of Irish, the voiceless stops in such clusters assimilated in voice to the preceding elements, while the nasals assimilated in the mode of articulation to the following stops (GOI 126‒127; McCone, 1996: 106‒109; Schrijver, 1993: 35‒39). The two met, as it were, in the middle and a geminate voiced obstruent resulted. While geminate voiced stops are a rarity in Proto-Celtic and in other ancient European languages ( Martinet, 1952: 198), thanks to this change they are very common in Primitive Irish.

In ogam inscriptions of the classical period (5 ^th^–7 ^th^ century a.d.), the resulting voiced stops are written with the letters for D and G, *e.g.*, DECCEDDA < PC * *dekantos* or TOGITTACC < * *tonketāko* (the double spelling DD or of the other consonants has nothing to do with geminate sounds, but is an orthographic convention in ogam that is independent of the phonological nature of the consonant). In Old Irish orthography, they are usually expressed by ⟨c⟩ and ⟨t⟩ (and by ⟨p⟩ for /b/ arising in other contexts). Preceding Proto-Celtic short * *a* and * *e* (the latter of which had been raised allophonically to mid-high * *ı* before the tautosyllabic nasal) were lengthened in the process and fell together as *é*. They are retained as such in accented syllables, but are shortened along with other long vowels in unaccented syllables. As a consequence, words with initial *éc-* and *ét-* very commonly go back to compounds consisting of the negative prefix * *an-* ‘un-’ + etyma in *c-* and *t-*. There are countless, uncontroversial examples for this chain of sound changes. To name just a few representative examples:

(1) PIE * *h₁dn̥t-* > * *dant-* > OIr. *dét*, W, Bret. *dant*, Corn. *dans* ‘tooth’.

(2) PC * *sentu-* > OIr. *sét*, Gaul. *sentu-*, W *hynt*, OBret. *hint*, Bret. *hent*, Corn. *hens* ‘path’.

The unstressed development of the vowel is exemplified by

(3) PIE * *h₂r̥g̑n̥to-* ‘shining one’ > * *arganto-* > OIr. *argat*, Gaul. *arganto-*, Cib. *argato-*, W *arian*, *ariant*, OCorn. *argans*, MCorn. *arhans*, OBret. *argant*, Bret. *argant* ‘silver, money’.

(4) and by the Old Irish preverb *ceta·* < * *kanta* < * *km̥th₂* ‘together with’ (cf. OW *cant* ‘with’) and by the preposition/preverb *etar* < * *enter* ‘between’.

Preceding * *o* and * *u* were not lengthened, *e.g.*, * *tonketo-* > ogam TOGITTACC, OIr. *tocad* ‘fortune’, * *kon-toletu-* > OIr. *cotlud* ‘act of sleeping’, or * *slunke-* > OIr. *slucaid/sluicid* ‘to swallow’ ( Schumacher, 2004: 593). The evidence for * *i* ‒ in contrast to mid-high * *ı*, the allophon of * *e* before a tautosyllabic nasal ‒ has been ambivalently assessed, and it warrants more detailed attention. The following paragraphs are therefore a digression from the overall topic of geminates.

McCone ( 1996: 107‒108) cites *ro·ic* ‘to reach’ < * *inket* ← * *īnkti* < * *h₂ēnk̑ti* and *fet* ‘whistle’, with regular lowering from * *u̯into-* ‘wind’ < * *u̯īnto-* < * *h₂u̯eh₁n̥to-* ‘blowing’ as relevant evidence for the question of the treatment of * *iNC*. Rix makes the tentative alternative, but phonologically ultimately equivalent suggestion of analysing *ro·ic* as a continuation of * *īnk-* from a reduplicated present * *h₂i-h₂n̥k̑-* (LIV 282‒283, fn. 7). This position implies that * *ink* became intermediate * *igg* with a short vowel.

This approach is gainsaid by Schrijver ( 1993: 39‒42) and Schumacher ( 2004: 200‒204) who explain *ro·ic* as * *ınket* < * *ænnket* < * *annketi* ← * *h₂n̥nkti*; *fet* can be explained semantically more easily as * *u̯izdo-* from the root * *(s)u̯ei̯zd-* ‘to whistle’. On the other hand, Schrijver and Schumacher derive *léicid* straightforwardly from * *link ^u̯^i̯eti* ← * *li-n-k ^u̯^-*, while McCone ( 1998: 474‒475) has to argue for analogical influence from a long-vowel form of the subjunctive stem at a pre-Irish stage.

Other forms have received less attention in this dispute, even though they are equally relevant for determining the outcome of PC * *inC*. McCone ( 1996: 107‒108) mentions a couple of “problematic instances with unlengthened stressed vowel”, namely *ecor* ‘arrangement’ (the verbal noun of *in·cuirethar*), *tecosc* ‘instruction’, *do·ecmaing* ‘to befall’, *do·ecmalla* ‘to collect’, and conjugated and therefore stressed forms of the preposition *etar* ‘between’, such as *etruinn* < * *enter-snī* ( *uel sim*.). McCone wonders if “the following *o* (plus *r* or *m*/ *ṽ*) played a role in the loss of the nasal”, but he arrives at no other conclusion than that “the precise conditioning remains unclear”. He refers to GOI 518‒519 in this context, but does not actually quote or discuss Thurneysen’s alternative solution, even though it merits a closer look.

Thurneysen proposes that “these examples can best be explained by assuming that in them the preposition had at one time the form *in-*” (relevant forms are also discussed by Armstrong, 1976: esp. 64‒66). The Celtic preposition with the meaning ‘in’ had been inherited from Indo-European as * *eni* ( Dunkel, 2014: 224‒225). It is attested as a plain preposition in the most archaic form in Celtiberian *eni*, and in a few nominal compounds in other Celtic languages, *e.g.*, Gaul. *Enignus*, OIr. *ingen* ‘daughter’, Ogam INIGENA < * *enigenā*, OIr. *inis*, W *ynys*, Bret. *enez* ‘island’ < * *eni-sth₂-ih₂-*. However, in the Gaulish preposition *in* it appears with loss of final *-i* and with unexpected raising. This same form * *in* underlies also the ordinary Old Irish preposition *i ^N^* and Welsh *yn*. That the Irish preposition continues the high vowel * *i* follows from the fact that * *en* with mere apocope of final * *-i* would have resulted in ** *a ^N^* in Old Irish, like the masculine infixed pronoun *-a ^N^* < * *-en* did. Dunkel ( 2014: 223) suggests that the vowel of Gaul. *in* reflects the raised allophone * *ɪ* of * *e* before a nasal in tautosyllabic position, *i.e.*, before consonant. ^3^ In Irish, * *ɪn* is usually kept distinct from original * *in*, but in this case it has to be assumed that mid-high * *ɪ* was further raised to * *i*.

In Gaulish, this allomorph also intruded into compounds, *e.g.*, *Indercillus* < * *en(i)-derk-* ‘having an eye inside (?)’ or *in-dutio-* ‘being endowed with (?)’ in the name *Indutiomarus*, perhaps from * *en(i)-dʰoh₁t-* ( Delamarre, 2003: 163). In Irish, * *in* was semantically and morphologically conflated with Proto-Celtic * *andi/ande* < * *n̥dʰi*. ^4^ This is most readily seen in the preposition itself: while the plain preposition is *i ^N^* < * *in*, the inflected forms such as 1sg. *indium*, 3pl. *intiu etc*. are built on the stem *ind-* < * *ande*. In the 3sg. masc. *and*, yet another etymon * *andom* < * *n̥-dom* ‘at home, inside’, was drawn into the paradigm ( Dunkel, 2014: 159, 230).

In verbal composition, the situation is even more complex. The allomorphs of the preverbs have been conflated to such a degree that a clear distinction is not always possible. This is not aided by the fact that the presentation and morphological analysis of verbal forms in eDIL and other handbooks is often imprecise or incorrect. The archaic allomorph * *eni-* shines through OIr. *do·infet* ‘to inspire’ and its 3sg. present subjunctive · *tinib* < * *to-eni-su̯izd-*, but otherwise evidence for it is hard to come by and hard to distinguish from the more common allomorphs * *ande-* and * *in-* (reconstructed preverbs are written with a final hyphen in order to distinguish them formally from prepositions and adverbs). The 2sg. imperative of *in·cosaig*/ *in·coisig* ‘to signify, indicate’ is *inchoisc*. As is evident from the lenited *-ch-*, this cannot be from * *in*/ *en-kom-sech-* but must either go back to * *enikom-sk-* or * *ande-kom-sk-*. eDIL quotes no attestations with *-d-*, *e.g.*, * *·indchoisc*. The lack of such forms in a verb that is well attested in early sources can be taken as indirect evidence that it involves * *eni-*.

The most common allomorph in verbal compounds appears to be *ande-. It seems that in the earliest period it occurred as *ind·* in pretonic position and as · *in(d)-* in stressed position, as opposed to * *in-* that appeared as *in·* and · *in-* respectively. Ultimately both merged in *in·* in pretonic position and cannot always be kept apart in other positions either. A precise description of all contexts is not intended here. Finally, and to complicate matters even further, it will become clear from the following that beside * *eni-*, * *ande-* and * *in-*, there was also a fourth allomorph * *en-* with a much more restricted domain. Leaving aside * *eni-*, whose presence in a handful of Irish compounds was demonstrated above, it can be shown by minimal pairs of phonological micro-contexts that a three-way contrast between * *ande-*, * *in-* and * *en-* needs to be made. The distinction between * *ande-* and * *in-* is required to account, for instance, for the different behaviour of *in(d)·lá* ‘to enter into, arrange, *etc*.’ and its prototonic stem, represented by the 3sg. subj. pres. · *indell* < * *ande-la-* (not **· *ell* < ** *·in-la- uel sim*.), versus *in·loing* ‘to join, bring together, put upon, *etc*.’ and its prototonic form · *ellaing* < * *in-long-* (not **· *in(d)laing*); or *in·samlathar*, *·intamlathar* ‘to imitate’ < * *ande-saml-*, versus *in·snaid* ‘to insert’, verbal noun *essnaid* < * *in-snad-* (not ** *intnaid*).

The reason why * *in-* instead of * *en-* is set up here for the preforms, at least for deuterotonic verbal forms when the preverb stands before the accent, is that * *en-* in unstressed position would have become ** *an·*. ^5^ On the other hand, the presence of the allomorph * *en-* (or potentially even * *an-*!) is required in other contexts, for instance by verbs such as *con·éitet* ‘to accompany’ < * *kom-en-tei̯g-*; or *in·túaisi* ‘to listen to’ whose prototonic stem must be from * *en-tou̯stī-*, *e.g.*, 3pl. *·éitset*; or by the augmented subjunctive stem of *in(d)·fét* ‘to tell, relate’, *e.g.*, 1sg. pres. subj. *·écius* < * *en-kom-u̯ei̯d-s-*. All of these verbal forms show the regular change of * *en-* > *é-* before a Proto-Celtic voiceless stop described at the beginning of this chapter. In the case of *·ellaing*, no decision can be made between * *in-* and * *en-*, since the outcome would be the same. Ultimately, in this way a fourfold allomorphy of * *eni-*, * *en-*, * *in-* and * *ande-* can be demonstrated in verbal morphology, with a number of distributional restrictions on their occurrence, as illustrated in Table 3.

**Table 3.** the four allomorphs of preverbal ‘in’ in Primitive Irish.

	reconstruction	preverb in pretonic position	preverb in tonic position
1.	*to-eni-su̯izd-	*-*	*do·infet, ·tinib*
	*eni-kom-sech-	*in·cosaig, in·coisig*	*inchoisc*
2.	*ande-la-	*in(d)·lá *	*·indell*
	*ande-samlā-	*in·samlathar *	*·intamlathar *
3.	*in/en-long-	*in·loing (in-)*	*·ellaing (in/en-)*
	*in/en-snad-	*in·snaid (in-)*	*VN essnaid (in-)*
	*in/en-tou̯stī-	*in·túaisi (in-)*	*·éitset (en-)*
4.	*kom-en-tei̯g-	*-*	*con·éitig*
	*en-kom-u̯ei̯d-s-	*-*	*·écius*

While all the phonological developments invoked so far are trivial in the diachrony of Old Irish, this is not the case for the words quoted by Thurneysen (GOI 518‒519) and McCone ( 1996: 108‒109), namely *ecor* ‘arrangement’, *tecosc* ‘instruction’, *do·ecmaing* ‘to befall’, *do·ecmalla* ‘to collect’ and *etruinn*. These cannot go back to * *en-koro- etc*. since it was just seen that this would have regularly resulted in ** *écor etc*. The logical conclusion, after the alternatives have been excluded, is that they must continue * *in-koro-*, ^6^ * *to-in-kom-sk ^u̯^o-*, * *to-in-kom-ink-*, * *to-in-kom-la-*, and * *inter-snī* respectively, with the development of * *in-k-* > * *igg-* in a first step, and then with regular lowering of * *igg-* before a back vowel > * *egg-*. The same solution applies to *itge*, *itche* ‘request, petition’ < * *in-tech-i̯o-* < * *in-tek ^u̯^-i̯o-*, only with the regular absence of lowering. ^7^

What about the distribution of * *in- vs*. * *en-*? Perhaps its obscured rationale is that * *in-* was originally at home in pretonic position, that is, as a plain preposition and before the accented part of the verbal complex, while * *en-* occupied the stressed part of the verbal complex, as reflected in *con·éitet*, *·éitset* or · *écius*, and was used in most verbal nouns. In a subsequent development, the unstressed variant intruded by analogy into the stressed portion of the verb, which explains cases such as *do·ecmaing* or *ecor*. The replacement of * *en-* by * *in-* in this environment is not a regular, but a sporadic, analogical process. Operating with a sporadic replacement is not completely arbitrary or random. That there could be a formal distinction between pretonic and tonic allomorphs of preverbs is commonplace elsewhere in Old Irish, for instance in the alternation *do· vs ·to-* or *a ^H^*/ *as· vs*. *e(s)-*. It is equally well known that the two allomorphs could influence each other. In the case of *a ^H^*/ *as·*/ *e(s)-*, the spread of the pretonic allomorph into tonic position is observable in such verbs as *as·beir*, · *epir*, where the imperative is found as *apair* already in Old Irish, or in pronominal forms such as 1sg. *asum* or 3sg. masc./neut. *as* instead of archaic *es-*. In the case of *do·/to-*, the tonic allomorph was frequently used in pretonic position, in archaic and in archaising orthography, for example archaising *to·beir* for *do·beir*.

The presence of two allomorphs side by side with each other in one paradigm is not isolated in the wider Celtic perspective either. It is securely attested for the prehistory of the Old Irish preposition *do* ‘to, for’ and for the preposition/preverb *de* ‘from’, the distribution of neither of which follows predictable rules throughout their respective paradigms. In the case of *do*, the allomorph PC * *dū* < PIE * *doh₁* ( Dunkel, 2014: 148‒149) underlies the plain preposition and conjugated forms such as *dúinn* ‘to us’ < * *dū-snī(s)* or *dúib* ‘to ye’ < * *dū-su̯ī(s)*, as well as British Celtic * *dī* and perhaps Gaulish *duci*, whereas * *do* < PIE * *do* underlies conjugated forms such as *duit* ‘to you’ << * *do-tī* < * *do-toi̯* or *dó* ‘to him/it’ < adverbial * *do*. In the case of *de*, its allomorphs PC * *dī* < pre-Celtic * *dē* and * *de* < * *de* occur without apparent distributional rationale in several compound verbs, *e.g.*, *·díltai* ‘to deny’ < * *dī-slondī- vs*. *dermat* ‘forgetting’ < * *de-ro-mento-* (but see the critical discussion in Dunkel, 2014: 148‒156).

A side remark: The preposition/preverb PC * *enter* is always continued with a short vowel in OIr. *etar* (cf. Lash, 2017 for other Old Irish allomorphs of it). This is expected for the preposition in pretonic position, but the short vowel is not expected in inflected forms of the preposition and when the preverb occurs in nominal compounds. For instance, the 1pl. is attested as *etruinn*, not as expected ** *étruinn*, or the word for ‘boundary-ditch, fence’ is *etarbae*, not expected ** *étarbae*. It might look attractive to derive those forms from an innovatory allomorph ** *inter*, in which the more basic local preposition * *in* had analogically caused the replacement of inherited * *en* by * *in*. However, such a scenario is excluded. There is no possibility how the hypothetical preform * *inter would have led to *ed’er with lowered * *e-* in the first syllable. It is therefore more economic to assume that the preform is indeed the traditional *enter > *ēd’er, ^8^ which *via* vowel shortening and depalatalisation in pretonic position resulted in *eder. In a further step, this shortened pretonic variant replaced the original tonic variant * *éter* everywhere. The complete replacement across the board of all tonic allomorphs by the pretonic ones in this scenario nicely illustrates the randomness of analogical change. In the case of several other prepositions, the language tolerated the coexistence of dual stems in unstressed and stressed prepositions: *co vs*. *cuc-*, *amal vs*. *saml *, *dar vs*. *tor-* or, indeed, as demonstrated above, *i vs*. *ind-*/ *and*.

## 8. Etymological gemination: Insular Celtic mutations

When we broaden the perspective from the level of isolated lexemes to that of accentual units and constituent phrases, we can see that some of the sandhi-effects across words that ultimately became the initial mutations of the Insular Celtic languages, can be analysed as the same or similar types of geminations that arose word-internally. These sandhi-related processes, namely the interaction of word-final sounds with word-initial sounds, thus extended the positions where geminate sounds were phonotactically permissible from word-internal to word-initial position. To all extents and purposes, internal and external sandhi behave equivalently. A comprehensive diachronic description of the emergence of mutation in Insular Celtic is outside the aims of this study. The present section only aims at highlighting the fundamental parallels of certain types of mutations with word-internal gemination. Only sandhi involving two interacting consonants is relevant for the question of gemination. Leniting contexts, where an initial consonant originally followed a word ending in a vowel, are therefore ignored here.

### 8.1. Irish

In the following, the examples will be drawn mainly from contexts that underlie mutations in Irish. Traditional grammars of Irish mention a mutation which they call ‘gemination’ ( *e.g.*, GOI 150–153). This is a misnomer, both synchronically and diachronically. Diachronically, other sandhi contexts also led to geminate sounds in Primitive Irish in the appropriate contexts. Synchronically, the occasional geminate spellings of certain sounds are best regarded as markers of non-lenition ( Greene, 1956). Instead, the main effect of this mutation, which finds no graphic expression in genuinely Old Irish sources, but which is directly observable in Middle and Modern Irish written sources and in Modern Irish pronunciation, is the prefixation of *h-* to a following word if it starts with a vowel. Therefore this mutation is called ‘aspiration’ here (cf. Stifter, 2009: 65).

The sandhi contexts that are relevant for the diachronic study of gemination are therefore nasalisation, aspiration, and non-mutation, *i.e.*, the appearance of the unmutated radical sound. It is essential to acknowledge that in addition to the morphosyntactically recognised mutations, the absence of an overt change, *i.e.*, ‘non-mutation’, also is a mutation in its own right. From the point of view of diachronic phonology, aspiration and non-mutation are for the most part just two sides of the same coin. In the vast majority of cases, both continue contexts where the mutated word was preceded by a word ending in *-s*. ^9^ At the time when final syllables had not been apocopated yet, the effects of non-mutation must have been identical to those of aspiration. The split between aspiration and non-mutation depends on the context: either intervocalic or adjacent to a consonant. Aspiration occurs when the final * *-s* > * *-h* combined with the initial vowel of the following word in Primitive Irish after the shift of the syllable boundary. When a consonant followed, the *-h* merged with that consonant. In most cases, this must have led to a phonetically slightly longer pronunciation that, at least in the case of the resonants, but perhaps also of stops, meant phonetic similarity to or identity with word-internal geminates. However, the effect was different if the following word started with * *u̯-*. In that case the chain of events was * *-s u̯-* > * *-h u̯-* > *# *hu̯-* > OIr. *-Ø f-*, where the outcome *f* (phonologically equivalent to a geminated * *u̯*!) is identical to that of the internal group * *-su̯-*, but is not phonetically similar to single * *u̯*, which rather gets lost. On the level of surface phonetics this means that when * *-h* stood immediately before a consonant, it was absorbed by it and non-mutation ensued.

Synchronically, aspiration is only caused by proclitic mono- or disyllabic particles that end in a vowel. It is conceivable that aspiration was originally also caused by Old Irish inflectional forms that ended in a vowel, for example the accusative plural of masculine and feminine inflectional classes, but there is no written evidence for this. This effect can only be conjectured. Non-mutation, in any case, occurs after all classes of words that ended in a consonant and in phrase-initial position.

Through the outlined processes, non-lenited initials and non-nasalised initial voiced stops came to be phonetically identifiable with internal geminates. The phonetic salience of geminates word-internally, and the rise of geminates word-initially, must have mutually reinforced each other. Sandhi-generated geminates will have considerably increased the overall token frequency of gemination in speech and strengthened its phonological status in the system. In contrast, gemination was rare in absolute word-final position.

In the case of the nasal mutation, several contexts need to be distinguished. A probably weakly articulated nasal sound in final position was attached and merged with initial consonants. It assimilated fully to initial resonants. That this sound must have been phonologically * *-n* can be gleaned from the different treatments of inherited initial and internal clusters with * *m*, which do not turn into geminates, *e.g.*, * *mrogi-* > OIr. *mruig* ‘land’, * *mligeti* > *mligid* ‘to milk’, or * *kom-rigo-* > OIr. *cuimrech* ‘binding, bond’. Furthermore, this *-n* is directly visible in the nasal mutation *n-* on initial vowels. It is therefore evident that, like in Gaulish and British Celtic, final PC * *-m* in the ending of accusative singulars, genitive plurals, and neuter nominative singulars, had become * *-n* in the prehistory of Irish. There are not many inherited word-internal clusters of * *n* + * *r* or * *l* that illustrate their identical treatment to that across the word boundary. The best examples are furnished by compounds with the preverb * *en-* ‘in’, where it hadn’t been replaced by other allomorphs. Practically speaking, the only good examples are those with * *l*, which indeed show the expected assimilation to *- *ll-*, namely OIr. *ellam* and *ellach* (see section 3.6.). For * *-nr-*, the only potential example that I am aware of is OIr. *eirr* ‘chariot-fighter’. This has been suggested to continue * *en-ret-* ‘he who runs into (the battle)’ (see NIL 577 n. 3), but the analysis * *ers-sed-* ‘he who sits at the back’ is preferable because of its parallel to *arae* ‘chariot-driver’ < * *are-sed-* ‘he who sits at the front’ (see 3.2. (4)). Nasalisation of voiceless initial stops has exactly the same outcome as in word-internal position, *i.e.*, a voiced single stop results synchronically. It can be surmised that the sound was a geminate at an intermediate stage (cf. chapter 7.). Nasalisation of voiced initial stops does not lead to geminates, but to prenasalised stops.

### 8.2. British and Gaulish

In contrast to the situation outlined for Irish, there is a broad consensus that gemination of initial sounds played no role in the emergence of British Celtic mutations ( Harvey, 1984; Russell, 1985; Schrijver, 1999; Sims-Williams, 1990; Sims-Williams, 2008; Thomas, 1990).

Very occasionally, external sandhi phenomena that look similar in structure to Insular Celtic mutations can be found written in Gaulish inscriptions, *e.g.*, *reguccambion* < * *regū-k ^(u̯)^’ kambion* ‘and I straighten the crooked’ in the inscription from Chamalières (L-100). The final *c* of *reguc* is probably enclitic * *-k ^u̯^e* for ‘and’ that merged with the initial *c-* of *cambion* ‘crooked’ (differently De Bernardo Stempel, 2010: 69).

## 9. Non-etymological gemination: onomastic gemination

### 9.1. Vocative morphophonology

While phonological developments represent the largest source for geminates, other factors may also have contributed to the increase of geminate sounds in the language. One such factor is the pragmatics of onomastic morphology. It is a general observation that, especially in the ancient Celtic data, gemination is especially frequent in anthroponomastics, but not among the ‘long’ dithematic compound names of the Indo-European type, which often have martial and heroic connotations, but rather among more colloquial short or hypocoristic names ( Stüber *et al*., 2009: 37–38). This morphophonological behaviour is not limited to Celtic, but is a feature of many ancient Indo-European naming systems (cf. Schmitt, 1995: 425, 618, 620; and Ellis Evans, 1967: 296–297, 376 with earlier literature). Masson ( 1986: 220) stresses the central pragmatic importance of the vocative as the context in which gemination of stem-final consonants could arise, in personal names but also in expressions that belong to colloquial registers.

The origin of this process of onomastic gemination may lie in ‘vocative reduction’ or ‘vocative truncation’, one method of the formal marking of vocatives (see Daniel & Spencer, 2009: 628–629 for this and other types of vocative marking). Descriptively speaking, vocatives can be shorter than the forms of names that fill the regular argument slots in a sentence (cf. the pertinent examples cited in Daniel & Spencer, 2009: 629; Janson, 2013: 224–231; Schmitt, 1995: 419–425, 618). Indo-European languages bear this out in many ways. The shortening can take the form of the reduction of the number of segments or of the reduction of moras, for example in the Proto-Indo-European vocative of *o*-stems such as * *u̯iHros* → * *u̯iHre* ‘man’; or the vocative of *-eh₂-*stems, caused by laryngeal loss in pausa, *e.g.*, qPIE * *g ^u̯^enah₂* → * *g ^u̯^ena* ‘woman’, *e.g.*, in OCS voc. *ženo*; for consonant stems, cf. Greek nom. Σωκράτης ( *Sōkrátēs*) → voc. Σώκρατες ( *Sṓkrates*), which shows both stress shift and mora reduction.

In other languages, whole syllables are dropped at the end. A contemporary illustration is the neo-vocative of Russian whereby male names in *-a* lose the final vowel, *e.g.*, nom. *Saša* → voc. *Saš*. This habit has recently been borrowed into Georgian ( Amiridze, 2022: 1–2). At the same time, this pragmatic shortening of names in situations of address – no doubt connected with the emotional urgency typical of conversations – can be counteracted on other levels of the speech act. The reduction or loss of segments, especially at the very end, is sometimes balanced by increasing the moraic count further to the front of the word. This is illustrated by the Georgian neo-vocative, where the loss of the final vowel is accompanied by the lengthening of the root vowel, *e.g.*, *Šota* → voc. *Šoot*, or *Gvanca* → voc. *Gvaanc* ( Amiridze, 2022: 2), so that the overall moraic count of the name remains the same. Vowel lengthening is a typologically common process of vocative marking ( Daniel & Spencer, 2009: 629). I believe that consonant gemination is a comparable phenomenon that is equally linked to vocative truncation, with the difference that, instead of lengthening a vowel as in Georgian, it is accompanied by the lengthening of the final consonant. Gemination can thus be regarded as a process that compensates for phonological or morphological loss, perhaps as a way of making up towards the end of the utterance for the overall reduction in the moraic structure. Due to the absence of suitable textual genres in the surviving documentation of the ancient Celtic languages, the connection between gemination and vocatives cannot be demonstrated with actual examples, but the relatively high number of geminates in personal names is indirect evidence of this tendency.

### 9.2. Gemination in Ancient Celtic names

Once gemination had been established as a morphological feature of personal names in a highly specific context, namely in the address of persons in intimate or informal speech acts, it could then be transferred also to contexts outside of vocatival function (for the generalisation of vocatives to other contexts more broadly, see Stifter, 2013). Examples of shortened names with gemination in core syntactical functions are well attested in ancient Celtic, *e.g.*, Gaul. *Eppo*, reflecting a compound name with * *epo-* ‘horse’ as first member, *Blattia* ← * *blātu-* ‘flower’, or *Sammus* ← * *samo-* ‘summer’. Commonly the ‘root’ (or rather onomastic basis) of such names is monosyllabic. Sometimes the etymology of Gaulish names is not entirely certain. For instance, the Gaulish name element *poppo-* has been interpreted as ‘cook’ < PIE * *k ^u̯^ok ^u̯^o-* ( Delamarre, 2003: 252), and the names *Peccia*, *Peccio* have been compared with Ogam Irish QECIA, QECEA < PIE * *k ^u̯^ek⁽ ^u̯^⁾i̯o-* ‘couragious, strong’ ( Delamarre, 2003: 247), related with W *pybyr* ‘eager, vigorous, brave’ < * *k ^u̯^ek ^u̯^ro-*. Onomastic gemination is the best explanation in such cases.

Conceptually related is gemination in kinship terms and in generic nouns for persons that are intimately known, *e.g.*, OIr. *macc* ‘son, boy’ < * *mak ^u̯^k ^u̯^o- vs*. ungeminated * *mak ^u̯^o-* in Gaul. *mapon*, W, Corn., Bret. *mab*; or W *geneth* ‘girl’ < * *genettā- vs*. Gaul. *geneta*, OIr. *geined* < * *genetā-* (more on this in section 11.1. on symbolic gemination). Gaulish has the personal name *Matta*, perhaps created from * *mati/u-* ‘good’, but this word also appears in Raeto-Romance as a generic term for ‘girl’. Maybe it had a similar generic function already in spoken Gaulish, from where it was retained as a substratum loan in Romance.

But also names with a larger phonetic body show gemination. A consonant, usually the final consonant before the ending, can be geminated without concomitant shortening of the name. Suffixes with voiceless velars are common in all Celtic languages. They continue earlier * *-ko-* or * *-k̑o-* added to vocalic stems, whence more complex suffixes such as * *-iko-*, * *-uko-*, * *īko-*, or the very productive * *-āko-* arose in the Celtic languages. In Gallo-Greek inscriptions, *-Vkko-* is occasionally found instead of expected *-Vko-*, especially when the vowel is *-i-*: Δονικκα, Ουαλικκο(νε), Ουηϐρουκκου, and the fragmentary Βιλλικκ], Ερικκ[, ]ουικκοϲ, ]ουλικκι. ^10^ Sometimes there is a geographical bias to such formations. For example, four of the five examples of the name *Maricca*, most likely derived from the adjective * *māro-* ‘big, great’, are found in Noricum and Pannonia. Since there is no phonological factor discernible that could have triggered the gemination in such formations, it is natural to assume that it has to do with the onomastic character of the data.

The comparatively compact Gallo-Greek corpus in *Recueil informatisé des inscriptions gauloises* (RIIG) is an ideal field to study the distribution of geminate sounds in names. RIIG currently consists of 426 inscriptions. Of these, 234 are too short or too fragmentary for a meaningful analysis. The remaining 192 inscriptions contain 65 examples of geminate sounds. Under close inspection, their distribution turns out to be heavily skewed. The following results are based on a small sample and are therefore only preliminary.

As Table 4 shows, some geminates have a clear predilection for suffixes, while others occur only in the root or in the semantically meaningful portion of the names. *-ll-* (whose possible origin as a suffix was discussed in section 2.1.) and *-kk-* are by far the most common geminated stem-final consonants. Of the twelve examples of *-kk-*, nine (75%) occur in suffixes, only three in roots. A similar situation is observable among the seventeen examples of *-ll-*: eleven (65%) are in suffixes, six in roots. The ratio is reversed in the case of the other sounds. All eleven examples of *-nn-* in Greek-letter inscriptions are in roots. Suffixal *-nn-* is only found twice, both in inscriptions in the Latin script. This fact is significant and may have something to do with the rendering of Gaulish suprasegmental phonology in Latin (see section 10.2.). Of the nine examples of *-ss-*, six are in roots and three in suffixes. In the case of * *-tt-* (lumped together with - *θθ-*), there are seven examples in roots and only one in a suffix. Of those seven, five belong to the onomastic stem * *att-*, apparently from the etymon PIE * *atto-* ‘daddy’ (see 1. (1)). Other geminates are too rare to draw clear conclusions. *-mm-* occurs only once in a root, and *-rr-* is found two or three times, apparently always in roots. The two examples of *-pp-* are too damaged to draw any conclusions. The most striking observation, however, is that voiced obstruents do not occur as geminates at all in this corpus. This links in with the overall impression that emerges from this study, namely that geminate voiced stops arose only very late through processes in the individual languages or that they are chiefly found in lexemes that are suspect of borrowing.

**Table 4.** geminates in the Gallo-Greek inscriptions.

	total	stem	suffix	unclear
*ll*	17	6	**11**	
*kk*	12	3	**9**	
*nn*	13	**11**	2	
*ss*	9	**6**	3	
*tt*	8	**7**	1	
*rr*	3	**3**		
*pp*	2			2
*mm*	1	**1**		
*bb*	—			
*gg*	—			
*dd*	—			

On a more general note, mainly based on anecdotal observations, geminates seem to be more common in Gaulish short names or in names formed with suffixes. This may be a special characteristic of Gaulish and seems to be less common in Irish. The distribution of geminate sounds in ancient and medieval Celtic names deserves research on a much wider and more diverse material basis, but this goes beyond the aims of this article. There are some practical methodological limitations to identifying relevant examples in the written record. The Lepontic script of the Cisalpine Celtic languages and the Celtiberian script do not distinguish graphically between single and geminate consonants. Geminates in these languages can only be identified when vernacular names are also transmitted in the Latin or Greek alphabets, either in epigraphy or in manuscript texts. For instance, the Celtiberian name ⟨lubos⟩ in the Celtiberian script has a counterpart in the genitive *Lubbi* in the Latin alphabet, but, to complicate matters, *Lubus* is also found in epigraphy. Cisalpine Celtic *aśkoneti(o)* in vernacular writing has a parallel in *Adgonnetius*, *kasilus* corresponds to *Cassillus*, and *esanekoti* possibly contains the adjective * *kotto-* ‘old’. In other cases, such as *koimila* and *anteśilu*, the lack of a Latin parallel does not permit to decide if the *l* is single or geminate. Furthermore, the frequency of gemination in the attested *written* corpus of ancient and medieval Celtic languages may give a distorted picture. Dithematic names without onomastic gemination, which may be typical of the small aristocratic elite, may be overrepresented in our available sources. It is conceivable that the frequency of short or hypocoristic names with onomastic gemination was higher among the non-aristocratic population and accordingly in everyday spoken language.

While the examples in this section are mostly taken from Gaulish, the formal processes of vocative truncation counteracted by compensatory gemination are valid for all Celtic languages. These processes established gemination as a morphophonological feature in the naming system. Through them, names, especially high-frequency variants such as hypocoristics, came to contain a relatively high proportion of geminate stops, in contrast to the rest of the lexicon, where gemination was most prominent among resonants. This relative increase in geminate stops will in turn have led to an overall reinforcement of the status of geminates in the phonological system of Celtic languages.

## 10. Non-etymological gemination: accent-related or graphic gemination

De Bernardo Stempel ( 2010: 71–79) has observed that many words in ancient Celtic, especially in Gaulish, display etymologically unexplained gemination, in particular in posttonic position – assuming that her hypotheses about the placement of the accent on the penultimate syllable in Gaulish are accurate. It is not possible here to repeat all of the sub-types she discusses, but a few select examples shall suffice. ^11^ The putative position of the Gaulish accent will be indicated by an acute accent: *simiuisonna* ‘the name of a month’ < * *sēmi-u̯ēs-ón-ā* ‘half spring (?)’, *ogronno-* ‘the name of a month’ < * *ou̯gró-no-* ‘cold one (?)’; *uxello-* ‘high’ < * *uχsélo-* < * *upselo-*, cf. OIr. *úasal*, W *uchel*, Bret. *uhel* < * *ou̯χselo-*, apparently with the same suffix, but with a different ablaut grade in the root.

De Bernardo Stempel thinks of genuine phonological gemination that is dependent on the stress. While this is possible, I do not want to exclude the alternative possibility that, at least in a subset of the material, such spellings could be a merely graphic convention in order to replicate Gaulish accentuation on the penultimate syllable within the framework of Latin suprasegmental phonotactic rules, which only permit stress on the penultimate syllable when it is positionally long.

The reverse of this rule or tendency is the simplification or degemination of geminates in pretonic position ( De Bernardo Stempel, 2010: 79). A good candidate for this is the group of Gaulish names around *Biracos* (stress on *ā*) beside unsuffixed *Birros*, the latter being a likely cognate of OIr. *berr*, W *byr*, Corn. *ber*, Bret. *berr* ‘short’.

## 11. Non-etymological gemination: ‘symbolic’ gemination

### 11.1. ‘Expressiveness’

Another factor – psychological and therefore outside the domain of regular phonetic change – played a role in the increase of geminate sounds. A number of instances of geminates are found in words that have, or are believed to have, an affective or emotive quality. This category of gemination is commonly referred to as ‘expressive gemination’ ( *e.g.*, De Bernardo Stempel, 2010: 80; De Bernardo Stempel, 1999: 508‒521; Lühr, 1985: 275–276; Stüber *et al*., 2009: 260; Kuryłowicz, 1957: 132, 138, 142‒144; these references are not meant to be exhaustive). In some kinship terms an emotional involvement is evident, especially where the creation of a geminate occurs only in a single language, *e.g.*, OIr. *macc* ‘son, boy’ < * *mak ^u̯^k ^u̯^o-*, which contrasts with the older, ungeminated * *mak ^u̯^o-* that is continued in Gaul. *mapon*, W, Corn., Bret. *mab*; or W *geneth* ‘girl’ < * *genettā- vs*. Gaul. *geneta*, OIr. *geined* < * *genetā-*. Perhaps * *u̯raggā-* or * *u̯rakkā-* ‘old woman’ (see 6.2. (3)) and Gaul. *ninno-*, OIr. *nen* ‘handmaid’, OW *Nennius*, Bret. *nen(n)* < * *ninno/ā-* (?) ‘servant (?)’ can be included in this category as well. In these cases, there is no discernible phonological reason for the divergent development in just a single language. It is undeniable that a rule of arbitrary gemination, whether we call it expressive or hypocoristic, must have been synchronically operative at least in kinship terms. This was already evident in the words * *atta* ‘dad’ and * *mamma* ‘mum’, which have to be reconstructed with geminates even for Proto-Indo-European, a language that otherwise actively avoided geminate sounds. Gemination in personal names may be of a similar nature.

Interjections are a class of words that combine expressiveness with a performative aspect, which can find formal expression in gemination.

(1) A relevant example in Old Irish is *nacc*/ *naicc* ‘no’, probably from * *nak ^u̯^k ^u̯^e*. This contrasts with the related * *nak ^u̯^e* < prohibitive * *na* + the sentential connector * *k ^u̯^e*, which underlies W *na(c)*, Corn., Bret. *na(g)* ‘not’.

(2) Once-attested OIr. *upp*, W *hwff*, Bret. *ouf* ‘ooff’ could in theory be derived from a common pre-form * *upp*, beside * *up* > W *wb* ‘woe, alas’, but they can also be viewed as spontaneous utterances.

However, expressiveness as an explanatory strategy is often extended beyond these narrowly circumscribed cases to words where emotional involvement of the speaker is not easily discernible. For example, we may ask ourselves why so many Celtic adjectives for basic concepts such as ‘small’, ‘old’, ‘weak’ display geminates. Most of them cannot be captured by an emotion-based concept of expressiveness. Instead I propose to use the more neutral, descriptive term ‘symbolic gemination’. It takes its motivation from the concept of sound symbolism that refers to a perceived resemblance between the phonological structure of a word and its meaning. Sound symbolism can have a strong element of iconicity and onomatopoeia, but, like almost anything in language, a lot of the symbolism is arbitrary and can be triggered by culturally and grammatically specific conditions that, in the case of prehistoric languages, must remain obscure to us.

The etymologies discussed in this study so far comprise phonologically or morphophonologically motivated instances of gemination in inherited words (if we consider onomastic gemination as a morphophonological process). ‘Symbolic’ or ‘iconic’ gemination, on the other hand, is not triggered by phonetic cues, but is motivated by pragmatic considerations such as the semantics of words, or it has no discernible motivation at all – or at least none that is recoverable to us. It translates a special relationship, which speaker and hearer have to a concept, into linguistic markedness of words.

I am aware of the danger that any non-native classification of words as ‘symbolic’ may be arbitrary. I have therefore decided to restrict the category of symbolic gemination in Celtic to two types of words: onomatopoetic nouns (in fact a single example) and adjectives. Other scholars may apply different criteria.

### 11.2. Nouns

In one instance a reasonably good case can be made for sound symbolism, *i.e.*, onomatopoeia, namely OIr. *cloc* ‘bell’ < * *kloggo-*. Medieval languages outside of Celtic presuppose the variant stem * *klokkā-*, namely MLat. *clocca*, Fr. *cloch* and Germ. *Glocke*. W *cloch* (fem.), Corn. *clogh*, Bret. *kloc’h* (masc.) can either be compared directly with the Irish word, if the change * *gg* > * *χ* (see section 6.2.) is accepted, or they continue * *klokkā-*, either as a genuine variant or as a borrowing from Middle Latin.

### 11.3. Adjectives

One semantic class of words in which phonologically obscure gemination is noticeably frequent, are comparable adjectives, very often those for basic concepts such as physical qualities. My speculative approach to explaining this descriptive fact is that the iconic component of gemination allowed to give expression to uncertainty about the precise magnitude of those qualities, *i.e.*, it was an iconic way of ‘hedging one’s bets’ (in the same way as speakers of German tend to qualify adjectives by *irgendwie* ‘somehow’, in order not to commit themselves too strongly to a specific value). In addition to their symbolic or iconic component, most of the adjectives in this category are also notable for being etymologically opaque. Many of them are not only found in Insular Celtic, but have parallels in Gaulish. Even though the contexts in which they appear in Gaulish, namely preponderantly in personal names, do not allow us to determine their semantic content beyond all doubt, the formal correspondence with words known from Insular Celtic renders their interpretation as adjectives fairly certain. For practical purposes, the material will be arranged in groups that reflect their distribution. Some adjectives are found in several languages, and some are exclusive to a single sub-branch. In those cases that are found across several branches, borrowing between the branches can never be excluded.

***11.3.1. Inherited formations***

Only a subset of adjectives with geminates is of more or less good Indo-European inheritance. They are being repeated in this panorama for the sake of the overall argument, even though they have already been discussed in previous sections.

(1) PC * *dallo-* ‘blind’ > Gaul. *dallo-*, OIr., W, Corn., Bret. *dall*. As discussed in section 2.1. (5), it is attractive to explain this as PIE * *du̯l̥no-*, but the lack of preserved * *u̯* in the potential Gaulish examples is worrying. If the word is of Indo-European origin, its geminate can be explained through regular sound change from * *ln*.

(2) qPC * *dorro-* > Ir. *dorr* ‘harsh, rough’ and the abstract *doirr* ‘anger’. Both words are only attested late. An Indo-European etymology * *dor-so-* from the root * *der-* ‘to tear’ has been suggested, but such an explanation is by no means unavoidable.

(3) PIE * *g̑ʰerso-* ‘small, short’ > * *gerso-* > * *gerro-* > OIr. *gerr* ‘short (cropped)’.

(4) PIE * *kerso-* ‘cropped’ > * *kerro-* > OIr. *cerr* ‘crooked, maimed’ could belong to the root * *kers-* ‘to cut’.

(5) PIE * *korso-* ‘cropped’ > * *korro-* > Gaul. *corro-*, *coro-* in names, W, OCorn. *cor*, Bret. *corr* ‘dwarf’. This appears to be an ablaut doublet of the preceding item.

(6) qPIE * *kurso-* > * *kurro-* > OIr. *corr* ‘peaked, pointed; projecting part’, W *cwr* ‘corner, pointy edge’. This could be related to Lat. *curuus* ‘bent’, Gr. κυρτός ‘bulging, swelling, convex’, but the underlying root * *kur-* does not look Indo-European. De Vaan's ( 2008: 158) alternative suggestions for Lat. *curuus* eliminate a connection with the Celtic adjective. The Irish examples cannot always be distinguished from the preceding item.

(7) qPIE * *k ^u̯^elno-* or * *k ^u̯^elso-* > PC * *k ^u̯^ello-* > W, Corn., Bret. *pell* ‘far’.

(8) PC * *mallo-* > Gaul. *mallo-*, OIr. ‘slow’, W *mall* ‘evil, rotten, bad’ can come from PIE * *ml̥no-* or * *ml̥so-* ‘hesitant’ (?).

(9) qPIE * *polno-* ‘full’ > * *φolno-* or * *(h₂ ^?^)olno-* > * *ollo-* > Gaul. *ollon* ‘big?; whole?’, OIr. *oll* ‘great, ample’, W *oll*, *holl*, Corn., Bret. *oll* ‘all’.

(10) PIE * *(s)lh₂go-* ‘slack’ >> PC * *laggo-* > OIr. *lac* ‘weak’. If this word is of Indo-European origin, its geminate is unmotivated. Matasović ( 2009: 232) instead suggests PIE * *lh₂g-ko-*, but I am unaware of evidence for the postulated development * *gk* > * *gg*. See also Zair ( 2012: 59). The gemination may perhaps have arisen secondarily and be due to systemic pressure from the class of primary adjectives discussed below.

(11) PIE * *truCsmo-* > * *trummo-* ‘heavy, pressing’ > OIr. *tromm*, W *trwm*, Corn., OBret. *trom* ‘heavy’. A connection with PIE * *treu̯d-* ‘to thrust, press’ or PC * *truk-* ‘unfortunate, sad’ has been suggested (see Matasović, 2009: 391).

(12) qPIE * *(s)tug/k-slo-* ‘treated with a hammer or chisel?’ > * *tuslo-* > * *tullo-* > OIr. *toll* ‘pierced’, W *twll*, Corn. *toll*, Bret. *toull* ‘hole, perforation’.

One adjective has parallels in other Indo-European branches, but, because of its unusual structure, is believed to be of substratal origin:

(13) PC * *menekki-* ‘frequent’ > OIr. *menicc*, W *mynych*, MCorn. *menough* has irregular correspondences in Germanic * *mana/iga-* ‘many’ < * *mono/igʰo-* and Old Church Slavonic *mъnogъ* ‘much, many’ < * *munogʰo-*, and perhaps even in Finno-Permic, *e.g.*, Finnish *moni* ‘many’, Votic *mõni*, Northern Sami *moanak*, Udmurt *мында* ‘as much as’. This item is atypical for adjectives with gemination because of its disyllabic base.

***11.3.2. Common Celtic formations***

The following adjectives have no convincing extra-Celtic cognates:

(1) qPC * *biggo-* > OIr. *bec* ‘small, little’; OW *bichan*, W *bychan*, OCorn. *boghan*, MCorn. *byhan*, Bret. *bihan* ‘small, little’ continue either * *biggano-* or * *bikkano-*, with the suffix perhaps copied from PC * *φlitano-* ‘wide, broad’ < PIE * *pl̥th₂no-*.

(2) PC * *birro-* > Gaul. *Birrus*, OIr. *berr*, W *byr*, Corn. *ber*, Bret. *berr* ‘short’. The Gaulish adjective seems to have been borrowed as a technical term into Lat. *birrus* ‘a cloak to keep rain off’, Gr. βίρρος ‘cloak’, perhaps originally a ‘short cloak’.

(3) PC * *brikko-* > Gaul. *Briccus*, OIr. *brecc*, W *brych*, Corn. *brygh* ‘speckled’, Bret. *brec’h* ‘variola’. A connection with PIE * *perk̑-* ‘speckled’ has been suggested (LEIA B-82; Matasović, 2009: 78), but would be irregular. The early attestation of OIr. *brecc* with initial *br-* suggests that it is not etymologically connected with *mrecht* ‘variegated’ < * *mrek-to-*.

(4) qPC * *buggo-* ‘soft’ > OIr. *boc*, OBret. *buc*, *boc*, Bret. *bouk* ( *boug*).

(5) qPC * *burro-* ‘swollen’ > OIr. *borr* ‘swollen’, W *bwr*, OCorn. *bor* ‘fat, strong, big’.

(6) qPC * *gollo-* > OIr. *goll* ‘blind of one eye’. IEW 545 connects this *via* a ‘secondary voicing’ with “air. (acymr.?) *coll* ‘luscum, einäugig’”, a hapax in the Munich Sortilegia ( *Thes.* ii 236.3). I suspect the latter to be a misunderstanding of *coll* ‘loss, destruction’.

(7) qPC * *glikki-* > OIr. *glicc* ‘clever, ingenious, skilled’. Could there be a connection with *glacc* ‘hand, grasp, clutch’ < * *glakkā-*? For the connection of ‘cognition’ and ‘hands’ cf. Germ. *begreifen* and Lat. *comprehendere* ‘to touch with the hand = to understand’.

(8) qPC * *gozdo-*, * *gonto-* or * *goddo-* > MIr. *got* ‘stammering, lisping’.

(9) qPC * *kokko-* ‘red, scarlet’ > Gaul. *cocco-*, W *coch*, Corn. *coch*, *cough*. Possibly an ancient *Wanderwort*, cf. Delamarre ( 2003: 120–21).

(10) qPC * *kotto-* ‘old’ > Gaul. *Cottos*, OCorn. *coth*, Bret. *kozh*, and perhaps in the Old British ethnonym *Atecotti*.

(11) PC * *metˢamo-* (?) > * *messamo-* ‘worst’ > Gaul. *messamobi*, OIr. *messam*.

(12) qPC * *kittu-* (?) > Early ModIr. *cittach* ‘lefthanded, clumsy’. Unlike most of the other adjectives in this group this requires a stem in * *-u-*, if it is an old formation at all. Cf. Schrijver ( 2003: 4–5).

(13) If the Gaulish names *Suallius/a*, *Sualius* are cognate with Ogam Ir. SUVALLOS and OIr. *súaill*, *súail* ‘small, trifle’, they probably have to be analysed as compounds of * *su-* ‘good’ + an element * *u̯al(l)-i-* (in the case of OIr. *súail(l)* hardly identical with the root * *u̯al-* ‘ruler, to rule’). It is uncertain if the geminate *ll* is original or caused by individual secondary factors within each language.

(14) qPC * *tretti-* > OIr. *treitt* ‘quick, swift’; the more common form of the adjective, OIr. *traitt*, is perhaps influenced by the structurally and semantically similar OIr. *prapp*. W *trythyll*, *drythyll* ‘lively, high-spirited; lascivious’ and OIr. *treittell*, *dreittell* ‘pet, favourite; warrior’ could be derivatives from this. Alternatively *traitt* can be reconstructed as * *tratti-*, but it would need to be separated etymologically from *treittell etc*. in that case.

(15) qPC * *trikki-* > OIr. *tricc* ‘swift, active, sudden, urgent’.

***11.3.3. Exclusively Irish examples***

Several adjectives only found in Irish are ambiguous as to the precise reconstruction of the sound that synchronically in Old Irish looks like the descendant from a geminate:

(1) qPC * *u̯ozdo-* > OIr. *fotae* ‘long’. Together with Lat. *uastus* ‘vaste, immense’, IEW 1113 derives this from * *u̯asdʰo-* or * *u̯osdʰo-*. However, De Vaan ( 2008: 655–656) does not separate Lat. *uastus* from *uāstus* ‘desolate, waste’ < * *h₁u̯(e)h₂sto-* ‘empty’ (= OIr. *fás* ‘empty, void’), which is not a viable preform for OIr. *fotae*. In view of PC * *oNT* > PrimIr. * *oDD* (see section 7.1.), the reconstruction qPC * *u̯onto-* is also possible, as is an original * *u̯oddo-*.

(2) A mechanically reconstructed immediate preform of MIr. *láitir* ‘strong, powerful’ would be something like PrimIr. * *lāddiri-*. Perhaps the conspicuous fact that the degrees of comparison, *e.g.*, comparative *laiteri*, and the derived abstracts *láitire* and *láitirecht* show no syncope, is an indication that the preform was more complex, *e.g.*, * *lāde/idVri-*. No analysis suggests itself for any of those reconstructions.

(3) qPC * *menno-* or * *minno-* > OIr. *menn* ‘clear, visible’. The homonym OIr. *menn* ‘stammering, inarticulate, mute’ could have an identical preform. Since the word is not attested in contemporary Old Irish sources, it could conceivably also have been * *mend* < * *mendo-* or * *mindo-* originally.

(4) MIr. *prapp* ‘sudden, swift’ could be a language-internal neologism that makes iconic use both of the phonologically highly marked sound *p* and of gemination, or rather its reflex, *i.e.*, a voiceless unlenited intervocalic stop. Alternatively, it may reflect a symbolically reduplicated Latin loan, namely *rap-rapidus* (personal communication, Paulus van Sluis).

Where do the adjectives without Indo-European pedigree come from? What is notable about this collection of adjectives, which strongly relies on Irish, is that, apart from being formally united by gemination, they form several semantically coherent subgroups. They relate to basic physical concepts; to physical defects; and they are symbolic of swiftness and agitation. Gemination was phonologically original in the subset that continues Indo-European formations. From there, it can have spread as a semantic marker within the semantic field. External influence need not be invoked. It is evident that words such as * *biggo-* ‘small’, * *buggo-* ‘soft’, * *laggo-* ‘weak’ constitute a ‘phono-semantic’ class whose structure was self-replicating. That such a process was productive, is evident from words such as *prapp* ‘rapid, quick’, which, if it isn’t a substratal loan, can have been created sound-symbolically at a late date.

Another reason for ascribing these words to *Urschöpfungen* within Celtic, and not to loans from unknown languages, has to do with the typological tendency of adjectives to be less amenable to borrowing than nouns. The fact that such a number of conceptually central adjectives display gemination points rather to sound-symbolism as the driving force. Once gemination had been established as a phono-semantic marker for basic adjectives, there must have been enough internal momentum to create new material through this morphophonological process, without taking recourse to borrowings from a substrate.

## 12. Unclear gemination

A small number of nouns and verbs of certain or likely Indo-European origin possess geminates that cannot be explained straightforwardly from their putative preforms by phonological developments. Neither do these words manifest an affective character. For some of them, I will make tentative morphological proposals, usually involving some sort of derivational process that can account for the gemination. However, these attempts are merely speculative. In some words, the gemination has to remain unaccounted for for the time being.

### 12.1. nouns

(1) PIE * *h₁eh₂no-* ‘ring’ > * *āno-* → * *ānnii̯o-* > OIr. *áinne*. This item is related to Lat. *ānus*, Arm. *anowr*. Maybe the derivation involves the addition of the suffix *-no-*, *i.e.*, * *ān-no-*, to explain the geminate, or it is due to the syncope of a preform such as * *ān-in-i̯o-*. The lack of Osthoff shortening and the palatalisation speak in favour of the latter option.

(2) PIE * *pl̥keh₂-* ‘flat stone’ > * *φlikā-* → * *likkā-* > OIr. *lecc*, W *llech*, Bret. *lec’h*, Corn. *lehan*. The Gaulish placename *Arelica* appears to have a single * *k*; various Gaulish names starting with *licc-* could contain the etymon with a geminate. Perhaps the geminate is the result of a ‘precocious’ syncope between identical sounds in an adjectival formation of the type * *φliko-kā-* ‘stony’, which became substantivised and ousted the original simple noun * *φlikā-* ‘stone’, or the suffix was added directly to the root, *i.e.*, * *φlik-kā-*.

(3) The relationship among OIr. *ícc* ‘healing’ < * *īkkā-* and W, OCorn. *iach*, Bret. *yac’h* ‘healthy’ < * *i̯akko-* or * *i̯ekko-* is notoriously difficult, as is its extra-Celtic relationship, if there is any, with Myc. *a₂-ke-te-re*, *ja-ke-te-re*, Gr. ἄκος ‘cure, medicine’. Matasović ( 2009: 171) and Zair ( 2012: 68) tentatively and fully conscious of the formal problems think of * *i̯h₂ko-* as a starting point, but neither addresses the question of the geminate. Schrijver ( 1995: 103–104) dismisses the link with Greek and instead suggests, also tentatively, a formation * *i̯et-ko/ā-* from the root * *i̯et-* ‘to position onself’. Although it cannot account for the vocalism of the Irish word, it explains the geminate. Finally, I want to join in the tentative chorus myself. A possible source for the geminate could be a Proto-Celtic adjectival formation * *i̯ek-ko-* from the root * *i̯ek-* ‘to speak’. The semantic connection would be the premodern notion of healing through words of power. But I need to stress that aside from explaining the geminate, this etymology creates other issues with the initial vowel.

(4) qPC * *kruttā-*, * *krutto-* ‘bulging object, belly (?)’ > OIr. *crott* ‘harp’, W *croth* ‘womb, uterus, belly’, *crwth* ‘fiddle, violin; hump, hunch; anything of bulging shape’, Corn. *crothak* ‘abdomen, uterus’, Bret. *kourzh* ‘vulva’. MLat. *crotta* ‘harp’ looks like a loan from Irish. Lith. *krūtìs* ‘breast’ and Latv. *krùts* ‘hill, breast’ look related, but have a long vowel. A common preform could be set up as * *kruHto-* with laryngeal loss in Celtic. Perhaps it contains the PIE root * *kreu̯H-* ‘to pile up, cover’.

(5) qPC * *makko-* > OIr. *macc*, W *mach*, OBret. *meic* ‘bond, enforcing surety’. Perhaps * *magʰ-ko-* from the PIE root * *magʰ-* ‘to be able, powerful’?

(6) PIE * *pr̥keh₂-* ‘furrow’ > PC * *φrikā-* > Gall.-Lat. *rica* (whence Fr. *raie* ‘ray’), Bret. *regenn*. OIr. *etarche*, *eitrige* continues a compound with *etar-* ‘between’. However, W *rhych* ‘furrow’ and Bret. *rec’h* ‘grief’ (perhaps metonymically from ‘worry-lines on the forehead’?) presuppose PC * *φrikko-* with a seemingly unmotivated geminate. The difference in stem-class could indicate that this is in fact an adjectival derivative * *φrik-ko-*. Alternatively, those forms go back to a preform with a sigmatic suffix, *i.e.* * *φrik-so-*. Formal influence from * *φrikkā-* < * *pr̥d-keh₂-* ‘fart’ is unlikely.

(7) qPC * *truddo-* from the root PIE * *treu̯d-* ‘to push’ is set up in IEW 1095 as the preform of OIr. *troit* ‘fight, battle’, but neither word-formation, stem-class nor vocalism are satisfactorily explained by this. If an Indo-European connection is abandoned, the word can also be set up as * *troddV-* or * *trontV-.*

(8) PIE * *u̯ei̯h₁tV-* ‘winding (plant/thing)’ > * *u̯ei̯tV-*. OIr. 1 *féith* ‘kidney, fibre, sinew, vein’ and 2 *féith* ‘some kind of twining plant’ have been derived from this; if and how 3 *féith* ‘a swamp, marsh’ belongs here, is unclear ( Irslinger, 2000: 202–204; Matasović, 2009: 419; Zair, 2012: 230). W *gŵyth*, Corn. *gwyth* ‘vein, sinew’, OBret. *goed*, Bret. *gwazh* ‘valley, stream’ are evidently related, but continue * *u̯ei̯ttV-* with an unmotivated geminate. Bauer ( 2015: 66–67) tentatively suggests a borrowing from Irish into British.

### 12.2. verbs

Only a select number of Old Irish verbs will be included here.

(1) qPC * *u̯ekkā-* > OIr. *feccaid* ‘to bend, stoop; turn towards’. IEW 1135 derives this from the root * *u̯ek-* ‘to bend’ “mit expressivem *-kk-*”, but that root is now reconstructed as * *u̯enk-* with a nasal (LIV 683). Unclear.

(2) On the evidence of ModIr. *dímhigean* ‘contempt’, OIr. *do·meicethar* ‘to despise, condemn’ has a medial /g/. This precludes the reconstruction ?*mik⁽’⁾né/n-h₂-, tentatively proposed in LIV 429. Operating with the formation * *mi-n-k(h₂)-e/o-*, where the position of the infixed *n* would have to be secondary, results in PrimIr. * *migge/o-*. This explains the medial *g* of OIr., but leaves the stressed vowel *e* unaccounted for. W *edmygaf* ‘to admire, honour’ and Bret. *dismeg* ‘opprobrious’ < * *-mik-* seem to continue a formation without a nasal infix, unless all go back to a preform * *migg-*.

(3) Matasović ( 2009: 344) derives MIr. *scrípaid* ‘to scratch’ from PC * *skrībbā-* from the root * *skri(H)bʰ-* ‘to scratch’ (LIV 562: * *sk⁽’⁾rei̯bʰ-*). This is unnecessary, the Middle Irish verb may rather be a learned reborrowing of Lat. *scrībere* with unlenited *b*.

## 13. Gemination in potential loanwords

### 13.1. Possible loanwords

After having scrutinised the possible sources and the types of geminate consonants in the lexicon of the Celtic languages, the question to what extent geminate sounds can be relied upon as indicators for the substratal origins of words can finally be broached. This applies of course only to words for which no etymological explanation within the Indo-European paradigm can been found, and to words for which no case – how ever flimsical – for a sound-symbolic neologism can be made. Some words with geminates are evidently foreign, a case in point being * *katto-* ‘cat’, which is a trans-European *Wanderwort*, perhaps ultimately originating in a Bronze-Age Afro-Asiatic language such as Nubian. At the same time, this word is atypical for the substratum loans that we are interested in here since it refers to a foreign concept brought in from outside, not to one that was encountered *in situ* by incomers.

After having dealt in the preceding sections with words for which some sort of language-internal explanation can be found, we are left with a list of items that have no good etymology and no obvious source. This makes them suspect of being loans. The collection below expands on Matasović’s ( 2009: 441‒443) list of suspected non-Indo-European loanwords in Celtic, enlarged by additional examples that have been found in non-systematic searches. Some of the words have manifest parallels in languages outside of Celtic, but formal irregularities prohibit their reconstruction for the Indo-European protolanguage or even for a common Western Indo-European subnode:

(1) qPC * *ātti-* (?) > OIr. *áitt* ‘place’. A variety of Indo-European etymologies have been proposed for this word, all requiring the ‘Kluge’s Law’ treatment * *-tn-* > * *-tt-* (Pedersen, VKG i 161: * *pōthni-*; Klingenschmitt in Lühr, 1985: 303: * *ō-i̯et-nā*-; Bammesberger, 1998: * *pōtni-*). Since the validity of Kluge’s Law for Celtic is not accepted in this article, the word is only mechanically projected back to a possible Proto-Celtic pre-form, without further analysis.

(2) qPC * *bakko-* ‘angle, bend, hook, crook’ > OIr. *bacc*, W *bach*, LCorn. *bahaw*, Bret. *bac’h*. This has cognates in Lat. *baculum* ‘stick’, Germanic * *pag-* ‘peg’, which presuppose ungeminated sounds.

(3) qPC * *bekko-* > Gaul. *beccus* > Fr. *bec* ‘spout, muzzle’. Instead of continuing a variant * *beko-* without gemination, Bret. *beg* ‘spout, mouth’ is best understood as a borrowing from Gallo-Romance ( Piette, 1973: 80–81).

(4) qPC * *braddo-* or * *brazdo-* > OIr. *brat* ‘spoil, plunder, robbery’.

(5) qPC * *braddo-* or * *brazdo-* → OIr. *bratán* ‘salmon’. This etymon is perhaps identical with the previous, with a special semantic development from ‘a good catch’ > ‘salmon’.

(6) qPC * *bratto-* > OIr. *bratt* ‘mantle, cloak’, W *brethyn* ‘cloth’; the vocalism of Bret. *brozh* ‘skirt’ is irregular.

(7) PC * *brokko-* > Gaul. *Broccus* > Fr. *broche*, ogam BROCI, OIr. *brocc*, W, OCorn. *broch*, Bret. *broc’h* ‘badger’. The early Irish forms beginning with *br-* exclude a connection of this word with PC * *mrogi-* ‘land’.

(8) PC * *bukko-* > Gaul. *Buccus*, OIr. *bocc*, W *bwch*, OCorn. *boch*, Bret. *bouc’h* ‘he-goat’. Perhaps a loan from Germanic * *bukka-*.

(9) qPC * *bunno-* ‘owl, bittern’ > OIr. *bonnán*, W *bwn* ‘bittern’, Bret. *bonn* ‘crane’.

(10) qPC * *donno-* > Gaul. *Donnos* ‘?’, OIr. *donn* ‘chief, noble’. This is an uncertain item. The meaning of the Gaulish word is unknown and the Irish word could be the substanivised adjective *donn* ‘brown’ (see 3.1. (4)).

(11) qPC * *ētt ^s^(V)lūm(b)-* ‘bat’ > OIr. *íatlu*, W *ystlum*. See Van Sluis (forthc.) for the unusual reconstruction.

(12) qPC * *gaddā-* ‘theft’ > OIr. *gat* ‘theft’, Bret. *gad* ‘hare’. See Stifter (2021) for the semantic relationship between the two words.

(13) qPC * *garrV-* ‘calf of the leg, shank’ > OIr. *gairr*, MW, MBret. *garr*, W, Corn. Bret. *gar*. Matasović ( 2009: 152) suggests a connection with PIE * *g̑ʰesr-* ‘hand’, but this is semantically and formally not convincing since * *sr* doesn’t give * *rr* in Celtic (see section 3.4.). Alternatively he considers the possibility of a connection with PIE * *g̑ʰerso-* ‘small, short’, namely a zero-grade formation * *g̑ʰr̥so-* * *garso-* > * *garro-* ‘short one’, with the semantic evolution to ‘limb > shank’.

(14) qPC * *glakkā-* > OIr. *glacc* ‘hand, grasp, clutch’.

(15) PC * *gobann-* ‘smith’ > Gaul. *gobann-*, OIr. *gobae*, gen. *gobann*, W, OCorn. *gof*, MW pl. *gofein*, MBret. *goff*, NBret. *gov*. Beside the stem *gobann-*, which is attested in personal and divine names, Gaulish also has the stem *gobet-*. See Blažek (2008) for a formally not satisfying Indo-European derivation of this word.

(16) PC * *gobbo-* ‘beak, gob’ > OIr. *gop*; Gaul. * *gobbo-* is presupposed by Fr. *gober* ‘to slurp’, *gobelet* ‘goblet’.

(17) The nominal stem *illo-* or *illio-* is frequent in Gaulish onomastics, but neither its meaning nor its analysis are known. It can be formally compared with OIr. *ell* ‘flush, sudden emotion’, or *ell* ‘opportunity, advantage’.

(18) qPC * *kappilo-* ‘horse’ > OIr. *cappall*, dat.pl. *caiplib*, W *ceffyl*, OB *chefel*; Bret. *kefeleg* ‘woodcock’ is a derivative. Related to Lat. *caballus* ‘pack-horse’, but not a borrowing.

(19) qPC * *karreko/ā-* > W *carreg*, Corn. *carrag*, Bret. *karreg* ‘rock’; borrowed into OIr. *carrac*.

(20) PC * *kau̯anno-* ‘owl’ > Gaul. *cauannos*, Fr. *chouan*, *chouette*, W *cuan*, OBret. *cou(h)ann*, Bret. *kaouenn* (cf. Jørgensen forthc.).

(21) qPC * *kīmmukko-* ‘lobster’ > W *cimwch*, *ceimwch*, *gimwch*. It is most economical to assume that NIr. *gliomach*, *giomach* was borrowed from W *gimwch* at a comparatively late date.

(22) qPC * *kladdo-* ‘stone (?)’ > ModIr. *cladach* ‘(rocky) shore’, *cladar* ‘heap of stones’, *cladán* ‘fence-like pile of stones’, all with [d] (see Stifter, 2023a: 179 for a very speculative connection with * *kaleto-* ‘hard’ *via* a loan from British). Since the word is restricted to Goidelic, a reconstruction with * *zd* would also be possible.

(23) qPC * *knukko-* > OIr. *cnocc* ‘protuberance, hill’, W *cnwch* ‘protuberance’, OBret. *cnoch*, MBret. *cnech*, NBret. *krec’h* ‘hill’.

(24) OIr. *corr* ‘heron’ < * *korrā-* or * *kurrā-* < * *korsā-* or * *kursā-* could simply be a substantivisation of the adjective ‘hooked, pointed, humped’ (see 11.3.2. (6); thus Schrijver, 1997: 297) or it could continue a more complex preform qPC * *korχsā*-. The reconstruction with the complex cluster * *rχs* is prompted by the comparison with W *crychydd*, Bret. *kerc’heiz etc*. (Schrijver *ibid*.), which would require the presence of a guttural sound in the coda of the root. Proto-Germanic * *hraigran-*/* *higra(n)-* has a related shape, but ultimately the starting point for this word is probably onomatopoetic.

(25) qPC * *krettā-* > OIr. *crett* ‘framework’, W *creth* ‘nature, appearance, form’. The Old Irish oblique cases *creitte*, *creitt* point to the Proto-Celtic preform * *krettā-*, but * *krittā-* is not entirely excluded.

(26) qPC * *krokkeno-* > Gallo-Lat. *crocina* ‘mastruca, a garment made of skin’, OIr. *croiccenn*, W *croen*, OCorn. *croin*, MCorn. *crohen*, Bret. *kroc’hen* ‘hide, skin’. The palatalised *cc* of Old Irish must have spread from syncopated forms such as * *croiccnea*.

(27) PC * *k ^u̯^esno-* (?) > * *k ^u̯^enno-* ‘head’ > Gaul. *penno-*, OIr. *cenn*, W *pen*, Corn., Bret. *penn*.

(28) qPC * *luttā-* or * *lottā-* > OIr. *lott* ‘ruin, damage, injury’.

(29) qPC * *mekkVno-* > OIr. *meccon* ‘an edible root’. The word is occasionally written with double *nn*, *e.g.*, dat. *meccunn*. If this is old, the suffix could be similar to that of the ‘avian’ suffix * *-anno-* discussed by Jørgensen (forthc.). For speculations about this etymologically obscure word see Lühr ( 1985: 293–295).

(30) qPC * *met(t)o-* (?) > OIr. *meth* ‘decay’, W *meth* ‘failure, error’, Corn. *meth*, Bret. *mezh* ‘shame’. The irregular correspondence between the Irish and the British words suggests that one branch must have borrowed the word from the other. In the absence of an extra-Celtic cognate, establishing even the Celtic preform is impossible. If it was * *meto-*, Irish *meth* is regular and the word was borrowed into British (thus Bauer, 2015: 71–73). If it was * *metto-* with a geminate, it is the other way round. Because of its isolation, the word may be a substratal loanword, in which case the probability of * *metto-* increases.

(31) qPC * *nūsso-* > OIr. *nús* ‘beastings, milk of a newly calfed cow’. Perhaps borrowed into W *nus*, Bret. *luzenn*, unless the British word continue * *n*/ *lou̯sso-* with a different ablaut grade.

(32) W *pwll*, Corn. *poll*, Bret. *poull* ‘hole, pit; pool, pond’. OIr. *poll* ‘hole’ is evidently a borrowing; usually its source is believed to be Welsh. A mechanical back-projection results in qPC * *k ^u̯^ullo-*, which is phonologically implausible, since labiovelars were delabialised before * *u* in Proto-Celtic. A borrowing from OE *pól* ‘pool’ is phonologically and genetically unlikely: it cannot explain the vocalism of the Welsh and Breton words and a loan from Old English would not be expected to be present in all three British languages. Alternatively, it could be a substratal borrowing from the so-called ‘ *p*-language’, a pre-Celtic language postulated to have been present in Ireland as late as the first half of the 1 ^st^ millennium a.d. by Schrijver ( 2000; 2005; see also Stifter forthc.).

(33) qPIE * *rou̯kko-* > ModIr. *ruacán*, *rócán* ‘cockle’, W *rhuch* ‘bran, husks; garment’. The final *-t* of MIr. *rucht* ‘cloak’ is unexplained ( Stifter, 2023a: 186). A connection with the root * *h₁reu̯dʰ-* ‘red’ is unlikely; Germanic * *hrukka-* ‘cloak’ cannot be related because of the different initial.

(34) qPC * *skaddo-* ‘herring’ > OIr. *scatán*, W *sgadan*, *ysgadan*. This substratal word has also been borrowed into Germanic languages, *e.g.* OE *sceadd*, ON *skaddr* ( Schrijver, 2005: 141–143; Stifter, 2023a: 184).

(35) qPC * *slattā-* ‘rod, staff’ > OIr. *slatt*, W *llath*, LCorn. *lath*, Bret. *lazh*. This has cognates in Proto-Germanic * *laþ(þ)a/ōn-*, * *latta(n)-* ‘lathe’.

(36) Pre-Irish * *sliggii̯o-* or * *sleggii̯o-* > OIr. *slice* ‘(oyster)shell’. Alternatively, * *slinkii̯o-* is also conceivable ( Stifter, 2023a: 186).

(37) qPC * *tanno-* ‘green oak’ > Gaul. *tanno-*, Bret. *glastannen*. OIr. *tinne*, which is glossed as ‘holly, elder’ in some glossaries, should be kept separate. The word is perhaps only an artificial construct to press the ogam letter into an arboreal scheme. Formally and semantically, Germanic Old Saxon *danna* ‘pine’, OHG *tanna* ‘fir wood’ have to be kept apart.

(38) qPC * *u̯annellā-* ‘swallow’ > OIr. *fannall*, W *gwennol*, Bret. *gwennili*, OCorn. *guennol*. Gall.-Lat. *uannellus* ‘lapwing’ may represent the same etymon (see Stifter, 2010: 149–150). On the other hand Matasović, for whom the Celtic words for ‘swallow’ are inherited, reconstructs * *u̯ennālā-* < * *u̯esn-*, derived from the Indo-European word for ‘spring’. However, this reconstruction cannot explain the vocalism of the Irish word, nor of the Gaulish, if it belongs here. The different reconstructions at least agree in setting up a geminate * *-nn-* for the preform.

### 13.2. Assessment

We can now proceed to a provisional assessment of this collection. Most of these words are isolated and lack parallels outside Celtic. Where parallels exist, they are often in Germanic (but this may be partly a consequence of how the data was compiled). It is therefore conceivable that these words may have been borrowed from unknown languages in the prehistory of Celtic, in Western Europe or on the Western Archipelago. Unlike the adjectives in section 11.3., the borrowing of nouns is typologically a trivial phenomenon. The case for borrowing can be made more rigorously if it can be shown that the candidate words share specific phonological or morphological features apart from gemination. A number of relatively unspecific observations can indeed be made (I will occasionally also include words from previous sections in this discussion):

Many of these words are monothematic and monosyllabic (if we disregard the Proto-Celtic ending), where gemination is found at the end of the stem or root syllable. From a Proto-Celtic perspective, the geminate sound occurs in the onset of the second syllable that also contains the ending. Typical examples are * *bratto-* or * *gobbo*-. Examples with more than one syllable are * *ētt ^s^(V)lūm(b)-*, * *gobann-*, * *kappilo-*, * *karrekā-*, * *kīmmukko-*, * *krokkeno-*, * *mekkVno-*, * *u̯annellā-*; * *kau̯anno-* takes a special place since * *-anno-* can be isolated as a suffix (see Jørgensen forthc.).

Another feature is the preponderance of short vowels, or rather the absence of long vowels, in the root syllables (cf. Van Sluis forthc.). Including disyllables, the most common vowels are *a* (11) and *o* (6 or 7); *e* (5) and *u* (4 or 5) lag somewhat behind. Intriguingly, *i* is absent from the collection, unless the obscure Gaul. *ill(i)o-* is a substratal borrowing. This is in contrast to the adjectives in section 11.3., where *i* is not uncommon. Exceptions with long vowels are * *ātti-*, * *ētt ^s^(V)lūm(b)-*, * *kīmmukko-*, * *nūsso-*, items that are all only attested in the Insular Celtic languages. Maybe they reflect borrowings from a local prehistoric language in these islands. Perhaps items such as * *rou̯kko-* and * *u̯ei̯ttā-,* that have been mechanically reconstructed with a Proto-Celtic diphthong, can be referred to the group with long vowels as well. Among the words with geminate stops, the majority have voiceless sounds (the comparative dearth of geminate voiced stops was already highlighted by Martinet ( 1952: 198)). Exceptions are * *gaddo/ā-*, * *gobbo-*, * *skaddo-* and * *sle/iggii̯o-*. * *Braddo-*, * *kladdo-* and * *tru/oddo-* could be further instances, but since they are restricted to Irish, their * *dd* could in theory continue earlier * *zd*. * *Kloggo-* is a special case since it may be onomatopoetic. Among geminated resonants, preponderantly *nn* and *rr* are found.

The words have been preponderantly adopted as *o*- or *ā*-stems. Exceptions are * *ātti-*, * *ētt ^s^(V)lūm(b)-* and the consonantal stem * *gobann-*. The complex morphology of the words for ‘smith’ ( *e.g.*, Gaul. * *gobet-*) raises the question of a language-internal origin.

From the words included in the group with possibly Indo-European origin ( section 12.1.), * *krutto/ā-*, * *makko-* and * *tru/oddo-* would also fit formally into the group of potential loanwords. On the other hand, * *φlikkā-* and * *φrikko-* stand apart with their radical *i*.

One must never lose sight of the fact that the borrowings need not come from a single source. The formal difference between some of the words can be an indication that more than one donor language is involved. The phonetic shape of the extant words owes as much to the target phonology of the Celtic languages to which it had to be adapted, as it does to the donor languages. Depending on when the borrowing occurred, the donor language need not have possessed geminates at all: if, at the time of borrowing, the receiving language already had allophonic lenition of inherited intervocalic stops, any foreign unlenited intervocalic stop could have been perceived as a geminate.

## 14. The insular outlook

At the end of the developments described in the foregoing chapters, the phonological system of Celtic had morphed into the one in Table 5.

**Table 5.** the pre-Insular-Celtic sound system.

	plosive	nasal	fricative	glide	liquid
bilabial	b bː	m mː	(β)	w	
dental	t tː d dː	n nː	(ð)		l lː r rː
alveolar			s (z) tˢ		
palatal				j	
velar	k kː g gː		(x ɣ)		
labiovelar	kʷ kʷː? gʷ gʷː?				

Automatic, positional allophones are in brackets. This system reflects, in a somewhat idealised fashion, the state of affairs in the ancient Celtic languages and in the prehistoric Insular Celtic languages before the emergence of phonological lenition. For the descriptively *p*-Celtic languages, * *p* has to be substituted for * *kʷ*. The superscript question marks after the geminated labiovelar stops indicate their somewhat uncertain character. They cannot have been very numerous in the first place, but if they existed (for instance as a precursor to OIr. *macc* ‘son’), it is unclear if they were true geminates, *i.e.*, * *makʷkʷo-*, or rather sequences of velars + labiovelars, *i.e.*, * *makkʷo-*. The relative frequencies of geminate stops, especially of the voiced stops, may have differed between the individual Celtic languages.

### 14.1. Irish

After it had taken such a long time to build up a phonological system that had an opposition of length for almost every consonant, it is surprising to see how quickly this system disintegrated even before the full attestation of the medieval Celtic languages set in. Chiefly responsible for the quick abandonment of gemination, at least on the phonological level, was the rise of the opposition between unlenited *vs*. lenited sounds in the Insular Celtic languages. The emergence of lenition first as an allophonic feature of single stops in intervocalic position may well at first have been a side effect of the strong opposition between single *vs*. geminate consonants, caused by a maximum phonetic polarisation of the manner of articulation (see also Martinet ( 1952: 198–203, 215–216) for a more phonetically oriented account of the loss of gemination and for the systemic implications of gemination as a prominent phonetic class). However, after the contrast lenition : non-lenition had emerged as the central phonological opposition in Insular Celtic, the gemination of stops lost its phonological significance and turned into a phonetic feature that was concomitant with non-lenition, indicated by the bracketed length marks in Table 6 below. The old opposition geminated : ungeminated was translated into the new phonemic opposition unlenited : lenited only among resonants. The resulting phonemic system of Primitive Irish is shown in Table 6.

**Table 6.** the Primitive Irish sound system.

	plosive	nasal	fricative	glide	liquid
bilabial	b(ː)	m(ː)	β β̃	w	
dental	t(ː) d(ː)	nː n	θ ð		l: l r: r
alveolar			s		
palatal				j	
velar	k(ː) g(ː)		x ɣ		
labiovelar	kʷ(ː) gʷ(ː)		xʷ ɣʷ		
glottal			h		

For the ancient, medieval and early modern Gaelic languages, information about the phonetic status of the sounds can only be inferred indirectly from the written sources. The fact that single and double resonants were distinguished fairly consistently in manuscripts up to the modern period is an indication that the graphic distinction corresponded to a real-life phonetic distinction. At no point in the history of Irish can a comparable consistency be observed for the writing of unlenited obstruents. This is not the place to discuss the intricacies of Old, Middle and Early Modern Irish spelling conventions. Suffice it to say that, for example in Old Irish, the representation of word-internal unlenited stops by single or double letters appears to have been chiefly a matter of personal discretion of each scribe. This is exacerbated in later copies of Old Irish texts by the interference of a host of alternative spelling conventions for Modern Irish.

For the modern languages, the earliest recordings of traditional speakers allow some insight into the phonetic realisation of the sounds. For Irish, these are the recordings on wax cylinders by Rudolf Trebitsch in 1907 (kept at the Phonogrammarchiv of the Austrian Academy of Sciences) and the recordings by Wilhelm Doegen on shellacs, carried out between 1928–1931 (Royal Irish Academy; Conroy *et al*., 2009). Recent phonetic studies of the recordings of Donegal Irish in the Doegen collection shed some light on the phonetic length of unlenited sounds ( Wheatley & Iosad, 2021). It appears that in the early 20 ^th^ century, a contrast in length was still perceptible for some sounds, best preserved among resonants where the researchers noted perceptible length distinctions. In contrast, there was no significant length distinction in obstruents. These findings agree with the orthographic obervations made above.

### 14.2. British

The details of the prehistoric developments of geminated sounds are rather different in the British Celtic languages and can only be sketched here. A striking difference to Irish is the sound * *p* as the regular outcome of the labiovelar * *kʷ* in the phonological system of British Celtic. It seems that the voiced labiovelar * *gʷ* was reinterpreted as an allophone of * *w* and that the two occurred in complementary distribution. When the merger of * *w* and * *gʷ* happened is unclear.

The transformation of the Proto-British system ( Table 7) into that of the individual neo-Celtic languages Welsh, Cornish and Breton has been the subject of a long controversy (see the section on previous research in the introduction). From a structural point of view, the operation of intervocalic lenition on single stops removed single voiceless stops from intervocalic position for a short period. In response to this, a chain-shift was set in action in which gemination lost its phonological significance and became merely allophonic with non-lenition, or, in other words, geminate voiceless stops refilled the now empty phonotactical slot of simple voiceless stops. Geminated voiced * *b:* and * *d:* fell together with the new simple * *b* and * *d*, which themselves were the results of the earlier lenition of simple * *p* and * *t*. ^12^ The case of * *g:* is less clear. It may also have developed into * *g*, thereby falling together with the product of lenited simple * *k*, but in some cases it seems to have merged with * *k:* in yielding * *x* (see the discussion in section 6.2.). Unlike in Irish, there was probably never a stage where geminates occurred word-initially as allophones of unlenited sounds. See Van Sluis ( 2019: 30–35) for an account that differs in the details with regard to initial consonants.

**Table 7.** the Proto-British sound system.

	plosive	nasal	fricative	glide	liquid
bilabial	pː p bː b	mː	β β̃	w	
dental	tː t dː d	nː n	ð		l: l r: r
alveolar			s		
palatal				j	
velar	kː k gː g gʷ gʷː?	ŋ	(x) ɣ		
glottal			h		

The operation of syncope in Late Proto-British brought previously intervocalic obstruents and fricatives into contact with each other and thus created new complex consonantal clusters, which were prone to assimilation. The outcomes of this process called ‘provection’ in British historical phonology are voiceless stops, *e.g.*, the Welsh river name *Calettwr* < * *kaled ^†^đuβr* ‘hard-water’ < * *kaleto-dubro-*, Bret. *klopenn* ‘skull’ < * *klog ^†^benn* ‘stone-head’ < * *kloko-k ^u̯^enno-* ( Harvey, 1984: 98–99). By virtue of two segments coalescing into one, one could expect that the assimilation product was a long sound at first, at least phonetically and at least for a short period of time. Van Sluis ( 2019: 69–74) argues that word-external provection ( *i.e.* in sandhi) also produced long consonants up to Early Middle Welsh.

However, the ultimate result word-internally is a phonologically single, voiceless stop ( Harvey, 1984: 99). Provection triggered yet another chain-shift by which existing single voiceless stops, *i.e.*, the old voiceless geminates, underwent a new round of phonetic weakening when intervocalic inside a word, after resonants, or within accentual units. This weakening, which has the appearance of a ‘secondary lenition’ ( Greene, 1956: 289), but which is traditionally called ‘spirantisation’, resulted in the introduction of voiceless fricatives into the system, *i.e.*, old * *pp* > * *p* > * *f etc*. In order to grasp the bewildering aspects of the diachronic treatment of geminates in British Celtic it is quintessential to rigorously differentiate between phonology and phonetics. Different classes of sounds (voiced stops, voiceless stops, resonants) were degeminated first phonologically, then phonetically, at different times ( Isaac, 2004: 70). Like in the Gaelic languages, geminate resonants remained longest as a phonological class and their simplification only occurred during the historical period.

The system immediately before the emergence of the individual British languages was therefore the one in Table 8. (ː) indicates that length may have remained for some time as a phonetically concomitant feature, even though it played no role in phonology (cf. also Schrijver, 2011: 30–33).

**Table 8.** the late Proto-British sound system.

	plosive	nasal	fricative	glide	liquid
bilabial	p b(ː)	m(ː)	f β β̃	w	
dental	t d(ː)	nː n	θ ð		l: l r: r
alveolar			s		
palatal				j	
velar	k g(ː)	ŋ	x ɣ		
glottal			h		

## 15. Conclusions

Geminate consonants were a distinct and prominent phonological class in the history of the Celtic languages, namely in the attested ancient Celtic languages of the Continent, as well as in the reconstructable stages immediately before the attestation of the medieval Celtic languages in the insular world. Geminates also played an important role on the interface between phonology and morphophonology. The phonology of the historically attested Insular Celtic languages, on the other hand, has evolved into different systems where gemination as a phonetic feature is concomitant to other oppositions at best.

While sound change in inherited lexical items can explain the regular emergence of the core of this Common Celtic phonological class, it cannot account for all the types of gemination and for the entirety of the examples. By and large, a dichotomy is discernible in the Celtic lexicon between, on the one hand, words with geminate resonants and a ‘strong’ sibilant, and, on the other hand, words with geminate stops. Very crudely put, geminates of the first group tend to have good Indo-European etymologies, whereas those of the second group are less amenable to explanations along traditional Indo-European principles, unless they occur across a morphemic boundary especially in compound formations. In the latter, assimilations across the morpheme boundary contributed to the rise of geminates, but to different degrees in the different Celtic languages. The evidence especially of ancient Celtic personal names indicates that the various sounds showed a skewed distribution between the root and the suffixal part of words. This observation illustrates that gemination had acquired a morphological function in addition to its purely phonological status. Voiceless geminate stops were fairly common in Proto-Celtic, but voiced geminate stops are virtually absent from Proto-Celtic. Words in which the latter did not arise across morpheme boundaries, and in Goidelic as the product of the change *NT > *DD, are mostly either strongly suspect of being sound-symbolic neologisms or borrowings from unknown sources.

Although there is no clear-cut demarcation line between the groups, it is useful for analytic purposes to divide the material into one with phonologically explicable gemination and one with gemination that has no known diachronic source. In many of the steps outlined in this survey, gemination can be seen as a phonetic strategy to reduce the number of phonotactically admissable consonant clusters while retaining the moraic structure of the words. Etymological gemination is thus a compensatory reaction on the prosodic level to the loss of phonological segments. In the case of non-etymological gemination, two possible sources are conceivable: either they are loans from prehistoric substratal languages, or they are *Urschöpfungen*, *i.e.*, neologisms created within Proto-Celtic or in the individual languages. For typological reasons it has been suggested above that nouns rather belong to the first category, whereas adjectives belong to the second.

Over the long period of at least one and a half thousand years, a variety of factors conspired and various processes reinforced each other to slowly increase the number of types, and the number of tokens, of geminate consonants in the Celtic languages, but there was not just a single pathway to this. Some of the earliest steps in this direction constitute ‘uneven’ phonological change, when, for instance, * *sm* and * *ln* became * *mm* and * *ll* already in Proto-Celtic, whereas * *sn* and, perhaps, * *sl* did not immediately follow suit. The material assembled in this investigation also demonstrates that the emergence and treatment of geminated voiced stops went rather diverse paths in the Celtic languages.

In addition to sound changes that provide purely phonological factors for the rise of gemination, there are also psychological drivers, namely borrowing, symbolism and pragmatics. In previous scholarship, expressiveness has been claimed to be an important factor, but this has not proved to be a useful concept on any grand scale. While the emotional attitude of speakers clearly plays a role in kinship terms, that segment of the lexicon accounts only for a tiny portion of instances of gemination. After the systemic establishment of gemination as a phonological category, it may rather have been the concept of sound symbolism that occupied a central role in the further spread of gemination, in particular in the creation of adjectives describing physical reality. It is very rare that we find, as it were, ‘spontaneous’ gemination in words of Indo-European inheritance, *i.e.*, gemination that can neither be explained by phonological nor by psychological factors.

Because of the imbalance in documentation between Continental and Insular Celtic languages, it is difficult to assess if the frequency of gemination, especially of obstruent sounds, is higher in the latter than in the former, that is to say, if their frequency increased over time. However, it is noteworthy that geminated voiced stops are surprisingly rare, albeit not absent, in Gaulish. They may have been reinforced as a numerically substantial class only in Insular Celtic. Throughout the observable history and the reconstructable prehistory of Celtic, it is always the resonants that have been particularly prone to the creation of new instances of gemination (or rather ‘fortification’ during the younger stages of the Insular Celtic languages, when one can no longer speak of true geminates). However, when ‘full saturation’, as it were, of gemination was achieved in the Insular Celtic languages, it was quickly abandoned as a phonologically distinct class.

Of the data analysed in this study (excluding personal names), over 50 lack an obvious inherited, *i.e.*, Indo-European explanation. We may allow for the possibility that a subgroup of words with geminates was created language-internally through the operation of analogy and sound symbolism. This view is taken here especially for adjectives. However, the overall number of unetymologised words is too large to ascribe them entirely to those factors. It is suggestive that at least a subgroup of them, in particular nouns, were borrowed from prehistoric local precursor languages in Western Europe and in the Western Archipelago. Geminates are only one sign for substratal loans, and ideally they should go hand in hand with other markers for non-inherited origin, such as other unusual phonological and morphological features. The question to what extent the number of unetymologised words with geminates in Celtic is different (larger, equal, or lower) than in other languages of Europe, especially the Germanic (cf. Kuiper’s ( 1995: 68–72) source ‘A2’ for loans into that language branch) and Italic languages and Greek, goes beyond the present article and needs to be investigated in a broader, comparative context.

 In any case, from the foregoing it emerges that Celtic is an Indo-European branch that seems to contain a substantial body of words with geminate sounds borrowed from unknown substratal languages. The question imposes itself if this observation may be linked with the fact that in the Celtic languages intervocalic single stops underwent weakening through lenition, that is to say that *unlenited* single intervocalic stops of the donor language were *perceived* by speakers of Celtic as their only available unlenited intervocalic stops, namely geminated. The evidence for such a scenario is not clear-cut: Single intervocalic voiced stops were probably already allophonically lenited in Proto-Celtic. This would render foreign unlenited voiced stops particularly prone to be replaced by geminates in the borrowing process, but in fact voiced geminate stops constitute only a small minority among the words with unknown provenance in the Celtic languages. It is therefore best to assume that the donor language or languages did indeed have geminates.

## Ethics and consent

Ethical approval and consent were not required.

## Data Availability

All data underlying the results are available as part of the article and no additional source data are required.

## Footnotes

^2^ A noteworthy side aspect of Lühr’s study ( 1985: 300–302) is that she sets up geminate * *pp* for Proto-Celtic, which, unlike its simplex counterpart, she assumes to have been preserved. This differential treatment has an exact parallel in Japanese. A potential candidate is Gaul. * *lapparo-*, which can be postulated as the source of Fr. *lapereau* ‘bunny’, against Fr. *lievre* ‘hare’ < Lat. *leporem*.

^3^ There are other seemingly sporadic examples of *i* where *e* is expected, but none is parallel to * *in* in distribution. The 3 ^rd^ singular of the copula OIr. *is*, W *ys* must come from * *isti* < * *esti* < * *h₁esti*, but these seem to be independent developments in Goidelic and British Celtic. In Old Irish, the *i* is the result of raising of unaccented vowels before palatalised * *s* ( Griffith, 2016: 48–51), in Welsh it is an instance of *i*-affection of * *e* ( Hamp, 1974b: 33; Schrijver, 1995: 265–268). In Gaulish, PC * *esti* may in fact be attested as *esi* (L-98 1a9) with unraised *e*. On the other hand, the 1 ^st^ singular of the copula appears as *imi* (L-120), *immi* (G-13) in Gaulish, but since the corresponding Old Irish form *am* continues * *emmi* < * *h₁esmi* with unchanged vocalism, this raising must be specific to Gaulish.

^4^ *Pace* Dunkel ( 2014: 224) who derives it from * *en-dʰe*. The comparison of OIr. *indel* ‘preparation, machinery’ with Welsh *annel* ‘trap, deception’ < * *ande-lo-*, and Gaul. *ande-* leave no alternative than to reconstruct PC * *andi/ande* < * *n̥dʰi*.

^5^ The pretonic form *in·* could also be accounted for on the basis of * *en-* if influence of the main-clause particle * *et* is invoked, namely * *enet·* > *in·*.

^6^ This has been compared with Gaulish *incoro* in the inscription from Châteaubleau (L-93), but in view of the latter’s uncertain interpretation this comparison has to remain speculative.

^7^ The word seems to have /t/, not the /d/ expected from the proposed etymology, cf. ModIr. *itghe*, *itche*. Perhaps *itge* was influenced by the semantically related *attach* ‘invoking, refuge’ or devoicing occurred before *ch*.

^8^ Celtiberian *entor* and *entara*, and the Gaulish placename *Entarabo* ostensibly retain the old vocalism of * *entVr*; Late Gaulish *inter ambes* in Endlicher’s Glossary could be due to influence from Latin. Welsh *ythr* is ambiguous since preconsonantal * *en* was regularly raised to * *ın* in British.

^9^ Non-mutation furthermore occurs after words ending in *-r*, but this is marginal in comparison to the cases with final * *-s*. Aspiration is also found after * *-th* < * *-t*, if the Old Irish prepositions *la* and *fri* continue * *let* and * *u̯rit*. It is a moot point if the conjunct ending of third-person-singular verbs, which likewise ended in *-t* in the prehistory of Irish, would have caused aspiration. Since no evidence for it is known, this possibility will not be further pursued here.

^10^ As a side remark, it can be speculated if the Welsh suffix *-ach*, which occurs in the names of rivers such as *Afallach* (based on *afall* ‘apple-tree’) and persons, may have a similar explanation, namely an onomastic gemination of an older suffix * *-āko-* > * *-akko*- with concomitant shortening of the vowel.

^11^ The assessment of De Bernardo Stempel’s examples and of the various formal subgroups depends crucially on accepting her proposed etymologies, not all of which are uncontroversial.

^12^ In some positions, geminated voiced stops may have been retained as allophonic variants of simple unlenited stops. Especially in the case of Breton, phonetic, but non-phonological gemination was maintained and was still observed by researchers in Breton of the 20 ^th^-century ( Falc’hun, 1951; Greene, 1956: 285; Jackson, 1953: 545–548; Jackson, 1967: 317). Van Sluis ( 2019: 73) argues that such a situation obtained also in Welsh up to Early Middle Welsh.
