# Supplementary material for: The rise of gemination in Celtic
Source: Open Res Eur. 2024 Feb 8;3:24. Originally published 2023 Feb 2. [Version 2] doi: 10.12688/openreseurope.15400.2 (PMC10446051; doi:10.12688/openreseurope.15400.2)
Supplement: Peer review history

# Reviewer report: Reviewer response for version 2

**DOI:** 10.21956/openreseurope.18478.r38030

**Published:** 2024-06-08

**Authors:** Anders Jørgensen

**Affiliations:** 1 Disciplinary Domain of Humanities and Social Sciences, Faculty of Arts, Department of Archaeology and Ancient History, Uppsala University, Uppsala, Sweden

The author has taken the peer reviews into account when preparing the second version. I have nothing to remark this time around so nothing stands in the way.

If any, are all the source data and materials underlying the results available?

Yes

Is the work original in terms of material and argument?

Yes

Is the work clearly and cogently presented?

Yes

Is the argument persuasive and supported by evidence?

Yes

Does the research article contribute to the cultural, historical, social understanding of the field?

Yes

Does it sufficiently engage with relevant methodologies and secondary literature on the topic?

Yes

Reviewer Expertise:

Comparative and historical linguistics pertaining to Celtic (mainly Breton) and the Indo-European languages more broadly.

I confirm that I have read this submission and believe that I have an appropriate level of expertise to confirm that it is of an acceptable scientific standard.

# Reviewer report: Reviewer response for version 1

**DOI:** 10.21956/openreseurope.16648.r30786

**Published:** 2023-04-03

**Authors:** Elisa Roma, Gianguido Manzelli

**Affiliations:** 1 Dipartimento di Studi Umanistici - Sezione di Linguistica teorica e applicata, Università degli Studi di Pavia, Pavia, Italy; 2 Dipartimento di Studi Umanistici, Universita degli Studi di Pavia, Pavia, Lombardia, Italy

**Overview**

 This article presents consistent and comprehensive evidence to support a scenario of the development of the reconstructed consonantal phoneme inventories of Proto-Indo-European and Proto-Celtic that motivates the emergence of phonologically long (aka geminate) consonants in the Celtic branch and the later restructuring of length oppositions in Insular Celtic languages.

 The article argues extensively and mostly convincingly for the emergence of long consonants (predominantly resonants and secondarily voiceless stops) in Proto-Celtic mainly as a result of assimilation of consonant clusters, both original clusters and, notably, clusters arising through word-formation processes and therefore morpheme encounters. These developments are dealt with in detail distinguishing common phonetic outcomes within the Celtic branch from divergent outcomes of original clusters (Sections 3 and 4) and different chronologies and kinds of word-formation processes involved (Sections 5 and 6). The paper also highlights the relatively smaller evidence for long voiced plosives in Proto-Celtic and in earlier attested Celtic languages such as Gaulish, a fact which might be related to the divergent developments of voiceless and voiced stops in lenition contexts in the two Insular Celtic branches.

 The author suggests that reconstructible long consonants for which etymologies cannot be grounded on attested forms in Indo-European languages can be due to other factors, such as affective connotations and sound symbolism, especially in word classes and semantic fields where these phenomena are purportedly more frequent (address forms of kinship terms and personal names, adjectives denoting physical qualities). What cannot be arranged in any of these files is reasonably, albeit tentatively, ascribed to prehistoric borrowing (lexical copying) from unknown substratal languages. A merit of the article is that it thoroughly engages with the reconstruction and motivation of forms for which long consonants might be based on affective connotations, sound symbolism, and even borrowing (Sections 9, 11, 13), so that, in an attempt to avoid circularity, these residual groups are not just unaccounted for left-overs.

 Doubtful issues and problematic cases are not concealed (Sections 10 and 12).

 The comments that follow are therefore not to be meant as major criticims, but rather as suggestions that, it is hoped, may help improve the clarity and persuasiveness of the paper. They are grouped under three headings: 1) request for consistent terminology, 2) comments on specific points, 3) typos and minor slips.

 Reference is made below to pages in the pdf file and to numbered sections; abbreviations are the same as the paper’s, if not given in full.

**1) **In general, there are a few **terminological inconsistencies**, probably due to cumulative drafting, that could be addressed, to the reader’s benefit:

 I suggest that a single term, either “plosive” or “stop” or “occlusive”, be used throughout. If these terms are not synonyms in the author's usage, this should be clarified. Similarly for “fricatives” and “spirants” (e.g. p. 20 § 6.2 “spirant sounds”). “Obstruents” is yet another term that in some loci but possibly not throughout the paper is used for stops/ plosives/ occlusives (e.g. cf. p. 22 “a geminate voiced obstruent resulted”, p. 36 “intervocalic obstruents and fricatives”), while obstruent is in fact a term that includes both stops and fricatives.

 Either “velar” or “guttural” should be chosen (again, there does not seem to be any difference between their usage, so a single term would be preferable).

 For the mid-high front vowel in Goedelic, ɪ is used on p. 23, which is the IPA symbol for mid-high front (unrounded), while on p. 22 (§ 7 and § 7 (4)) a different symbol ɩ, possibly rather used for high central unrounded, is used instead.

 On a slightly different note, although the widespread terminology “mediae and mediae aspiratae” (p. 6) for the consonant inventory of PIE has a long tradition, it could be improved through greater consistency, allowing conversion with the terms used for the daughter languages and in general.

**2) Comments on specific points**:

 p. 4 (Preliminaries): “* *ballo-*, which is meant as equivalent to a phonetic analysis as [balːo-].” The phonetic transcription [balːo-] (rather [balːo]-) here seems to imply that a long consonant in a reconstructed form is not considered phonologically relevant (e.g. an allophone between vowels in this example); this is not generally the case, as reconstructed forms are rather closer to broad, phonemic transcriptions (put between slashes //). Anyway, this point should be better clarified. Also, it should be clarified if any stress and syllabic border is meant or implied in reconstructed forms. 

 p. 4 (Preliminaries): “The main contrast between the two classes of Celtic stops is considered to be between ‘voiced’ (= D) and ‘unvoiced/voiceless’ (= T) consonants”. It should be made clear from the outset (and not simply implied or taken for granted) whether the main phonological contrast between the two classes of stops (voiced vs. unvoiced) for the purposes of the paper is deemed corresponding, i.e. not a different opposition, to a phonetic contrast between singleton and long (= lenis and fortis respectively). This is not so, but what follows here seems to suggest the contrary.

 p. 4 (Preliminaries) and throughout: The label "quasi-" in qPIE and qPC is slightly misleading in this context, as it does not correspond to usual practice both for attested and reconstructed languages: in fact, modifiers distinguishing different phases usually point to chronological order (pre-, post-, early, late, old, middle, modern), while "quasi" refers to an approximation from either direction, or is rather synonymous with pre-. Therefore, quasiPC could be meant as a phase of late Indo-European approximating the separation of the Celtic branch. Clearer labels would be useful.

 p. 6, Table 1: p is inserted in the bilabial fricative case, but I suppose this is a slip: a fricative should be represented by a symbol for a fricative (e.g. for a voiceless bilabial fricative [ɸ]); [p] is a plosive that possibly lies behind a fricative consonant at this stage. This development from PIE has not been mentioned here before, however (see the paragraph listing the main changes in the consonant system from PIE to Proto-Celtic - beginning with “After” and ending with “Table 1 in Section 1”, p. 6). So, either p should be put in the right column (plosive) or fricativisation of the bilabial plosive should be mentioned and a fricative put in this slot in Table 1 (see § 2.1 (14) for an instance of this development).

 p. 7 § 2.1 (9) “* *salʲ†nʲī-*”: The use of the symbol † to signal the position where a vowel has undergone syncope in the prehistory of Irish should be mentioned.

 p. 8 § 2.2 (4) “Gaul. * *cammīnos*, which survives as a loanword in Ital. *cammino*, Fr. *chemin*, etc. ‘path, way’”: “as a loanword” sounds odd for a reconstructed Gaulish word that survives in French and Italian.

 p. 13 § 3.7 (2): “... for ‘eye’ is PIE * *suh₂lih₁* ‘two suns’”. Beside the translation, a clarification such as “the PIE dual form...” would be useful here, since when an inflected, non-nominative, non-singular (and non-masc. for adjectives) PIE form is given, its case/number (gender) must be specified.

 p. 14 § 3.9.1 (3): “constitutes the root of OIr. impersonal verb *do·cuäs* ‘has gone’.” It is rather an impersonal verb FORM, traditionally a singular passive (perfect) form, something which is different from an impersonal verb (i.e. a verb that is not construed with a nominative argument). The point is not totally irrelevant here since the Indo-European formation with * *-to-* lies regularly behind OIr. preterite/perfect passive forms. From the point of view of both their etymology and the Old Irish paradigms, the examples in § 3.9.2 (4) and § 3.9.5 (3) are the same; it is only the English translations that need to resort to "impersonality" with an intransitive verb such as 'to go'. Perhaps a different translation, anyhow, could be envisaged, e.g. 'there has gone', 'someone has gone'.

 p. 16 § 3.10 (3) “not ** *-aggo-* > OIr. ** *-ac*”: rather OIr. **-< *ac>* = -/ag/ (see § 4.1 for = /d/ through the stage *dd)

 p. 17 § 5.1 (3) “one can think of a formation from the root * *h₃rei̯H-* ‘to whirl’, perhaps from a hypothetical nasal-infixed verb * *rinati* ‘to run’, comparable with Goth. *rinnan* < * *h₃ri-n-H-*, with the addition of the adjectival suffix * *-no-* to the present stem. Alternatively, the formation could derive from the noun * *rei̯no-* ‘great flowing mass of water’ < * *h₃rei̯(H)-no-*, with ablaut and with the addition of a second nasal suffix.” The two alternatives appear to be highly speculative: can similar formations be invoked to support either hypothesis?

 p. 17 § 5.1 (6) “Old Irish has two distinct verbs that influenced each other formally: in PIE * *sn̥-n(e)-h₂-* ‘to obtain’ [...] PIE * *su̯n̥h₂-* ‘to sound’ > * *su̯ana-* acquired geminate * *nn* probably after its model, i.e., * *su̯anne/o-* > OIr. *seinnid* ‘to sound, make music’.” This kind of analogy at an early stage is frankly hard to believe, if the verbs were unrelated and, at the time when gemination was taken over by the second verb, length was distinctive for * *n*. Although mutual influence between the two verbs can be conceived, either a different explanation could be envisaged for geminate *n* in *seinnid* 'play music', or a later analogy should be invoked.

 p. 18 § 6.2 “An equation encompassing Gaulish and Irish...” Why "Gaulish and Irish" if Galatian is also involved?

 p. 21 § 6.3 (1) “Alternatively * *ku̯etur-* could be an archaism preserved in this compound, or *petor-* is simplified from the younger Gaulish form * *petu̯or-* with influence from the cardinal * *ku̯etu̯ores* ‘4’.” Since these alternatives are simpler, it would be better to mention them first, and then put forward the hypothesis which implies two successive metatheses.

 p. 22 (end of § 6.3) “There was a contrast between *s* and a strong sibilant...” If this is mirrored in the opposition between *s* and *ts*, it seems rather an instance of fricative vs. affricate. Otherwise, this point could be better qualified.

 p. 22 (end of § 6.3) “On the whole, geminated voiced stops were rare except across the morpheme boundary.” If that is so, it could be wiser to put geminated voiced stops on a different level as opposed to geminated voiceless stops: Table 2 puts all geminated consonants except nasals and liquids between brackets, which means that they are all considered allophones of the corresponding singletons, but if geminated voiced stops are so infrequent and limited to morpheme boundaries, one wonders whether they can be put on a par with the other allophones and should not be considered simply consonant clusters (where sub-phonemic alternations were rather between assimilated and non-assimilated clusters, than between short and long consonants). The point is that consonant encounters in word formation do not automatically create geminated allophones, but only (allowed) consonant clusters. The absence of true geminated allophones for voiced stops at this stage could be linked to their later divergent developments (§ 6.2).

 p. 22 § 7 “fell together as *é*.” Rather <é> = /eː/; as elsewhere, the spelling conventions of the different languages should not be taken for granted when describing phonetic changes. Therefore, also after “initial *éc-* and *ét-*”, add = /eːg/- and /eːd/-.

 p. 22 § 7 (4) *ceta-* is also a preverb with the meaning 'first' (perhaps more commonly), so a different example would be preferable.

 p. 23, fn. 3 “3 ^rd^ singular of the copula”: insert “present indicative”

 p. 24 Caption of Table 3: “preverbal 'in'” looks strange, as forms between single inverted commas are usually translations (is this what is meant here?)

 On a different note, the reconstructed forms in the Table are not straightforward, since allomorphy of the preverb is not clearly grounded on the basis of reconstructed contexts but rather on the basis of OIr. outcomes and in particular stress positioning. An independent reason, either phonetic or other, should be put forward for each previous stage allomorphy. Also, at this point it is not entirely clear how relevant the reconstruction of these allomorphs is for the main purpose of the paper. Since the discussion is meant to justify why * *in*+voiceless stop does not turn into *i/e*+voiced stop in OIr. in some cases, the starting point and the detour of the discussion should take a different stance.

 p. 24 fn. 5 This reconstruction assumes that * *et* is the particle that occurred in main clauses between preverb and verb-root and after a simple inflected verbal form. This option should be clarified.

 p. 24 (§ 7) “* *igg-* before a back vowel > * *egg-*.” Why a back vowel and not a lower non-front vowel? Does this occur with a following *u*?

 p. 25 § 8.1 “the outcome *f* (phonologically equivalent to a geminated * *u̯*!) is identical to that of the internal group * *-su̯-*, but is not phonetically similar to single * *u̯*, which rather gets lost.” It gets lost if internal, but not if initial.

 p. 26 § 8.2 While it is generally acknowledged that gemination did not play a role for the opposition between lenition and non-mutation, as it did in Goedelic, it is commonly held that it played a role for Welsh fricativisation of voiceless consonants (voiceless spirantisation; see the summary in Willis 2009 and § 14.2 in this article). At least some lines here about this development would clarify the picture and the author's stance in its regard and in relation to Section 14.2.

 p. 27 § 9.2 “in the root or in the semantically meaningful portion of the names”. Suffixes and endings are also meaningful (as all morphemes, by definition), so here it would be better to put the morphological predilection in different terms (e.g. semantically intense part?). Usually content vs. function word is used for lexemes, but that would not easily work for morphemes in name formation.

 p. 27 § 9.2 “in Gaulish short names or in names formed with suffixes.” This should be clarified: it is not entirely clear to what kind of names these are contrasted (names formed by composition, supposedly?).

 p. 27 § 9.2 “Dithematic names”. Does this mean compound?

 p. 27 § 9.2 “came to contain a relatively high proportion of geminate stops, in contrast to the rest of the lexicon, where gemination was most prominent among resonants.” This appears to be true for voiceless geminate stops, but not voiced ones (as per Table 4).

 p. 28 § 10 “The reverse of this rule or tendency is the simplification or degemination of geminates in pretonic position”. This simplification would not be warranted in the second scenario just mentioned, i.e. that gemination after penultimate stressed syllable is due to Latin stress position rules.

 p. 29 § 11.1 “restrict the category of symbolic gemination in Celtic to two types of words: onomatopoetic nouns (in fact a single example) and adjectives.” Cross-linguistic parallels for these kinds of expressive gemination would be useful for the sake of the argument.

 p. 29 § 11.3.1 “Only a subset of adjectives with geminates is of more or less good Indo-European inheritance. They are being repeated in this panorama for the sake of the overall argument, even though they have already been discussed in previous sections.” It is not entirely clear why forms where gemination emerges as the outcome of sound change are listed in this section where expressive gemination in adjective bases is deemed to occur and where one expects to find instances of adjectives where “phonologically obscure gemination” is identified (beginning of Section 11.3). Rather than supporting the overall argument, it puzzles the reader.

 p. 30 § 11.3.2 (9) “qPC * *kokko-*” This word (with geminate consonant) is attested in Greek and Latin (and attributable to Galatian, Freeman 2001: 16), and probably refers primarily to the kermes oak berry (Delamarre 2003: 120 ‘grain rouge, cochenille’), so one wonders whether it can be an instance of expressive gemination in adjectives. The development of the colour meaning is secondary.

 p. 30 § 11.3.2 (11) * *messamo-*. It is not entirely clear to me why this item is put here. Its formation is different from the other cases in this Section, as gemination in all likelyhood occurs as a result of word formation.

 p. 30 § 11.3.2 (12) “Early ModIr. *cittach* ‘lefthanded, clumsy’.” Are there no other related words?

 p. 31 § 11.3.2 (15) OIr. *tricc*. Should not this go in the following Section 11.3.3?

 p. 31 § 11.3.3 “They relate to basic physical concepts; to physical defects; and they are symbolic of swiftness and agitation.” Could taboos have played a role?

 p. 31 end of § 11.3.3 “Another reason for ascribing these words to Urschöpfungen within Celtic, and not to loans from unknown languages, has to do with the typological tendency of adjectives to be less amenable to borrowing than nouns.” This is a crucial point in the argument and therefore, although it is commonplace, it deserves non-trivial mention and updated references (e.g. Matras 2007, Matras & Adamou in press).

 p. 35 § 14 “* *makʷkʷo-*, or rather sequences of velars + labiovelars, i.e., * *makkʷo-*.” From a phonetic point of view, it is rather unlikely that secondary articulations like labialisation occurred twice, so to say. Generally, geminate consonants are produced with a longer constriction duration than singletons (Ladefoged and Maddieson 1996); phonological gemination of complex articulations such as affricates, for example, usually consist of a longer stop gesture with delayed release (e.g. [tːs]) rather than two successive articulations stop+fricative ([tsts], Ladefoged and Maddieson 1996: 92). Anyhow, typological parallels for gemination including two successive labialisations should be mentioned in support of the first option. (The articulation of Modern Irish palatal vs. neutral tense (long) resonants, in varieties where the length distinction is preserved, could also be compared, although this is not an exact phonetic parallel). [Note that this comment argues for the opposite view as Martin Kümmel's review. * *kk ^w^* is meant to stand in contrast to single * *k ^w^*, rather than to * *k ^w^k ^w^*]

 p. 35 § 14.1 “However, after the contrast lenition : non-lenition had emerged as the central phonological opposition in Insular Celtic, the gemination of stops lost its phonological significance and turned into a phonetic feature that was concomitant with non-lenition, indicated by the bracketed length marks in Table 6 below.” If the emergence of lenition “may well at first have been a side effect of the strong opposition between single vs. geminate consonants”, after the phonologisation of lenition, geminate stops could rather lose their phonetic status of long or tense consonants, but the effects of their earlier phonologisation were maintained and continued in fact in the phonological status of (non-lenited) stops. In other words, if the opposition between single vs. geminate stop turned into fricative vs. stop, this is not a real instance of de-phonologisation, as would be the case if it were maintained only in some environments, rather a re-phonologisation. This at least is in line with what is stated at the beginning of Section 8.1.

**3) Typos and minor slips**:

 p. 5 (previous research) Stokes > Stokes’

 p. 5 (previous research) assmilation > assimilation

 p. 10 § 3.1 (2) oder > or

 p. 20 § 6.2 (3) bye-form > by-form

 p. 22 (end of § 6.3) “in the system above”: Table 2 is in fact below.

 p. 22 § 7 allophon > allophone

 p. 30 § 11.3.1 (8) (OIr.) *mall* is missing

 p. 30 § 11.3.2 (6) acymr. > OW

 p. 30 § 11.3.2 (6) einäugig > one-eyed (?)

 p. 33 § 13.1 (10) substanivised > substantivised

 p. 36 (end of § 14.1) obervations > observations

If any, are all the source data and materials underlying the results available?

Yes

Is the work original in terms of material and argument?

Yes

Is the work clearly and cogently presented?

Yes

Is the argument persuasive and supported by evidence?

Yes

Does the research article contribute to the cultural, historical, social understanding of the field?

Yes

Does it sufficiently engage with relevant methodologies and secondary literature on the topic?

Yes

Reviewer Expertise:

Celtic Linguistics (Goedelic)

We confirm that we have read this submission and believe that we have an appropriate level of expertise to confirm that it is of an acceptable scientific standard.

## References

[1] : Dictionnaire de la langue gauloise: une approche linguistique du vieux-celtique continental. *Paris: Errance* .2003;

[2] : The Galatian language: a comprehensive survey of the language of the ancient Celts in Greco-Roman Asia Minor. *Lewiston, NY: Mellen Press* .2001;

[3] : The sounds of the world's languages. *Oxford: Blackwell* .1996;

[4] : The borrowability of structural categories. *In: Yaron Matras and Jeanette Sakel (eds.), Grammatical Borrowing in Cross-Linguistic Perspective. Berlin, New York: De Gruyter Mouton* .2007; 10.1515/9783110199192.31 31-74 10.1515/9783110199192.31

[5] : Word classes in language contact. *In: Eva Van Lier (ed.), The Oxford Handbook of Word Classes. Oxford: Oxford University Press* .In press; Reference source

[6] : Old and Middle Welsh. *In: M. J. Ball, and N. Müller (eds), The Celtic languages (2nd ed.). London, New York: Routledge* .2009;117-160

# Response

**Published:** 2023-04-10

**Authors:** David Stifter

Thank you very much for your many useful comments! David

# Reviewer report: Reviewer response for version 1

**DOI:** 10.21956/openreseurope.16648.r30783

**Published:** 2023-03-03

**Authors:** Anders Jørgensen

**Affiliations:** 1 Disciplinary Domain of Humanities and Social Sciences, Faculty of Arts, Department of Archaeology and Ancient History, Uppsala University, Uppsala, Sweden

The article provides a thorough treatment of the various origins of geminate consonants in the older Celtic languages. The article goes into great detail and provides explicit etymologies for a great number of words. As such, it will undoubtedly be used not only as a handbook of gemination in Celtic, but also as an etymological supplement for the Celtic languages for words which just happen to contain geminates.

 The article proceeds methodically, going through the various input clusters responsible for geminates in Celtic. The prose is straightforward and easy to follow. Of special interest is the discussion of possible substrate vocabulary, partially identifiable by geminates which do not allow further segmentation.

 While I have no difficulty giving the paper my full endorsement, there are a few minor things that might improve the text (page number refer to the pdf version of the article):

 p. 3: In the list of abbreviations, "OBret." is missing

 p. 4: Quoting Cornish from the GPC might not be ideal. It would be advisable to check the attested spellings in Graves (1962), The Old Cornish Vocabulary (for Old Cornish), Low (2022), A Dictionary of Middle Cornish (for Middle Cornish minus the more recently discovered texts) and George (2020), An Gerlyver Meur (for Old, Middle and Late Cornish).

 p. 4: Caution is advisable when quoting from Favereau's dictionary. He modernizes Old Breton (with accompanying phonetic transcription according to dialect!) and makes headwords out of material only *possibly* attested in e.g. place-names. It is always a good idea to check rarer words in Hemon's GIB and online in DEVRI.

 p. 6, § 2.1, example (4) and following: It might be a good idea to briefly mention Hill (2010-12). It relates directly to the discussion of words with syllabic *l+n* here and in the nasal presents below. If Hill is correct, the proposed reconstructions here will not work.

 p. 7, § 2.1, example (9): It may be worth mentioning the existence of MBret. *sallaff* 'to salt', adj. *sall* 'salted'. A derivation from French *saler* 'to salt' is possible (with Bret. *sall* 'salted' being back-formed from the verb). However, the Breton *-ll-* is surprising and the match to the proposed PCelt. form is intriguing. Could be cognate with OIr. *sall* instead.

 p. 8, § 2.2, example (3): It would be better to include Middle Breton *cam* 'step' here instead of the Modern Breton derivative (especially since the Breton suffix *-enn* may cause confusion for the reader).

 p. 10-11, § 3.1, example (8): The lack of syncope seems to be secondary if the reconstruction in the preceding entry is to be trusted.

 p. 11, § 3.1, example (13): Reference to the discussion of Egurtzegi and Ariztimuño (2013) would be suitable here, since it appears to eliminate some of the evidence in favour of substratal status.

 p. 14, § 3.9.4: delete hyphen in the heading.

 p. 16, § 4.2, example (4): Does a Modern Breton *izhin* actually exist?

 p. 17, § 3.7, example (3): W *melltith *is not a good example of the borrowing of Lat. *-ld- *as Brit. * *‑llt‑ *(shouldn’t this rather be Brit. * *-lt-*?). As shown by MBret. *malloez < maledictiō, millizyen* *< maledictiōne-, *Bret. *millig-, *MCorn. *mylyg-* *< maledīc-, *Welsh *melltith* is probably secondary, with learned *‑d‑ *(devoiced by the preceding *‑ll‑*), as also seems to be the case with MBret. *bennoez, binizyen <* *benedictiōne-, binnig-* *<* *benedīc- *as opposed to W *bendith, bendig-*. Note that the medial cluster in *melltith*, if one really takes it at face value, cannot have merged with older * *‑lt‑* (from PCelt. * *-lt- *and Latin *-lt-, -ld-*), because this gives MW *‑ll- *medially, cf. e.g. * cal’dāria > *kaltɔr > MW *callawr, *MBret. *cauter.*

 This leaves us with *mellt *as the only reasonably solid example of preserved * *‑ld‑ *(as Brit. * *‑lt‑*). Given that we have examples of vacillation between *‑ll *and *‑llt *in Middle Welsh (MW *gwellt *next to OW *guel, *MW *guell, *MW *gwyllt *next to MW *guyll, gwyll, *MW *deall *next to *dallt*), it might rather be the case that the final *‑t *in *mellt *is unetymological.

 p. 18, § 6.2: Breton *a-dreñv* means 'behind'.

 p. 20, discussion of the outcome of voiced geminates in Brit.: Allowing an analogical treatment of clusters across morpheme boundaries may allow us to treat the outcome of *zd as having passed through the stage *dd, only to become devoiced to *tt and spirantized and thus being part of a general devoicing of voiced geminate clusters (cf. Jørgensen 2022). This requires that e.g. Bret. *gad* is a borrowing (presumably from Irish).

 p. 21, example (4): It would make sense to standardize Breton *gwrec'h*, *grec'hent* to either forms in *gwr-* or *gr-*.

 p. 30, § 11.3.2, example (1): It seems much more likely that the *-an* in *bychan, *etc. is the diminutive suffix, cf. the meaning.

 p. 30, § 11.3.2, example (4): This contradicts the change of *gg > *kk > *x presented earlier in the text. I have suggested (Jørgensen 2022: 145) that the actual reflex of *buggo- in Breton is *bouc'h* 'blunt' and that *bouk* is a borrowing from Irish.

 p. 33, § 13.1, example (26): It may be better to leave out W *croen* and OCorn. *croin*, since they cannot continue *krokken- directly anyway, whatever the explanation. Also, as far as I can see, a reconstruction *krokkenno- would fit both Irish and Breton better (MCorn. *crohen* tells us nothing and Gallo-Lat. *crocina* does not fit anyway and may have suffix substitution). The word consistently rhymes as having /-enn/ in Middle Breton and is mostly spelled in accordance with this. It does not otherwise behave like a singulative in *-enn*, forming its plural with internal *i-*affection, so it does not appear to have been influenced by this suffix.

If any, are all the source data and materials underlying the results available?

Yes

Is the work original in terms of material and argument?

Yes

Is the work clearly and cogently presented?

Yes

Is the argument persuasive and supported by evidence?

Yes

Does the research article contribute to the cultural, historical, social understanding of the field?

Yes

Does it sufficiently engage with relevant methodologies and secondary literature on the topic?

Yes

Reviewer Expertise:

Comparative and historical linguistics, pertaining to Celtic (mainly Breton) and the Indo-European languages more broadly.

I confirm that I have read this submission and believe that I have an appropriate level of expertise to confirm that it is of an acceptable scientific standard.

## References

[1] : A Dictionary of Middle Cornish. *Cardiff: Goldcrest Publications* .2021;

[2] : An Gerlyver Meur. *Kesva an Yeth Kernewek, Cornish Language Board* .2020;

[3] : The Old Cornish Vocabulary. *Columbia University PhD dissertation* .1962;

[4] : Silbische Liquiden vor Nasalen im Inselkeltischen und das Problem der Nasalpräsentien vom Typ air. sernaid, kymr. -sarnu. *Keltische Forschungen* .2012;5:157-184

[5] : Celtic.2022; 10.1017/9781108758666.009 135-151 10.1017/9781108758666.009

[6] : Remarks on the Etymology of the Basque Word for ‘Swallow’ and Its Potential Relation to Celtic. *ÉRIU* .2013;63(-1) : 10.3318/ERIU.2013.63.79 79-90 10.3318/ERIU.2013.63.79

# Response

**Published:** 2023-03-08

**Authors:** David Stifter

Thank you very much for your very helpful comments!

# Reviewer report: Reviewer response for version 1

**DOI:** 10.21956/openreseurope.16648.r30784

**Published:** 2023-02-22

**Authors:** Karin Stüber

**Affiliations:** 1 University of Zurich, Zurich, Switzerland

Geminate consonants in lexemes attested in Celtic languages have been used in the past as cues for non-Indo-European origins of the words in question and in consequence for their status as borrowings from substrate languages. These alleged loanwords, in turn, were sometimes used for conclusions on phonological properties of those substrate languages.

 The article discusses the historical phonology of geminate consonants in the Celtic languages. In a systematic fashion the evidence for etymologically justified geminates, arising by assimilation of consonant clusters, is presented first for Proto-Celtic and then for the individual Celtic languages. This is followed by a chapter on non-etymological gemination, e. g. in onomastics or with expressive force.

 The article offers a very useful collection of all the relevant material and a thorough discussion thereof. Finally, the question whether geminates can be a marker of substrate loan words is addressed.

 The author's conclusion is that, broadly speaking, geminate resonants (like ll, rr) are usually phonologically justified in inherited lexemes, whereas words containing geminate stops tend to be etymologically obscure. In the case of nouns, there is good cause to understand them as loanwords. On the other hand, the author identifies a number of adjectives describing physical properties containing geminate stops. In their case, he argues that those geminates might have arisen for onomatopoetic reasons.

 In my view, the article is a valuable contribution to Celtic phonology. The idea of taking word class into account when considering phonological properties, when transferred to other languages, promises interesting results

If any, are all the source data and materials underlying the results available?

No source data required

Is the work original in terms of material and argument?

Yes

Is the work clearly and cogently presented?

Yes

Is the argument persuasive and supported by evidence?

Yes

Does the research article contribute to the cultural, historical, social understanding of the field?

Yes

Does it sufficiently engage with relevant methodologies and secondary literature on the topic?

Yes

Reviewer Expertise:

Historical linguistics, Celtic linguistics

I confirm that I have read this submission and believe that I have an appropriate level of expertise to confirm that it is of an acceptable scientific standard.

# Response

**Published:** 2023-02-22

**Authors:** David Stifter

Thank you very much!

# Reviewer report: Reviewer response for version 1

**DOI:** 10.21956/openreseurope.16648.r30785

**Published:** 2023-02-16

**Authors:** Martin Joachim Kümmel

**Affiliations:** 1 Friedrich-Schiller-Universität Jena, Jena, Germany

The paper treats a topic never systematically treated as such, and so it is by definition innovative, even if much of its content takes up earlier research concerning individual points. The question it tries to answer follows from the absence of geminates in the Proto-Indo-European ancestor of Celtic and the existence of geminates in Celtic languages: Geminates must have been introduced by phonological changes somewhere in the (pre)history of Celtic, so where did they come from? The same question must be asked for every other branch of Indo-European that developed geminates, and it has not been investigated systematically for all of them, and so this article could serve as a model for other branches.

 The main source for geminates was assimilation of different consonant clusters, and so the article discusses a large number of such clusters and their development in Celtic; it also includes a discussion of the outcome of *i preceding assimilated *NT-clusters in Goidelic, arguing for a variant *in beside *en,  *eni, *ande for „preverbal *in*“. It is shown that gemination of resonants was probably the first and arose by assimilation of some clusters already in Proto-Celtic (PC). In addition, some clusters with stops also appear to have been assimilated everywhere and thus possibly in PC, namely *ndn, *nnd and even * *dk*. Many more changes cannot be ascribed to Proto-Celtic but belong to the parallel histories of Celtic subbranches and show similar tendencies after their separation. In addition to the results of various assimilations, cases of „onomastic“ and „expressive“ gemination and the impact of possible borrowings are discussed. It is shown that different factors "conspired“ for the rise of geminates, including support from unknown substrate languages which probably had geminates.

 Previous research is adequately discussed, and the argumentation is generally sound; unclear cases are named as such, and dubious proposals are rightly rejected. Of course, as always in such comprehensive overviews, not all cases can be discussed in all details but there are no serious shortcomings.

 Remarks on single points are given under the heading numbers of the original paper.

 1.  It remains questionable if "strongly affective“ words with geminates can really be "projected back to the protolanguage“. The problem, as already shown by the Celtic examples, is that such words do not follow the sound laws, and hence it is impossible to demonstrate that they must be inherited. Instead, the correspondence between Gothic *atta*, Gaulish *atta/att* *ū*, Hittite *atta-* etc. does not presuppose a common ancestor *attV-, as little as its similarity to Common Turkic *ata* presupposes a common ancestor. By the way, the Hittite word does not necessarily have an original geminate *tt (distinct from single *t), since *tt* would be the outcome of *t anyway, and the Germanic word, being an *n-*stem, could easily owe its geminate to Kluge’s Law. In the light of „onomastic“ gemination (section 9), it seems equally possible that a potential PIE word still had *t which underwent gemination only *einzelsprachlich* (but not, e.g., in Slavonic *at-ika-).

 2.1 What exactly is the process of "analogical introduction“ of consonantal gemination after the model of one suffix? Was this a proportional analogy *-lo- : *-llo- = *-ko- : X?

 2.2 For the examples with *T-s-m° in suffixes, it might be made clearer why the individual cases must be reconstructed with *s and not simply with *T-m°, since the *s is never directly visible nor supported by parallels in other branches.

 2.3 (1-2) OE *pint-*, MLG *klint* need not presuppose *nd- if my proposal of some special developments in Germanic NT-clusters (Kümmel 2017) is correct which would allow to explain Germanic *nt also from *nd *ʱ*; alternatively,  an explanation through Kluge’s Law would be possible, viz. *glend *ʱnó-* > Germ. *klentta- > *klinta-.

 2.4 (1-4) Original *dk was apparently not assimilated but just simplified to *k in decade formations like PC *wikant *ī* ‘20’, *trikont- ‘30’ < (q)PIE *(h₁)wi-dk̑ m̥tih₁, *tri-dk̑omt-, where Indo-Iranian has *nk̑ (Sanskrit *viṃśatí-*, *triṃśát-*; Ossetic Digoron *ins* *æj, ærtin*) while other IE languages (with slightly different formations) show loss of *d with compensatory lengthening of the vowel (e.g., Latin *uīgintī*, Greek Doric *wī́kati*) if they continue such a form or sequence (unlike remade forms like Greek *triā́konta*). So it seems that the assimilation in words formed in Celtic follows a new pattern here.

 (5) The difference of *dk > *kk and *tk > *kt, *dg > *gd is striking but it might be useful to address the question of a conditioning for the metathesis in *arxto-, *gdonjo- (and *gdū ‘earth’). Maybe just *dk  > *tk, followed by *tk > *kk* /V_V (but not *dg > *gg?) and *tk, *dg > *kt, *gd /#,C_?

 3.4 The example of *tisr- with some segmental reflex of * *s* in Celtic and the other examples with complete loss do not show a uniform treatment, or do they? This might be made clearer.

 3.7 (1) There might be an alternative etymology for *gwell* etc., from a *wel-root, cf. Germanic *wel- ‘good’ etc. (so Matasović 2009 s.v.).

 3.9 For the intermediate stage * *t ^s^* to yield later *ss*, it must have been either *ts or *tt ^s^, so one of these should be written instead; otherwise it would have been monomoraic and become bimoraic again.

 3.9.5 For the development of * *st* > *ss* an intermediate stage [ts] is unnecesssary, so its asumption depends on (Gaulish) attestations of *ðð/θ* etc. Are there examples other than *tuððos/tuθos* for this? In any case, such a development in Gaulish does not necessarily imply that Insular Celtic had the same metathesis.

 3.10 Fn. 2 If Proto-Celtic really had (acquired and) preserved *pp in contrast to simple *p, wouldn’t it be useful to include this possible (but very rare) phoneme in the phoneme tables?

 4.1 Would it be possible to set up a relative chronology by which *zd > *dd > British (and Gaulish) *tt was earlier than the rise of younger *dd (which was not devoiced)? Devoicing of geminates is a rather natural change (and probably part of Kluge’s Law in Germanic), and the examples of a voiceless outcome under 6.2 seem to show the same, and might be older, as discussed there.

 6.1 What could be the reason why original *dʱsdʱ > *dzdʱ does not develop like original *tst > Celtic *ts? With only one example, some kind of analogical influence is difficult to exclude.

 6.2 Here the question arises what kind of "morpheme boundaries” are meant: formations with prefixes are originally compounds (at least they are in Sanskrit where external sandhi is applied), so it is actually boundaries of phonological words, not (word-internal) morphemes. Could this make a difference for the outcome of clusters, and would it be possible to assign conflicting results to full vs. incomplete univerbation?

 11.3.1 (13) The vocalism of Slav. *munaga- has been argued to be regular from a zero grade (Matasović 2004) - still not directly comparable to Celtic *men- and Germanic *man- but not really ”irregular”.

 14. There is hardly any real difference between *kʷkʷ and *kkʷ, as it is quite improbable that it was actually pronounced without lip rounding in the first half of the long [k]-gesture and with rounding in the second, and it is even more improbable that such a [kkʷ] could stand in contrast to [kʷkʷ].

 14.1 The recordings of Trebitsch have been published in Lechleitner & Remmer (2004), so this edition should be cited here.

 14.2 Tables 7 and 8: Why is labial-velar /gʷ/ present in table 7 but gone in table 8? Is there not still a unlenited : lenited correlation with /w/ in the attested languages, more or less like /b/ : /β/? Probably, the glide should be classified as labio-velar rather than „bilabial“,  and one may also consider adding another labio-velar phoneme /hʷ/ (cf.  Primitive Irish /xʷ/), especially since this possibly does not always go back to a cluster * *hw* (at least according to Jørgensen 2012).

 The relative length of voiceless obstruents may be argued to have remained relevant until the split of vowels (reflected) in (Modern) Welsh into a long/tense in originally open syllables (preceding simple voiced consonants and some fricative-stop clusters) and short/lax variant in  originally closed syllables (preceding clusters, original geminates, voiceless consonants and *m, ng*), so it must have played a role in phonology.

If any, are all the source data and materials underlying the results available?

No source data required

Is the work original in terms of material and argument?

Yes

Is the work clearly and cogently presented?

Yes

Is the argument persuasive and supported by evidence?

Yes

Does the research article contribute to the cultural, historical, social understanding of the field?

Yes

Does it sufficiently engage with relevant methodologies and secondary literature on the topic?

Yes

Reviewer Expertise:

Indo-European linguistics; historical phonology; phonological typology

I confirm that I have read this submission and believe that I have an appropriate level of expertise to confirm that it is of an acceptable scientific standard.

## References

[1] : Palatalization of *sk in British Celtic. *In Nielsen Whitehead, B; Olander, T; Olsen, BA; and Rasmussen, JE (eds.), The Sound of Indo-European. Phonetics, Phonemics and Morphophonemics. Selected papers from the conference held in Copenhagen, 16-19 April 2009 (Copenhagen Studies in Indo-European 4). Copenhagen: Museum Tusculanum* .2012;209-222

[2] : Das dünkt mich dunkel: Germanische etymologische Probleme. *In Hansen, BSS; Olander, T; and Olsen, BA (eds.), Etymology and the European lexicon. Proceedings of the 14th Fachtagung der Indogermanischen Gesellschaft, 17-22 September 2012, Copenhagen. Wiesbaden: Reichert* .2012;219-233

[3] : Celtic Recordings - Ireland, Wales, Brittany, Isle of Man and Scotland (1907-09). (Sound Documents from the Phonogrammarchiv of the Austrian Academy of Sciences. The Complete Historical Collections 1899 - 1950. Series 5/2: The Collections of Rudolf Trebitsch. OEAW PHA CD 14). *Wien: Verlag der Österreichischen Akademie der Wissenschaften* .2004;

[4] : The Proto-Indo-European syllabic resonants in Balto-Slavic. *Indogermanische Forschungen* .2004;337-354

[5] : Etymological dictionary of Proto-Celtic (Leiden Indo-European Etymological Dictionary Series 9). *Leiden/Boston: Brill* .2009;

# Response

**Published:** 2023-02-16

**Authors:** David Stifter

Thank you very much for this helpful and insightful report, David
